# Supplementary material for: A Hidden Markov Model for Detecting Confinement in Single-Particle Tracking Trajectories
Source: Biophys J. 2018 Sep 13;115(9):1741–54. doi: 10.1016/j.bpj.2018.09.005 (PMC6226389; doi:10.1016/j.bpj.2018.09.005)
Supplement: Document S1. Supporting Materials and Methods, Figs. S1–S20, and Tables S1 and S2 [file mmc1.pdf]

Biophysical Journal, Volume *115*

Supplemental Information

A Hidden Markov Model for Detecting Confinement in Single-Particle Tracking Trajectories

Paddy J. Slator and Nigel J. Burroughs

Supporting Material: A Hidden Markov Model for Detecting Confinement in Single Particle Tracking Trajectories

PJ Slator, NJ Burroughs

1 Note S1: MCMC algorithm for harmonic potential confinement HMM

We implemented the following MCMC algorithm in Matlab. The source code, documentation, trajectory data, and working examples are freely available (doi:10.5281/zenodo.1405647).

For a 2D trajectory $\mathbf{X} = \{X_i, t_i\}_{i=1}^{N+1}$ the switching harmonic confinement model with parameters $\{\kappa, D, D_C\}$ and hidden states $\{\mathbf{z}, \mathbf{C}\}$ has dynamics

$$\begin{aligned} dX_t &= -\kappa z_t (X_t - C_t) dt + \sqrt{2D} dW_t \\ dC_t &= \sqrt{2\big(D_C z_t + D_{est}(1 - z_t)\big)} dW_t^{(C)}. \end{aligned} \tag{1}$$

Where (as in the main text) $X_i = (X_i^{(1)}, X_i^{(2)})$ and $C_i = (C_i^{(1)}, C_i^{(2)})$ are 2D vectors. During confinement X_t has Ornstein-Uhlenbeck dynamics with centre C_t. This model assumes that switching can only occur at sampling points and that measurement noise is negligible. Measurement noise can be incorporated by introducing a model for the measured position Y_t dependent on the true position X_t, e.g. a Gaussian variate. This would allow complex measurement noise, such as raster scan anisotropic measurement errors, to be incorporated. However, the inference algorithm would be substantially more complex because of the additional hidden Markov chain X_t, so is not pursued here.

To integrate over a time step Δt when confined ($z_t = 1$), we can use the OU solution in X_t (assuming C_t is constant over a time step). Thus, ignoring time dependence of C_t over Δt we get,

$$X_{t+\Delta t}\Big|_{X_t, z_t = 1} \sim N\left(C_t + (X_t - C_t)e^{-\kappa \Delta t}, \frac{D}{\kappa}\left(1 - e^{-2\kappa \Delta t}\right)\right) \tag{2}$$

and $C_{t+\Delta t} \sim N(C_t, 2D_C \Delta t)$, where $N(x; \mu, \sigma)$ is the normal PDF with mean μ and variance σ, (we use this parameterisation and notation throughout). If $\Delta t \to 0$ we obtain the simplification $X_{t+\Delta t}\Big|_{X_t, z_t=1} \sim N\left(X_t - \kappa \Delta t (X_t - C_t), 2D\Delta t\right)$ as expected under Euler-Maruyama integration of the SDE. The latter is valid provided $\kappa \Delta t << 1$, or $D\Delta t << D/\kappa$, i.e. the diffusional displacement over Δt is substantially smaller than the confinement width of the Harmonic potential well.

Using this solution (Equation 2) we can state the following: for a 2D trajectory $\mathbf{X} = \{X_i, t_i\}_{i=1}^{N+1}$ the probability of observing $\mathbf{X}$ given the parameters (θ) and hidden states ($\mathbf{z}$, $\mathbf{C}$) is (recall that $\Delta X_i = X_{i+1} - X_i, \Delta t_i = t_{i+1} - t_i$)

$$\begin{aligned} \pi(\mathbf{X}|\theta, \mathbf{z}, \mathbf{C}) &= \prod_{i=1|z_i=0}^{N} N(\Delta X_i; 0, 2D\Delta t_i) \prod_{i=1|z_i=1}^{N} N\left(X_{i+1}; C_i + (X_i - C_i)e^{-\kappa \Delta t_i}, \frac{D}{\kappa}\left(1 - e^{-2\kappa \Delta t_i}\right)\right) \\ &= \prod_{i=1}^{N} N\left(\Delta X_i; z_i\left((C_i - X_i)\left(1 - e^{-\kappa \Delta t_i}\right)\right), D\left((1 - z_i)2\Delta t_i + \frac{z_i}{\kappa}\left(1 - e^{-2\kappa \Delta t_i}\right)\right)\right). \end{aligned} \tag{3}$$

Since ΔX_i is 2D this can be rewritten as a product over each coordinate. Using Bayes' rule we can write the posterior ($\Delta C_i = C_{i+1} - C_i$)

$$\begin{aligned}\pi(\theta, \mathbf{z}, \mathbf{C}|\mathbf{X}) \propto \pi(\theta, z_1, C_1) \prod_{i=1}^{N-1} \text{Bernoulli}(z_{i+1}; z_i(1-p_{esc}) + (1-z_i)p_{trap}) \\ \times \prod_{i=1}^{N} N\left(\Delta X_i; z_i\left(C_i - X_i + (X_i - C_i)e^{-\kappa \Delta t_i}\right), D\left((1-z_i)2\Delta t_i + \frac{z_i}{\kappa}\left(1 - e^{-2\kappa \Delta t_i}\right)\right)\right) \\ \times \prod_{i=1}^{N-1} N\left(\Delta C_i; 0, 2\Delta t_i (D_C z_i + D_{est}(1-z_i))\right), \end{aligned} \tag{4}$$

where $\pi(\theta, z_1, C_1)$ is the prior. We use conjugate priors for all parameters, specifically

$$\begin{aligned}\pi(\theta, z_1, C_1) =& \text{Unif}(D; D_{min}, D_{max})\text{Unif}(D_C; D_{C_{min}}, D_{max}/D_{ratio})\text{Unif}(\kappa; \kappa_{min}, \kappa_{max}) \\ &\times \text{Beta}(p_{esc}; a_{esc}, b_{esc})\text{Beta}(p_{trap}; a_{trap}, b_{trap}) \\ &\times \text{Bernoulli}(z_1; \pi_{trap}) N(C_1; \mu_{C_1}, 1/\tau_{C_1}).\end{aligned} \tag{5}$$

We choose prior parameters which enforce model conditions, for example a large D_{ratio} implies $D >> D_C$.

1.1 MCMC sampler

We developed an MCMC sampler which draws samples from this distribution as follows.

Parameter updates A Gibbs sampler can be used (under conjugate priors) for conditional posterior sampling of the diffusion coefficients (D and D_C) and transition probabilities (p_{esc}, p_{trap}). Specifically, the updates for D and D_C are inverse-Gamma

$$D^{-1}|_{D_C,\kappa,p_{esc},p_{trap},\mathbf{X},\mathbf{C},\mathbf{z}} \sim \text{Gamma}_T\left(N-1, \frac{1}{2}\sum_{i=1}^{N} \frac{\left(\Delta X_i - z_i\left(C_{t_i} - X_i + (X_{t_i} - C_{t_i})e^{-\kappa \Delta t_i}\right)\right)^2}{(1-z_i)2\Delta t_i + \frac{z_i}{\kappa}\left(1 - e^{-2\kappa \Delta t_i}\right)}, D_{min}, D_{max}\right) \tag{6}$$

$$D_C^{-1}|_{D,\kappa,p_{esc},p_{trap},\mathbf{X},\mathbf{C},\mathbf{z}} \sim \text{Gamma}_T\left(1 + \sum_{i|z_i=1} 1, \frac{1}{4}\sum_{i|z_i=1} \frac{\Delta C_i^2}{\Delta t_i}, D_{C_{min}}, D_{max}/D_{ratio}.\right) \tag{7}$$

where $\text{Gamma}_T(\alpha, \beta, x_{min}, x_{max})$ denotes a truncated Gamma distribution with parameters α and β, truncated at x_{min} and x_{max}. We enforce the truncation by rejecting any moves which lie outside this region. If $\sum_{i|z_i=1} 1 = 0$ then the conditional for D_C reduces to the prior, so we update by sampling from $\text{Unif}(D_{C_{min}}, D_{max}/D_{ratio})$.

The updates for the transition probabilities are from Beta distributions

$$p_{esc}|_{D,D_C,\kappa,p_{trap},\mathbf{X},\mathbf{z},\mathbf{C}} \sim \text{Beta}\left(a_{esc} + n_{10}, b_{esc} + n_{11}\right) \tag{8}$$

$$p_{trap}|_{D,D_C,\kappa,p_{esc},\mathbf{X},\mathbf{z},\mathbf{C}} \sim \text{Beta}\left(a_{trap} + n_{01}, b_{trap} + n_{00}\right) \tag{9}$$

where n_{ml} is the number of transitions from state m to state l, i.e.

$$n_{ml} = \sum_{i|z_{i-1}=m, z_i=l} 1. \tag{10}$$

The harmonic potential well strength (κ) is updated with a Metropolis-Hastings step. The conditional distribution is

$$\pi(\kappa|D, D_C, p_{esc}, p_{trap}, \mathbf{X}, \mathbf{C}, \mathbf{z}) \propto \prod_{z_i=1} N\left(\Delta X_i; (C_{t_i} - X_{t_i})\left(1 - e^{-\kappa \Delta t_i}\right), \frac{D}{\kappa}\left(1 - e^{-2\kappa \Delta t_i}\right)\right) \tag{11}$$

We use a Metropolis-Hastings move with Gaussian proposal

$$\kappa' \sim N(\kappa, s_\kappa) \tag{12}$$

where s_κ was chosen empirically to give an acceptance rate of approximately 0.3. The acceptance probability is

$$\alpha(\kappa'|\kappa) = \frac{\pi(\kappa'|D, D_C, p_{esc}, p_{trap}, \mathbf{X}, \mathbf{C}, \mathbf{z})}{\pi(\kappa|D, D_C, p_{esc}, p_{trap}, \mathbf{X}, \mathbf{C}, \mathbf{z})}. \tag{13}$$

We enforce the prior, Unif$(\kappa; \kappa_{min}, \kappa_{max})$, by rejecting any values of κ lying outside this interval.

Gibbs update for the Harmonic Potential well centre. For the centre, $\mathbf{C}$, we update using a blocked Gibbs move. We report the update here without calculation; the full derivation is given in section "Full derivation of Gibbs move for harmonic well centre". Let $\mathbf{C}_{j,n}$ be a block of length n starting at j, i.e. $\mathbf{C}_{j,n} = \{C_i\}_{i=j}^{j+n}$. The update is

$$\mathbf{C}_{j,n} \sim N(\boldsymbol{\mu}_{C_{j,n}}, \boldsymbol{\Sigma}^{-1}_{C_{j,n}}) \tag{14}$$

where the $n \times n$ precision matrix is

$$\boldsymbol{\Sigma}^{-1}_{C_{j,n}} = \begin{pmatrix} \Sigma^{-1}_{j,j} & \Sigma^{-1}_{j,j+1} & & & & \\ \Sigma^{-1}_{j+1,j} & \Sigma^{-1}_{j+1,j+1} & \Sigma^{-1}_{j+1,j+2} & & & \\ & \Sigma^{-1}_{j+2,j+1} & \ddots & & & \\ & & & \ddots & & \\ & & & & \ddots & \Sigma^{-1}_{j+n-1,j+n} \\ & & & & \Sigma^{-1}_{j+n,j+n-1} & \Sigma^{-1}_{j+n,j+n} \end{pmatrix} \tag{15}$$

with elements

$$\Sigma^{-1}_{i,i-1} = \Sigma^{-1}_{i-1,i} = \frac{-1}{2\Delta t_i(D_{est}(1-z_i) + D_C z_i)} \tag{16}$$

$$\Sigma^{-1}_{i,i}\big|_{2\le i\le N-1} = \frac{z_i(1-e^{\kappa\Delta t_i})^2}{\frac{D}{\kappa}(1-e^{-2\kappa\Delta t_i})} + \frac{1}{2\Delta t_{i-1}(D_C z_{i-1} + D_{est}(1-z_{i-1}))} + \frac{1}{2\Delta t_i(D_C z_i + D_{est}(1-z_i))}. \tag{17}$$

If the block contains the first timepoint, i.e. $j = 1$, then the first element is

$$\Sigma^{-1}_{1,1} = \frac{z_1(1-e^{\kappa\Delta t_1})^2}{\frac{D}{\kappa}(1-e^{-2\kappa\Delta t_1})} + \tau_{C_1} + \frac{1}{2\Delta t_1(D_C z_1 + D_{est}(1-z_1))} \tag{18}$$

and if the block contains the last timepoint, i.e. $j + n = N$, then the last element is

$$\Sigma^{-1}_{N,N} = \frac{z_N(1-e^{\kappa\Delta t_N})^2}{\frac{D}{\kappa}(1-e^{-2\kappa\Delta t_N})} + \frac{1}{2\Delta t_{N-1}(D_C z_{N-1} + D_{est}(1-z_{N-1}))}. \tag{19}$$

The mean $\boldsymbol{\mu}_{C_{j,n}}$ is given by solving

$$\boldsymbol{\Sigma}^{-1}_{C_{j,n}}\boldsymbol{\mu}_{C_{j,n}} = \mathbf{b}_{j,n} \tag{20}$$

where $\mathbf{b}_{j,n} = \{b_i\}_{i=j}^{j+n}$ is an n-vector with elements

$$b_i\Big|_{j+1\le i\le j+n-1} = \frac{z_i(1-e^{-\kappa\Delta t_i})^2)}{\frac{D}{\kappa}(1-e^{-2\kappa\Delta t_i})}\frac{\Delta X_i + X_i(1-e^{-\kappa\Delta t_i})}{1-e^{-\kappa\Delta t_i}} \tag{21}$$

$$b_j\Big|_{j\ne 1} = \frac{C_{j-1}}{2\Delta t_{j-1}(D_C z_{j-1} + D_{est}(1-z_{j-1}))} + \frac{z_i(1-e^{-\kappa\Delta t_i})^2)}{\frac{D}{\kappa}(1-e^{-2\kappa\Delta t_i})}\frac{\Delta X_i + X_i(1-e^{-\kappa\Delta t_i})}{1-e^{-\kappa\Delta t_i}} \tag{22}$$

$$b_{j+n}\Big|_{j+n\ne N} = \frac{C_{j+n+1}}{2\Delta t_{j+n}(D_C z_{j+n} + D_{est}(1-z_{j+n}))} + \frac{z_i(1-e^{-\kappa\Delta t_i})^2)}{\frac{D}{\kappa}(1-e^{-2\kappa\Delta t_i})}\frac{\Delta X_i + X_i(1-e^{-\kappa\Delta t_i})}{1-e^{-\kappa\Delta t_i}}. \tag{23}$$

If the block contains the first timepoint then

$$b_1 = \tau_{C_1}\mu_{C_1} + \frac{z_i(1-e^{-\kappa\Delta t_i})^2)}{\frac{D}{\kappa}(1-e^{-2\kappa\Delta t_i})}\frac{\Delta X_i + X_i(1-e^{-\kappa\Delta t_i})}{1-e^{-\kappa\Delta t_i}} \tag{24}$$

and if the block contains the last timepoint,

$$b_N = \frac{z_i(1 - e^{-\kappa \Delta t_i})^2)}{\frac{D}{\kappa}(1 - e^{-2\kappa \Delta t_i})} \frac{\Delta X_i + X_i(1 - e^{-\kappa \Delta t_i})}{1 - e^{-\kappa \Delta t_i}}. \tag{25}$$

Since $\mathbf{\Sigma}^{-1}_{C_{j,n}}$ is tridiagonal, Equation (20) can be efficiently solved for $\boldsymbol{\mu}_{C_{j,n}}$, for example using the left matrix division function in Matlab (with $\mathbf{\Sigma}^{-1}_{C_{j,n}}$ encoded as a sparse matrix). Hence the update can be sampled from Equation (14).

Metropolis-Hastings move for $\mathbf{z}$ *and* $\mathbf{C}$. Since the hidden states in the model are highly correlated, we developed a joint update for $\mathbf{z}$ and $\mathbf{C}$, a blocked Metropolis-Hastings move. To simulate values from $\pi(\mathbf{z}, \mathbf{C}|\theta, \mathbf{X})$, we first propose new values of $\{\mathbf{z}, \mathbf{C}\}$ for a block of length n, let $\{\mathbf{z}_{j,n}, \mathbf{C}_{j,n}\} = \{z_i, C_i\}_{i=j}^{j+n}$ denote this block. We use a joint proposal distribution

$$q(\mathbf{z}'_{j,n}, \mathbf{C}'_{j,n}|\mathbf{z}_{j,n}) = q(\mathbf{z}'_{j,n}|\mathbf{z}_{j,n}) q(\mathbf{C}'_{j,n}|\mathbf{z}'_{j,n}). \tag{26}$$

For $\mathbf{z}_{j,n}$ we use a proposal distribution $q(\mathbf{z}'_{j,n}|\mathbf{z}_{j,n})$ which is weighted towards complete confinement or free diffusion. Specifically, let 0^n and 1^n denote a sequence of 0's or 1's of length n, we propose $\mathbf{z}'_{j,n} = 0^n$ and $\mathbf{z}'_{j,n} = 1^n$ both with probability 1/3. Otherwise, with probability 1/3, we propose a random sequence generated from by the Markov chain with parameters p_{esc}, p_{trap}. The proposal density is hence

$$q(\mathbf{z}'_{j,n}|\mathbf{z}_{j,n})\Big|_{j\neq 1} = \begin{cases} \frac{1}{3} + \frac{1}{3}\prod_{i=j}^{n+j+1} \text{Bernoulli}\left(z_i; z_{i-1}(1-p_{esc}) + (1-z_{i-1})p_{trap}\right) & \text{if } \mathbf{z}'_{j,n} = 0^n \\ \frac{1}{3} + \frac{1}{3}\prod_{i=j}^{n+j+1} \text{Bernoulli}\left(z_i; z_{i-1}(1-p_{esc}) + (1-z_{i-1})p_{trap}\right) & \text{if } \mathbf{z}'_{j,n} = 1^n \\ \frac{1}{3}\prod_{i=j}^{n+j+1} \text{Bernoulli}\left(z_i; z_{i-1}(1-p_{esc}) + (1-z_{i-1})p_{trap}\right) & \text{any other } \mathbf{z}'_{j,n}. \end{cases} \tag{27}$$

If $j = 1$, then the first term in the product in Equation (27) is undefined, so we propose z_1 from the prior distribution, Bernoulli$(z_1; p_{trap}/(p_{trap} + p_{esc}))$, giving

$$q(\mathbf{z}'_{1,n}|\mathbf{z}_{1,n}) = \begin{cases} \frac{1}{3} + \frac{1}{3}\left(1 - \frac{p_{trap}}{p_{trap} + p_{esc}}\right) \\ \times \prod_{i=2}^{n+2} \text{Bernoulli}\left(z_i; z_{i-1}(1-p_{esc}) + (1-z_{i-1})p_{trap}\right) & \text{if } \mathbf{z}'_{1,n} = 0^n \\ \frac{1}{3} + \frac{1}{3}\frac{p_{trap}}{p_{trap} + p_{esc}} \\ \times \prod_{i=2}^{n+2} \text{Bernoulli}\left(z_i; z_{i-1}(1-p_{esc}) + (1-z_{i-1})p_{trap}\right) & \text{if } \mathbf{z}'_{1,n} = 1^n \\ \frac{1}{3}\text{Bernoulli}\left(z_1; \frac{p_{trap}}{p_{trap} + p_{esc}}\right) \\ \times \prod_{i=2}^{n+2} \text{Bernoulli}\left(z_i; z_{i-1}(1-p_{esc}) + (1-z_{i-1})p_{trap}\right) & \text{any other } \mathbf{z}'_{1,n}. \end{cases} \tag{28}$$

The right hand terms in the top two cases of Equations (27) and (28) are required because the sequences 0^n and 1^n are possible when simulating a Markov chain with parameters p_{esc} and p_{trap}.

We next propose a value for $\mathbf{C}_{j,n}$ using the block Gibbs update density derived earlier (Equations (14)-(25))

$$q(\mathbf{C}'_{j,n}|\mathbf{z}'_{j,n}) = N(\mathbf{C}'_{j,n}; \boldsymbol{\mu}_{C_{j,n}}, \mathbf{\Sigma}^{-1}_{C_{j,n}}) \tag{29}$$

where $\boldsymbol{\mu}_{C_{j,n}}$, $\boldsymbol{\Sigma}_{C_{j,n}}^{-1}$ are calculated (using Equations (15)-(20)) with the proposed value $\mathbf{z}'_{j,n}$. Up to proportionality, the density of interest is

$$\pi(\mathbf{z},\mathbf{C}|\theta,\mathbf{X}) \propto \prod_{i=1}^{N} N\left(\Delta X_i; z_i\left(C_i - X_i\right)\left(1-e^{-\kappa\Delta t_i}\right), D\left((1-z_i)2\Delta t_i + \frac{z_i}{\kappa}\left(1-e^{-2\kappa\Delta t_i}\right)\right)\right)$$

$$\times N(C_1, \mu_{C_1}, 1/\tau_{C_1}) \prod_{i=1}^{N-1} N\left(\Delta C_i; 0, 2\Delta t_i(D_C z_i + D_{est}(1-z_i))\right) \tag{30}$$

$$\times \text{Bernoulli}\left(z_1; \frac{p_{trap}}{p_{trap}+p_{esc}}\right) \prod_{i=1}^{N-1} \text{Bernoulli}(z_{i+1}; z_i(1-p_{esc}) + (1-z_i)p_{trap}). \tag{31}$$

which we call $P(\mathbf{z},\mathbf{C})$. The acceptance probability is thus

$$\alpha(\mathbf{z}',\mathbf{C}'|\mathbf{z},\mathbf{C}) = \min\left\{1, \frac{P(\mathbf{z}',\mathbf{C}')q(\mathbf{z}_{j,n}|\mathbf{z}'_{j,n})q(\mathbf{C}_{j,n}|\mathbf{z}_{j,n})}{P(\mathbf{z},\mathbf{C})q(\mathbf{z}'_{j,n}|\mathbf{z}_{j,n})q(\mathbf{C}'_{j,n}|\mathbf{z}'_{j,n})}\right\}. \tag{32}$$

By sequentially calculating these MCMC updates, we can sample from the posterior distribution $\pi(\theta,\mathbf{z},\mathbf{C}|\mathbf{X})$. Algorithm 1 (in Supporting Material) details this HPW model MCMC algorithm in pseudocode.

Blocking choice. The two blocked moves require a choice of block size n and starting point j. For the blocked Gibbs move for $\mathbf{C}$ we found that updating the whole Markov chain at once, i.e. $j = 1$ and $n = N-1$ was most efficient. For the blocked Metropolis-Hastings move for $\{\mathbf{z},\mathbf{C}\}$ we sample the block size n from Unif(B_{min}, B_{max}), and then sample j from Unif$(1, N-n)$. For all MCMC runs on real data we set $B_{min} = 2$ and $B_{max} = 200$. For MCMC runs on simulated data we heuristically tuned.

Algorithm runtime and convergence. The main factors which affect the algorithm runtime are the trajectory length and hidden state update blocking choices, detailed above. The main bottlenecks in the blocked hidden state updates are the calculation of $\mu_{C_{j,n}}$ and sampling from Equation (29). These require solving Equation (20) and Cholsky decomposition of $\Sigma_{C_{j,n}}^{-1}$ (note that inversion of $\Sigma_{C_{j,n}}^{-1}$ is not necessary) respectively. Clearly, the larger the blocks, the longer the runtime, although we were able to obtain speed ups by using sparse matrices in Matlab. However, runtime is not the same as MCMC algorithm convergence - which is affected by many factors and will vary depending on the data; the complexity of the confinement state sequence strongly affects the convergence rate as the algorithm needs to “find” the correct confinement sequence through hidden state updates. We advise trying different hidden state update blocking choices on new data sets to determine acceptable chain mixing and runtimes.

Algorithm 1 Metropolis-within-Gibbs sampler for harmonic potential well model

$K \leftarrow$ number of MCMC steps
$D_{min}, D_{C_{min}}, D_{max}, D_{ratio}, \kappa_{min}, \kappa_{max}, a_{esc}, b_{esc}, a_{trap}, b_{trap} \leftarrow$ prior parameters
$B_{max}, B_{min} \leftarrow$ maximum and minimum Metropolis-Hastings block sizes
$D_{est} \leftarrow$ estimated value for D (Equation (34))
Choose initial values - $D^{(0)}, D_C^{(0)}, \kappa^{(0)}, p_{esc}^{(0)}, p_{trap}^{(0)}, z^{(0)}$ (e.g. sample from Equation (5))
Calculate $\boldsymbol{\mu}_{C_{1,N-1}}$ (mean vector) and $\boldsymbol{\Sigma}^{-1}_{C_{1,N-1}}$ (precision matrix) using Equations (15)-(20)
$\mathbf{C}^{(0)} \leftarrow$ random number drawn from $N(\boldsymbol{\mu}_{C_{1,N-1}}, \boldsymbol{\Sigma}^{-1}_{C_{1,N-1}})$
for $k = 1$ to $k = K$ **do**
  $1/D^{(k)} \leftarrow$ random number drawn from $\Gamma\left(N-1, \frac{1}{2}\sum_{i=1}^{N} \frac{(\Delta X_i - z_i(C_{t_i} - X_i + (X_{t_i} - C_{t_i})e^{-\kappa \Delta t_i})^2}{(1-z_i)2\Delta t_i + \frac{z_i}{\kappa}(1-e^{-2\kappa\Delta t_i})}\right)$
  if $D < D_{min}$ **or** $D > D_{max}$ **then**
    $D^{(k)} \leftarrow D^{(k-1)}$
  end if
  if $\sum_{i|z_i=1} 1 = 0$ **then**
    $1/D_C^{(k)} \leftarrow$ random number drawn from Unif$(D_{C_{min}}, D_{max}/D_{ratio})$
  else
    $1/D_C^{(k)} \leftarrow$ random number drawn from $\Gamma\left(1 + \sum_{i|z_i=1} 1, \frac{1}{4}\sum_{i|z_i=1} \frac{\Delta C_i^2}{\Delta t_i}\right)$
  end if
  if $D_C < D_{C_{min}}$ **or** $D_C > D_{max}/D_{ratio}$ **then**
    $D_C^{(k)} \leftarrow D_C^{(k-1)}$
  end if
  $\kappa' \leftarrow$ random number drawn from $N(\kappa^{(k-1)}, s_\kappa)$
  if $\kappa' > \kappa_{min}$ **and** $\kappa' < \kappa_{max}$ **then**
    Calculate $\alpha(\kappa'|\kappa)$ using Equation (13)
    $u \leftarrow$ random number drawn from Unif$(0, 1)$
    if $u < \alpha(\kappa'|\kappa)$ **then**
      $\kappa^{(k)} \leftarrow \kappa'$
    end if
  end if
  Calculate $n_{00}, n_{01}, n_{10}, n_{11}$ using Equation (10)
  $p_{esc}^{(k)} \leftarrow$ random number drawn from Beta$(a_{esc} + n_{10}, b_{esc} + n_{11})$
  $p_{trap}^{(k)} \leftarrow$ random number drawn from Beta$(a_{trap} + n_{01}, b_{trap} + n_{00})$
  $n \leftarrow$ random integer drawn from $\{B_{min}, B_{min}+1, ..., B_{max}\}$
  $j \leftarrow$ random integer drawn from $\{1, ..., N-n\}$
  Calculate $\boldsymbol{\mu}_{C_{j,n}}$ (mean vector) and $\boldsymbol{\Sigma}^{-1}_{C_{j,n}}$ (precision matrix) using Equations (15)-(20)
  $\mathbf{C} \leftarrow$ random number drawn from $N(\boldsymbol{\mu}_{C_{1,N-1}}, \boldsymbol{\Sigma}^{-1}_{C_{1,N-1}})$
  $n \leftarrow$ random integer drawn from $\{B_{min}, B_{min}+1, ..., B_{max}\}$
  $j \leftarrow$ random integer drawn from $\{1, ..., N-n\}$
  $\mathbf{z}' \leftarrow$ proposed value drawn from $q(\mathbf{z})$ (Equation (27))
  Calculate $\boldsymbol{\mu}_{C_{j,n}}$ and $\boldsymbol{\Sigma}^{-1}_{C_{j,n}}$ (given $\mathbf{z}'$) using Equations (15)-(20)
  $\mathbf{C}'_{j,n} \leftarrow$ random number drawn from $N(\boldsymbol{\mu}_{C_{j,n}}, \boldsymbol{\Sigma}^{-1}_{C_{j,n}})$
  calculate $\alpha(\mathbf{z}', \mathbf{C}'|\mathbf{z}, \mathbf{C})$ using Equation (32)
  $u \leftarrow$ random number drawn from Unif$(0, 1)$
  if $u < \alpha(\mathbf{z}', \mathbf{C}'|\mathbf{z}, \mathbf{C})$ **then**
    $\mathbf{z} \leftarrow \mathbf{z}'$
    $\mathbf{C} \leftarrow \mathbf{C}'$
  end if
  $\mathbf{z}^{(k)} \leftarrow \mathbf{z}$
  $\mathbf{C}^{(k)} \leftarrow \mathbf{C}$
end for

Initial values and priors. For all MCMC runs on simulated and experimental data the priors were as follows

$$\begin{aligned}\pi(D) &= \text{Unif}(D; 0, 4\,\mu\text{m}^2\,\text{s}^{-1})\\ \pi(D_C) &= \text{Unif}(D_C; 0, 0.04\,\mu\text{m}^2\,\text{s}^{-1})\\ \pi(\kappa) &= \text{Unif}(\kappa; 0\,\text{s}^{-1}, 20\,000\,\text{s}^{-1})\\ \pi(p_{esc}) &= \text{Beta}(p_{esc}; 1, 1000)\\ \pi(p_{trap}) &= \text{Beta}(p_{trap}; 1, 1000).\end{aligned} \tag{33}$$

We chose non-zero $D_{C_{min}}$ because very low D_C values occasionally caused computational overflow in the blocked update covariance matrix $\mathbf{\Sigma}_{C_{k,n}}$. We chose non-zero κ_{min} because MCMC chains were occasionally very slow to converge from very low values of κ. Inferred posterior distributions for both these parameters were much higher than these minimum values. We chose an informative prior, $\text{Beta}(1, 1000)$, on the transition probabilities to inhibit rapid switching and short confinement events. These events are also inhibited by the implicit prior penalty against a transition to a confinement state, due to the diffusion of the confinement centre C_t when the particle X_t is not confined. The probability of C_t and X_t being in close proximity is proportional to $\frac{1}{(D_{est}+D)t}$, where t is the time since the end of the last confinement event. This prior dependence is weak and no issues were detected due to it, see Fig. S5. We initialised D, D_C, κ, p_{esc} and p_{trap} by sampling from the prior distributions. For D_{est} (the diffusion coefficient of C when X is confined) we used the Brownian motion maximum likelihood estimate

$$D_{est} = \frac{1}{4N}\sum_{i=1}^{N}\frac{\Delta X_i^2}{\Delta t_i}. \tag{34}$$

For $\mathbf{z}$ we initialised by simulating a Markov chain using the initial p_{esc} and p_{trap}. For $\mathbf{C}$ we initialised by a Gibbs block update given $\mathbf{z}$.

Convergence diagnostics. For the parameters, we ran multiple chains and assessed convergence using the Gelman point scale reduction factor (PSRF) (1). On real data we considered an MCMC run converged if the PSRF was less than 1.2 for all variables. We initialised the Markov chains by sampling from the prior distributions. Under the given prior parameters the initial values were over dispersed with respect to the target distributions on all trajectories (a requirement of the PSRF method).

Full derivation of Gibbs move for harmonic well centre

Here we calculate a Gibbs update for the harmonic well centre ($\mathbf{C}$). As described earlier in Note S1, we update in blocks of length n, $\mathbf{C}_{j,n} = \{C_i\}_{i=j}^{j+n}$, where $1 \le j \le N-n$. We derive an update by comparing the conditional distribution for a block (obtained from the posterior distribution, Equation (30) in the main text), and the multivariate normal PDF. On the one hand we have the conditional distribution for $\mathbf{C}_{j,n}$, for which there are two cases

$$\pi(\mathbf{C}_{j,n}|\mathbf{C}_{-j,n},\mathbf{z},\theta,\mathbf{X})\Big|_{j\neq 1} \propto \exp\left(\sum_{i=j}^{j+n}\frac{-\left(\Delta X_i - z_i(C_i - X_i + (X_i - C_i)e^{-\kappa\Delta t_i})\right)^2}{2D\left((1-z_i)2\Delta t_i + \frac{z_i}{\kappa}(1-e^{-2\kappa\Delta t_i})\right)} + \sum_{i=j}^{j+n+1}\frac{-\Delta C_i^2}{4\Delta t_i(D_C z_i + D_{est}(1-z_i))}\right) \tag{35}$$

$$\begin{aligned}\pi(\mathbf{C}_{j,n}|\mathbf{C}_{-j,n},\mathbf{z},\theta,\mathbf{X})\Big|_{j=1} \propto \exp\Bigg(&\frac{-\tau_{C_1}}{2}(C_1-\mu_{C_1})^2 + \sum_{i=j}^{j+n}\frac{-\left(\Delta X_i - z_i(C_i - X_i + (X_i - C_i)e^{-\kappa\Delta t_i})\right)^2}{2D\left((1-z_i)2\Delta t_i + \frac{z_i}{\kappa}(1-e^{-2\kappa\Delta t_i})\right)}\\ &+ \sum_{i=j}^{j+n+1}\frac{-\Delta C_i^2}{4\Delta t_i(D_C z_i + D_{est}(1-z_i))}\Bigg)\end{aligned} \tag{36}$$

where $\mathbf{C}_{-j,n} = \mathbf{C} \setminus \mathbf{C}_{j,n}$. On the other hand, the multivariate normal PDF with mean $\boldsymbol{\mu}_{C_{j,n}}$ and precision matrix $\boldsymbol{\Sigma}^{-1}_{C_{j,n}}$, up to proportionality with respect to $\mathbf{C}_{j,n}$, is

$$\begin{aligned}\exp\Bigg[-\frac{1}{2}\Big((\mathbf{C}_{j,n}-\boldsymbol{\mu}_{C_{j,n}})^T\boldsymbol{\Sigma}^{-1}_{C_{j,n}}(\mathbf{C}_{j,n}-\boldsymbol{\mu}_{C_{j,n}})\Big)\Bigg] &= \exp\Bigg[-\frac{1}{2}\sum_{l=j}^{j+n}\sum_{m=j}^{j+n}(C_l-\mu_l)(C_m-\mu_m)\Sigma^{-1}_{l,m}\Bigg] \\ &= \exp\Bigg[-\frac{1}{2}\sum_{l=j}^{j+n}\sum_{m=j}^{j+n}(C_lC_m - C_l\mu_m - C_m\mu_l + \mu_l\mu_m)\Sigma^{-1}_{l,m}\Bigg]\end{aligned} \tag{37}$$

where $\Sigma^{-1}_{l,m}$ denotes the (l,m)th element of the precision matrix and μ_l denotes the lth element of the mean vector. Thus we can calculate a multivariate normal update by comparing the coefficients in the exponential for Equations (35) and (37) (also using Equation (36) if $j=1$). For the squared and cross terms this gives

$$C_i^2\Big|_{2\le i\le N-1} : -\frac{1}{4\Delta t_{i-1}(D_C z_{i-1} + D_{est}(1-z_{i-1}))} - \frac{z_i(1-e^{\kappa\Delta t_i})^2}{2\frac{D}{\kappa}(1-e^{-2\kappa\Delta t_i})} - \frac{1}{4\Delta t_i(D_C z_i + D_{est}(1-z_i))} = -\frac{1}{2}\Sigma^{-1}_{i,i} \tag{38}$$

$$C_iC_{i-1} : \frac{1}{2\Delta t_{i-1}(D_C z_{i-1} + D_{est}(1-z_{i-1}))} = -\Sigma^{-1}_{i,i-1} = -\Sigma^{-1}_{i-1,i}. \tag{39}$$

And for $i=1$ and $i=N$ we have

$$C_1^2 : -\frac{\tau_{C_1}}{2} - \frac{z_i(1-e^{\kappa\Delta t_i})^2}{2\frac{D}{\kappa}(1-e^{-2\kappa\Delta t_i})} - \frac{1}{4\Delta t_1(D_C z_1 + D_{est}(1-z_1))} = -\frac{1}{2}\Sigma^{-1}_{1,1} \tag{40}$$

$$C_N^2 : -\frac{1}{4\Delta t_{N-1}(D_C z_{N-1} + D_{est}(1-z_{N-1}))} - \frac{z_i(1-e^{\kappa\Delta t_i})^2}{2\frac{D}{\kappa}(1-e^{-2\kappa\Delta t_i})} = -\frac{1}{2}\Sigma^{-1}_{N,N}. \tag{41}$$

By solving Equations (38)-(41) we can hence write the n by n precision matrix

$$\boldsymbol{\Sigma}^{-1}_{C_{j,n}} = \begin{pmatrix} \Sigma^{-1}_{j,j} & \Sigma^{-1}_{j,j+1} & & & \\ \Sigma^{-1}_{j+1,j} & \Sigma^{-1}_{j+1,j+1} & \Sigma^{-1}_{j+1,j+2} & & \\ & \Sigma^{-1}_{j+2,j+1} & \ddots & & \\ & & & \ddots & \\ & & & \ddots & \Sigma^{-1}_{j+n-1,j+n} \\ & & & \Sigma^{-1}_{j+n,j+n-1} & \Sigma^{-1}_{j+n,j+n} \end{pmatrix}. \tag{42}$$

The mean vector $\boldsymbol{\mu}_{C_{j,n}}$ can be calculated by comparing the C_i coefficients. For $j+1 \le i \le j+n-1$ this gives

$$C_i\Big|_{j+1\le i\le j+n-1} : \frac{z_i(1-e^{-\kappa\Delta t_i})^2)}{\frac{D}{\kappa}(1-e^{-2\kappa\Delta t_i})}\frac{\Delta X_i + X_i(1-e^{-\kappa\Delta t_i})}{1-e^{-\kappa\Delta t_i}} = \sum_{m=j}^{j+n}\mu_m\Sigma^{-1}_{i,m}. \tag{43}$$

And providing the block does not contain the first or last timepoint, we have

$$C_j : \frac{C_{j-1}}{2\Delta t_{j-1}(D_C z_{j-1} + D_{est}(1-z_{j-1}))} + \frac{z_j(1-e^{-\kappa\Delta t_j})^2)}{\frac{D}{\kappa}(1-e^{-2\kappa\Delta t_j})}\frac{\Delta X_j + X_j(1-e^{-\kappa\Delta t_j})}{1-e^{-\kappa\Delta t_j}} = \sum_{m=j}^{j+n}\mu_m\Sigma^{-1}_{j,m} \tag{44}$$

$$C_{j+n} : \frac{C_{j+n+1}}{2\Delta t_{j+n}(D_C z_{j+n} + D_{est}(1-z_{j+n}))} + \frac{z_{j+n}(1-e^{-\kappa\Delta t_{j+n}})^2)}{\frac{D}{\kappa}(1-e^{-2\kappa\Delta t_{j+n}})}\frac{\Delta X_{j+n} + X_{j+n}(1-e^{-\kappa\Delta t_{j+n}})}{1-e^{-\kappa\Delta t_{j+n}}} = \sum_{m=j}^{j+n}\mu_m\Sigma^{-1}_{j+n,m}. \tag{45}$$

If the block contains the first timepoint, i.e. $j=1$ we have

$$C_1 : \tau_{C_1}\mu_{C_1} + \frac{z_1(1-e^{-\kappa\Delta t_1})^2)}{\frac{D}{\kappa}(1-e^{-2\kappa\Delta t_1})}\frac{\Delta X_1 + X_1(1-e^{-\kappa\Delta t_1})}{1-e^{-\kappa\Delta t_1}} = \sum_{m=j}^{j+n}\mu_m\Sigma^{-1}_{1,m} \tag{46}$$

and if the block contains the last timepoint, i.e. $j+n=N$, then

$$C_N : \frac{z_i(1-e^{-\kappa\Delta t_i})^2)}{\frac{D}{\kappa}(1-e^{-2\kappa\Delta t_i})} \frac{\Delta X_i + X_i(1-e^{-\kappa\Delta t_i})}{1-e^{-\kappa\Delta t_i}} = \sum_{m=j}^{j+n} \mu_m \Sigma^{-1}_{N,m}. \tag{47}$$

To calculate $\boldsymbol{\mu}_{C_{j,n}}$ we solve the system of linear equations

$$\boldsymbol{\Sigma}^{-1}_{C_{j,n}} \boldsymbol{\mu}_{C_{j,n}} = \mathbf{b}_{j,n} \tag{48}$$

where $\mathbf{b}_{j,n}$ is a column vector with elements

$$b_i = \left\{ \frac{z_i(1-e^{-\kappa\Delta t_i})^2)}{\frac{D}{\kappa}(1-e^{-2\kappa\Delta t_i})} \frac{\Delta X_i + X_i(1-e^{-\kappa\Delta t_i})}{1-e^{-\kappa\Delta t_i}} \right\}_{i=j+1}^{j+n-1} \tag{49}$$

and b_j, b_{j+n} from the left hand side of Equations (44) and (45). (Or Equation (46) or (47) if $j=1$ or $j+n=N$ respectively.) Since $\boldsymbol{\Sigma}^{-1}_{C_{j,n}}$ is tridiagonal this equation can be efficiently solved, for example using the left matrix division function (with $\boldsymbol{\Sigma}^{-1}_{C_{j,n}}$ as a sparse matrix) in Matlab. Given $\boldsymbol{\mu}_{C_{j,n}}$ and $\boldsymbol{\Sigma}^{-1}_{C_{j,n}}$ the Gibbs update is

$$\mathbf{C}_{j,n} \sim N(\boldsymbol{\mu}_{C_{j,n}}, \boldsymbol{\Sigma}^{-1}_{C_{j,n}}). \tag{50}$$

Pseudocode for harmonic potential confinement HMM simulation

In the simulations we include a drift term for the centre so that C tracks X when not confined; this ensures that when the particle switches from free diffusion to confinement the well centre C is close to X which is a realistic requirement. This allows for confinement zones to be small relative to the field of view. This tracking of X by C isn't included in the inference algorithm as diffusion alone is sufficient to allow the MC to find appropriate paths.

Algorithm 2 Simulation algorithm for harmonic potential well model.

$\{D, D_{est}, D_C, \kappa, p_{esc}, p_{trap}\} \leftarrow$ choice of model parameters
$\{\Delta t_i\}_{i=1}^{N+1} \leftarrow$ choice of time steps ($\Delta t_i = t_{i+1} - t_i$)
$X_1 \leftarrow$ initial particle position
$C_1 \leftarrow$ initial harmonic well centre position
$z_1 \leftarrow$ initial confinement state
for $i=1$ to $i=N-1$ **do**
  $z_{i+1} \leftarrow$ random number drawn from Bernoulli$(z_i(1-p_{esc})) + (1-z_i)p_{trap})$
  $C_{i+1} \leftarrow$ random number drawn from $N\left(C_i + \kappa\Delta t_i(1-z_i)(X_i - C_i), 2\Delta t_i(D_C z_i + D_{est}(1-z_i))\right)$
  $X_{i+1} \leftarrow$ random number drawn from $N\left(X_i + z_i(C_i - X_i + (X_i - C_i)e^{-\kappa\Delta t_i}), D\left((1-z_i)2\Delta t_i + \frac{z_i}{\kappa}(1-e^{-2\kappa\Delta t_i})\right)\right)$
end for
$X_{N+1} \leftarrow$ random number drawn from $N\Big(X_N + z_N(C_N - X_N + (X_N - C_N)e^{-\kappa\Delta t_N}),$
$D\left((1-z_N)2\Delta t_N + \frac{z_N}{\kappa}(1-e^{-2\kappa\Delta t_N})\right)\Big)$

2 Note S2: Data preprocessing

The initial analysis of this dataset (2) revealed superdiffusive behaviour at time delays less than 1×10^{-4} s, which was attributed to dynamic error in measurements due to sub-nanometre localisation precision. An analysis revealed that displacement angles showed a bias towards horizontal displacements (Fig. S1). We determined that this behaviour was a result of the raster scan whereby there was an inaccuracy in the x coordinate at these fast recording rates. This effect was statistically significant in a Chi-squared test (with the null hypothesis that angular displacements follow a uniform distribution), Fig. S2.

This bias was removed, in $40\,\mathrm{nm}$ AuNP/CTxB/GM1 trajectories on mica, by subsampling at rate 10, Fig. S2A. These trajectories were previously shown to display no trapping (2); hence the bias that remains even when subsampling at higher rates in displacement angles for $20\,\mathrm{nm}$ AuNP/CTxB/GM1 on glass trajectories is presumably due to directional displacements in trapping events, Fig. S2A. We also investigated the effect of window averaging, taking the average particle position over a window size n, and found similar trends to subsampling, Fig. S2B. This analysis therefore indicated that a subsampling rate of 10 is sufficient to remove these spatial localisation artifacts. This reduces the trajectory sampling rate from $50\,\mathrm{kHz}$ to $5\,\mathrm{kHz}$.

Additionally, a small number of $20\,\mathrm{nm}$ AuNP/CTxB/GM1 trajectories on glass (7 out of 71) had displacements that were unreasonably large, probably caused by an additional AuNP in the focal area. We dealt with this by visually inspecting the trajectories, and removing (before subsampling) the section of the trajectory with the artifacts. All presented analysis is on this subsampled, quality assured set.

Supporting References

1. Gelman, A., J. B. Carlin, H. S. Stern, D. B. Dunson, A. Vehtari, and D. B. Rubin, 2013. Bayesian Data Analysis, Third Edition. CRC Press.
2. Spillane, K. M., J. Ortega-Arroyo, G. de Wit, C. Eggeling, H. Ewers, M. I. Wallace, and P. Kukura, 2014. High-Speed Single-Particle Tracking of GM1 in Model Membranes Reveals Anomalous Diffusion due to Interleaflet Coupling and Molecular Pinning. *Nano Letters* 14:5390–5397.

1 Table S1: Correlations between confinement event statistics.

	Event lifetime	Mean confinement radius	Radial skewness
Mean confinement radius	0.01 ($p = 0.81$)		
Radial skewness	-0.13 ($p = 0.0003$)	-0.28 ($p < 0.0001$)	
Radial mean-median distance	-0.16 ($p < 0.0001$)	0.29 ($p < 0.0001$)	0.26 ($p < 0.0001$)
Radial SD	-0.09 ($p = 0.01$)	0.69 ($p < 0.0001$)	0.12 ($p = 0.0006$)

Pearson correlation coefficient between confinement statistics, for the set of all confinement events, except those which contained either the first or last timepoint. Exact calculations for confinement statistics are also given in Table 1, main text.

2 Table S2: Comparison of within and between trajectory variances for confinement event statistics.

Statistic	Mean within trajectory variance	Variance across all events	ANOVA p-value
Mean confinement radius ($\bar{R}_{lm}$)	$3.1 \times 10^{-5}\,\mu m^2$	$4.8 \times 10^{-5}\,\mu m^2$	1.2×10^{-6}
Radial skewness (S_{lm})	0.49	0.77	9.1×10^{-4}
Radial mean-median distance	$2.9 \times 10^{-7}\,\mu m^2$	$4.4 \times 10^{-7}\,\mu m^2$	0.06
Radial SD	$4.5 \times 10^{-6}\,\mu m^2$	$4.9 \times 10^{-6}\,\mu m^2$	3.0×10^{-30}
Confinement event lifetime	$0.025\,s^2$	$0.029\,s^2$	2.6×10^{-19}

Spatial statistics calculated on the set of 271 events obtained by applying the restrictions given in Table 1 in the main text. Lifetime statistics calculated on set of 214 events, also described in Table 1, main text. ANOVA calculated across all trajectories, not including events revisiting the same location.

1 Fig. S1: Radial histogram of angular displacements for a 40 nm AuNP/CTxB/GM1 trajectory on mica.

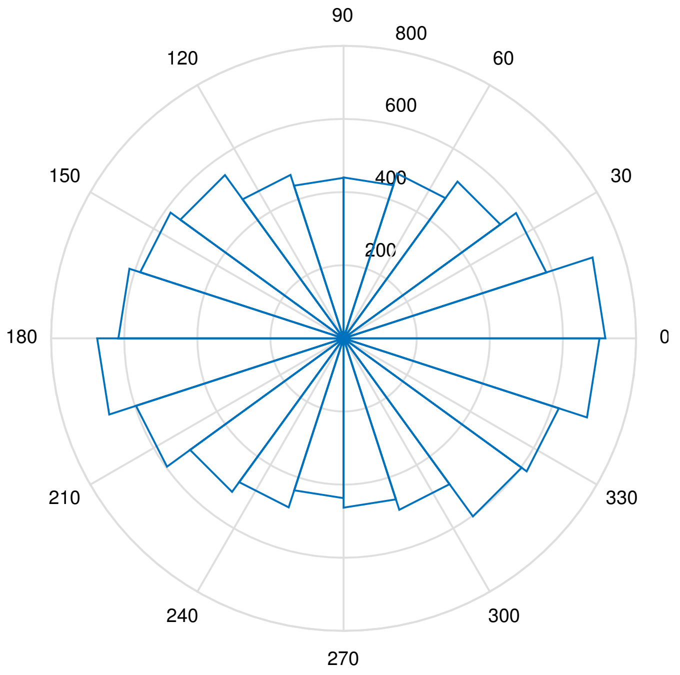

The angular displacement (plotted in degrees) in radians is $\phi_i = \arctan(\Delta X_{2i}/\Delta X_{1i}) + \pi$, where $\Delta X_i = \{\Delta X_{1i}, \Delta X_{2i}\}$.

2 Fig. S2: Subsampling removes the angular bias in trajectory displacements.

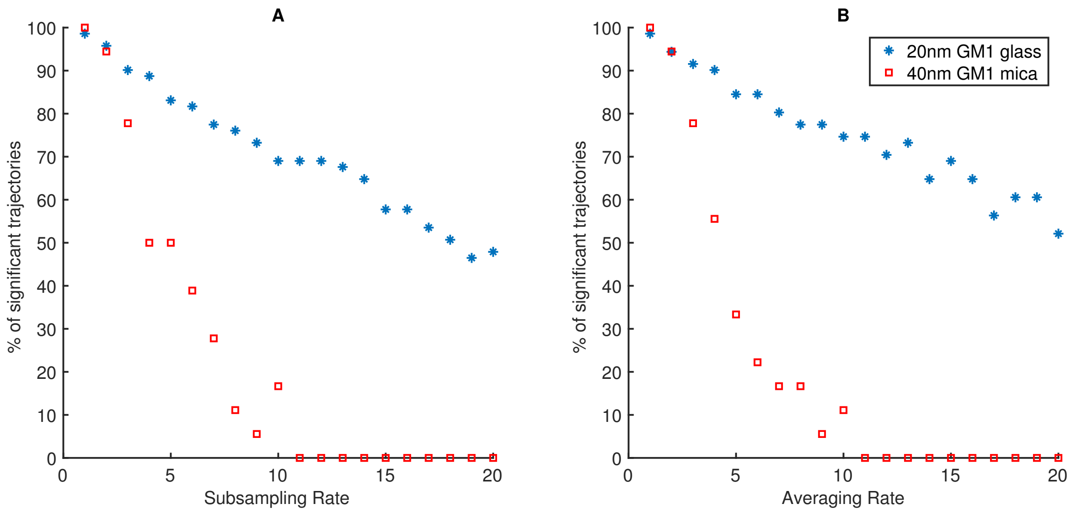

Percentage of GM1 trajectories where the null hypothesis (angular displacements follow a uniform distribution) was rejected in a Chi-square test, plotted against subsampling (A) or averaging rate (B). For each trajectory, the null hypothesis was rejected if $p < 0.0003$ (p=0.05 with Bonferroni correction, 169 trajectories in total).

3 Fig. S3: Confinement probabilities cluster around 0 and 1.

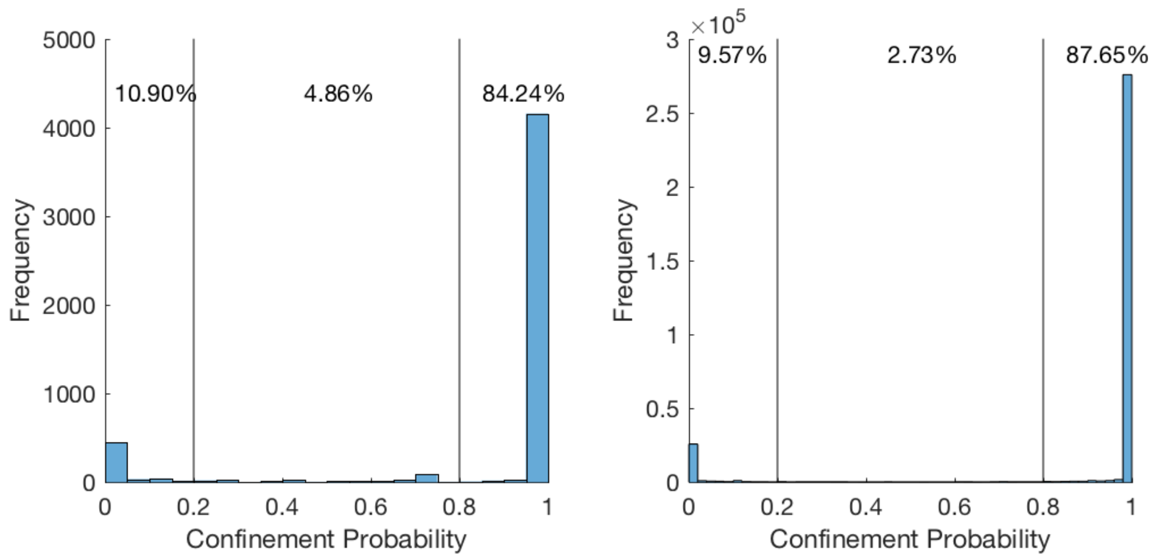

Histograms of inferred (posterior) probabilities for confinement state $\mathbf{z}$ on $20\,\mathrm{nm}$ AuNP/CTxB/GM1 trajectories. (A) Single representative trajectory. (B) Pooled probabilities across all trajectories (66). Percentages report the weight in each section (0-0.2, 0.2-0.8 and 0.8-1).

4 Fig S4: Fit of HPW model to a simulated trajectory

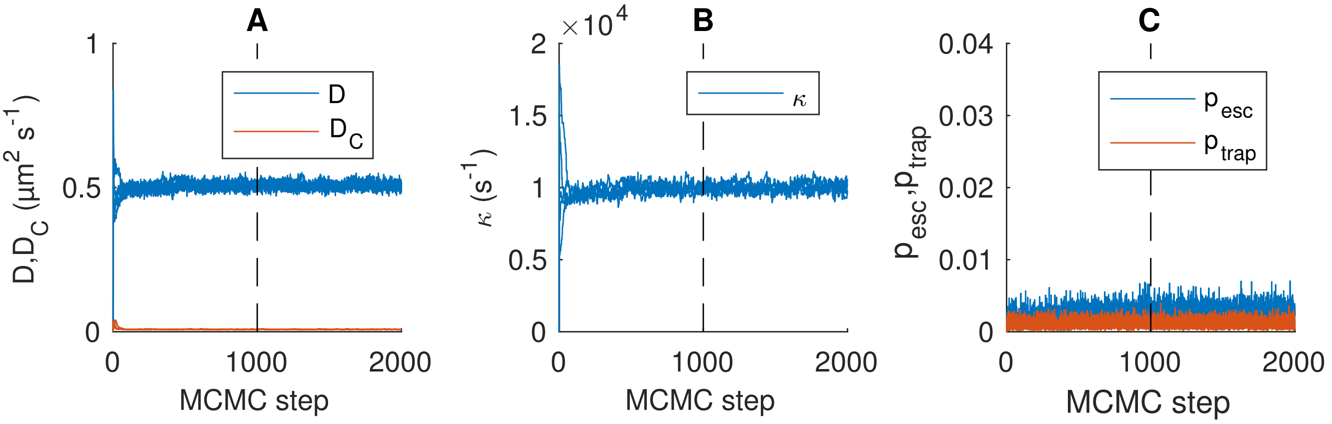

(A) MCMC chains for D (blue) and D_C (red). (B) MCMC chain for κ. (C) MCMC chains for p_{esc} and p_{trap}. For each parameter 12 independent MCMC runs are shown. Corresponding parameter posterior distributions are shown in Fig. 2 in the main text.

5 Fig. S5: Varying D_{est} in simulations: parameter estimates

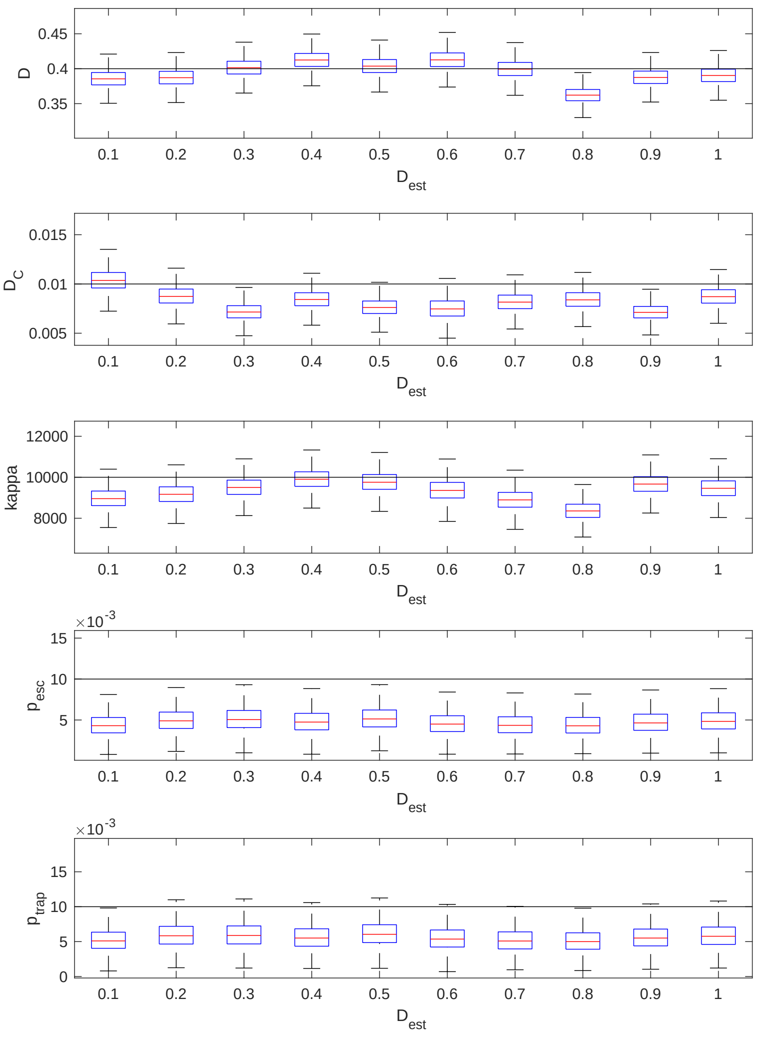

Parameter posterior samples for the harmonic potential well model applied to simulated trajectories with varying D_{est} values. Other simulation parameters: $D = 0.4\,\mu\text{m}^2\,\text{s}^{-1}$, $D_C = 0.01\,\mu\text{m}^2\,\text{s}^{-1}$, $\kappa = 10\,000\,\text{s}^{-1}$, $p_{esc} = 0.01$, $p_{trap} = 0.01$.

6 Fig. S6: Varying D_{est} in simulations with localisation error: parameter estimates

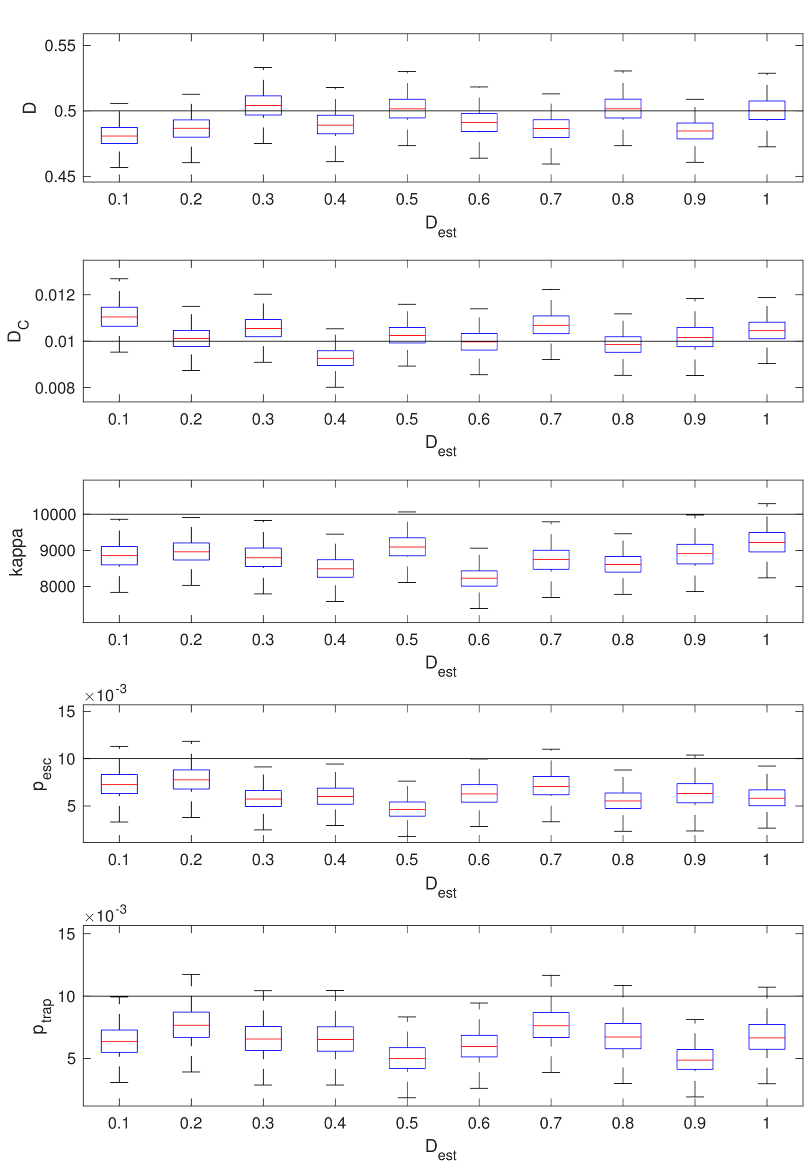

Parameter posterior samples for the harmonic potential well model applied to simulated trajectories with varying D_{est} values. Trajectories include static localisation (measurement) error with standard deviation $2.7\,\mathrm{nm}$, and were preprocessed by subsampling at rate 10. Other simulation parameters: $D = 0.5\,\mu\mathrm{m}^2\,\mathrm{s}^{-1}$, $D_C = 0.01\,\mu\mathrm{m}^2\,\mathrm{s}^{-1}$, $\kappa = 10\,000\,\mathrm{s}^{-1}$, $p_{esc} = 0.01$, $p_{trap} = 0.01$.

7 Fig. S7: Varying static localization error in simulations

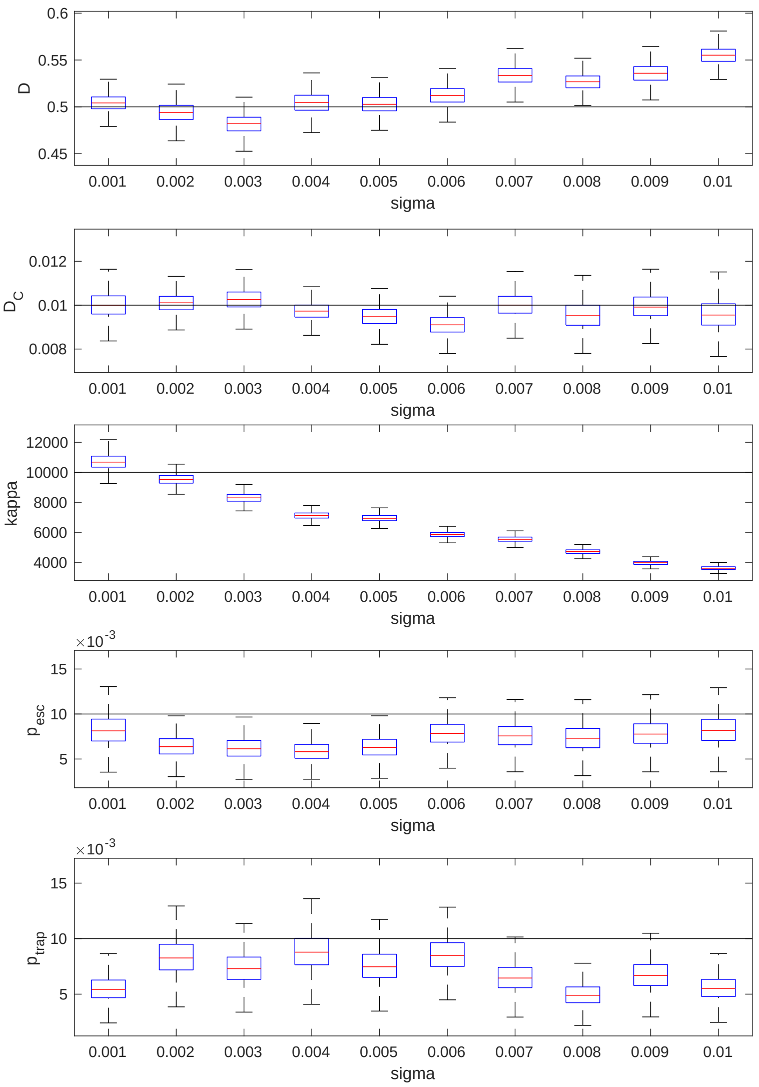

Parameter posterior distributions for harmonic potential well model applied to simulated trajectories including static localisation error with varying standard deviation σ (in microns). Trajectories were preprocessed by subsampling at rate 10. Black lines represent simulated parameter values ($D = 0.5\,\mu m^2\,s^{-1}$, $D_C = 0.01\,\mu m^2\,s^{-1}$, $\kappa = 10\,000\,s^{-1}$, $p_{esc} = 0.01$, $p_{trap} = 0.01$).

8 Fig. S8: Varying trajectory length in simulations

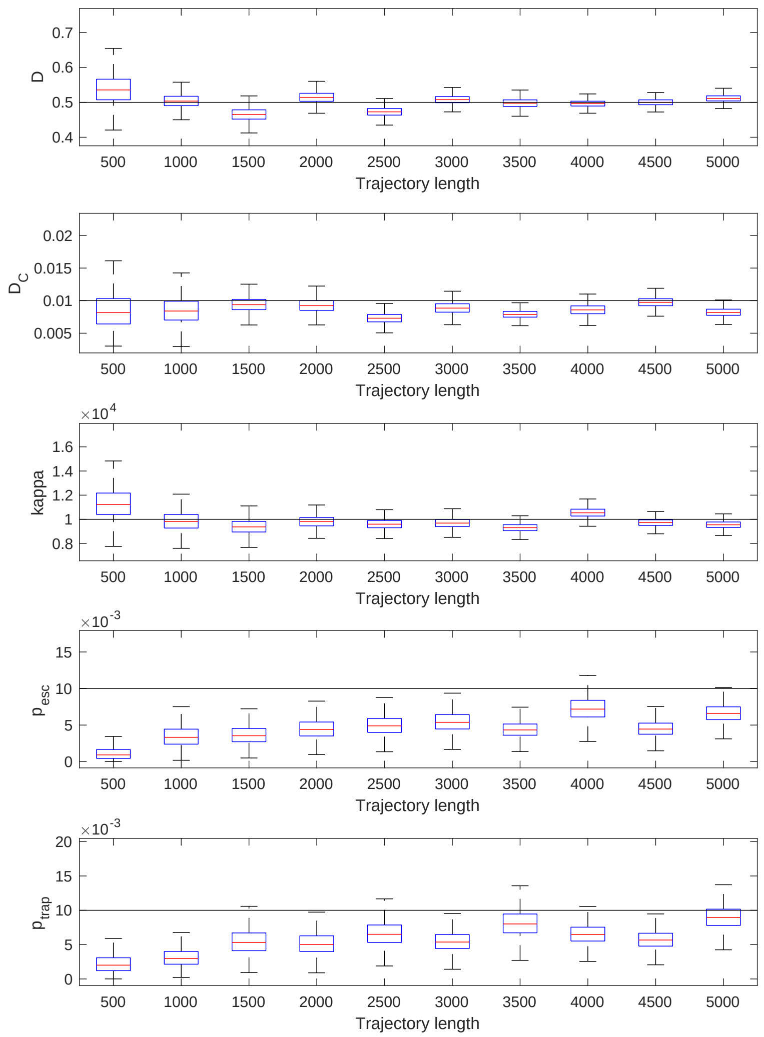

Parameter posterior samples for harmonic potential well model applied to simulated trajectories of varying length (between 500 and 5000 time steps). Black lines represent the simulated parameter values ($D = 0.5\,\mu\text{m}^2\,\text{s}^{-1}$, $D_C = 0.01\,\mu\text{m}^2\,\text{s}^{-1}$, $\kappa = 10\,000\,\text{s}^{-1}$, $p_{esc} = 0.01$, $p_{trap} = 0.01$).

9 Fig. S9: Varying number of confinement events in simulations

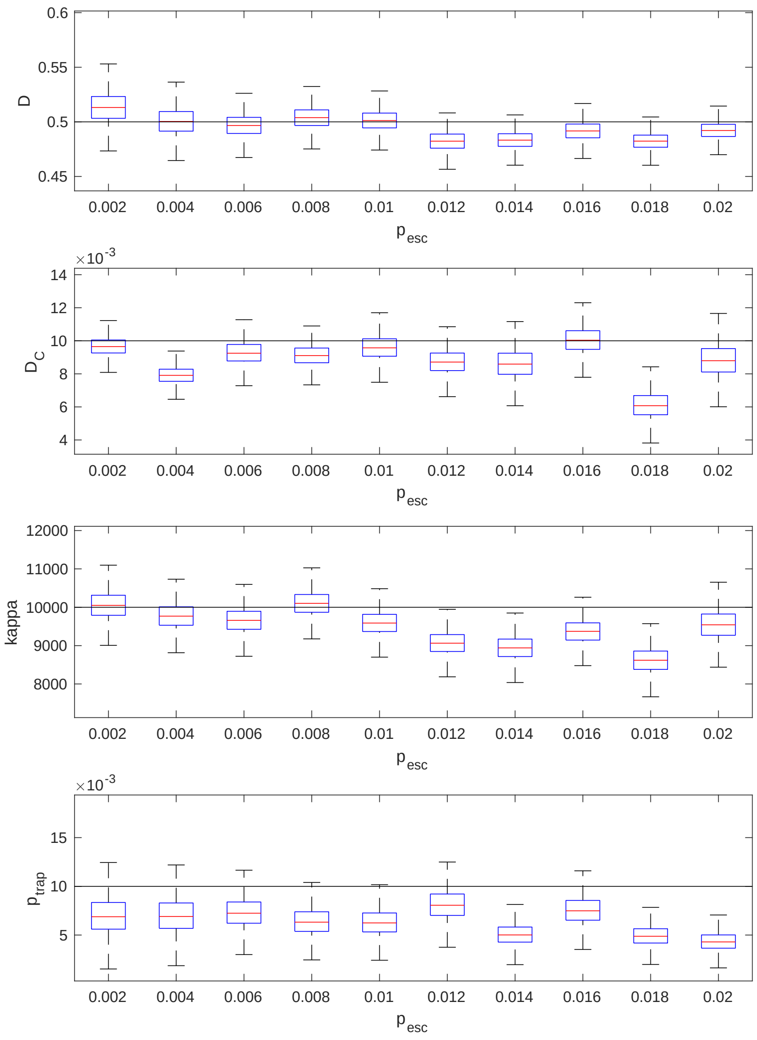

Parameter posterior distributions for harmonic potential well model applied to simulated trajectories with varying confinement event length. The event length was changed by adjusting the p_{esc} parameter between 0.002 and 0.02. Black lines represent simulated parameter values ($D = 0.5\,\mu\mathrm{m}^2\,\mathrm{s}^{-1}$, $D_C = 0.01\,\mu\mathrm{m}^2\,\mathrm{s}^{-1}$, $\kappa = 10\,000\,\mathrm{s}^{-1}$, $p_{esc} = 0.01$, $p_{trap} = 0.01$).

10 Fig. S10: Varying subsampling rate in simulations: parameter estimates

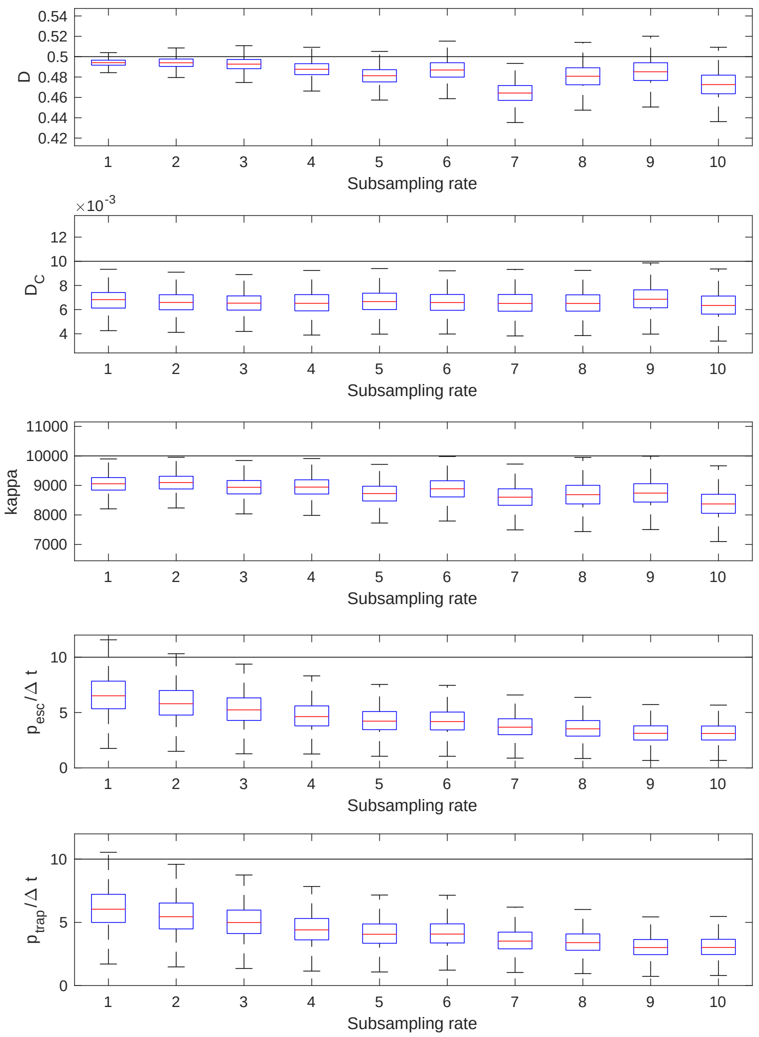

Parameter posterior samples for the harmonic potential well model applied to a single simulated trajectory (total length 20000 time steps) subsampled at different rates. We plot the rates, $p_{esc}/\Delta t$ and $p_{trap}/\Delta t$, instead of transition probabilities as these are invariant to the subsampling rate. Black lines represent simulated parameter values ($D = 0.5\,\mu\mathrm{m}^2\,\mathrm{s}^{-1}$, $D_C = 0.01\,\mu\mathrm{m}^2\,\mathrm{s}^{-1}$, $\kappa = 10\,000\,\mathrm{s}^{-1}$, $p_{esc}/\Delta t = 10\,\mathrm{s}^{-1}$, $p_{trap}/\Delta t = 10\,\mathrm{s}^{-1}$).

11 Fig. S11: Varying subsampling rate in simulations: confinement state

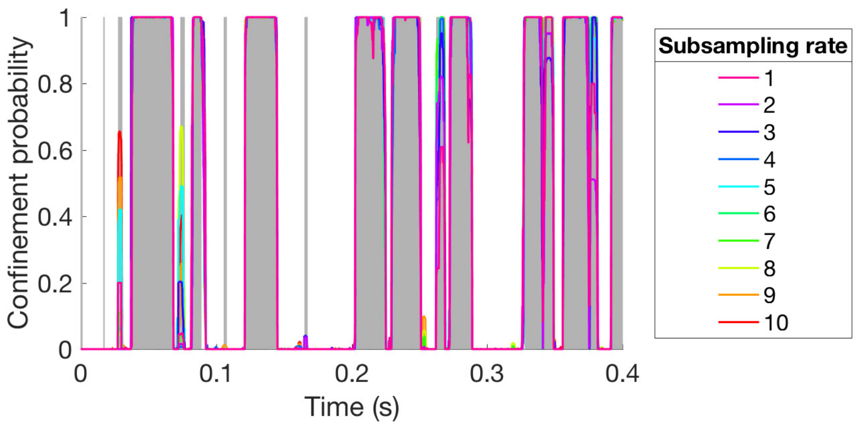

Confinement state posterior estimates for the model applied to a single simulated trajectory (total length 20000 time steps) subsampled at different rates. Grey area denotes the simulated (true) confinement state. Simulation parameters: $D = 0.5\,\mu\mathrm{m}^2\,\mathrm{s}^{-1}$, $D_C = 0.01\,\mu\mathrm{m}^2\,\mathrm{s}^{-1}$, $\kappa = 10\,000\,\mathrm{s}^{-1}$, $p_{esc} = 0.002$, $p_{trap} = 0.002$.

12 Fig. S12: Varying confinement strength in simulations

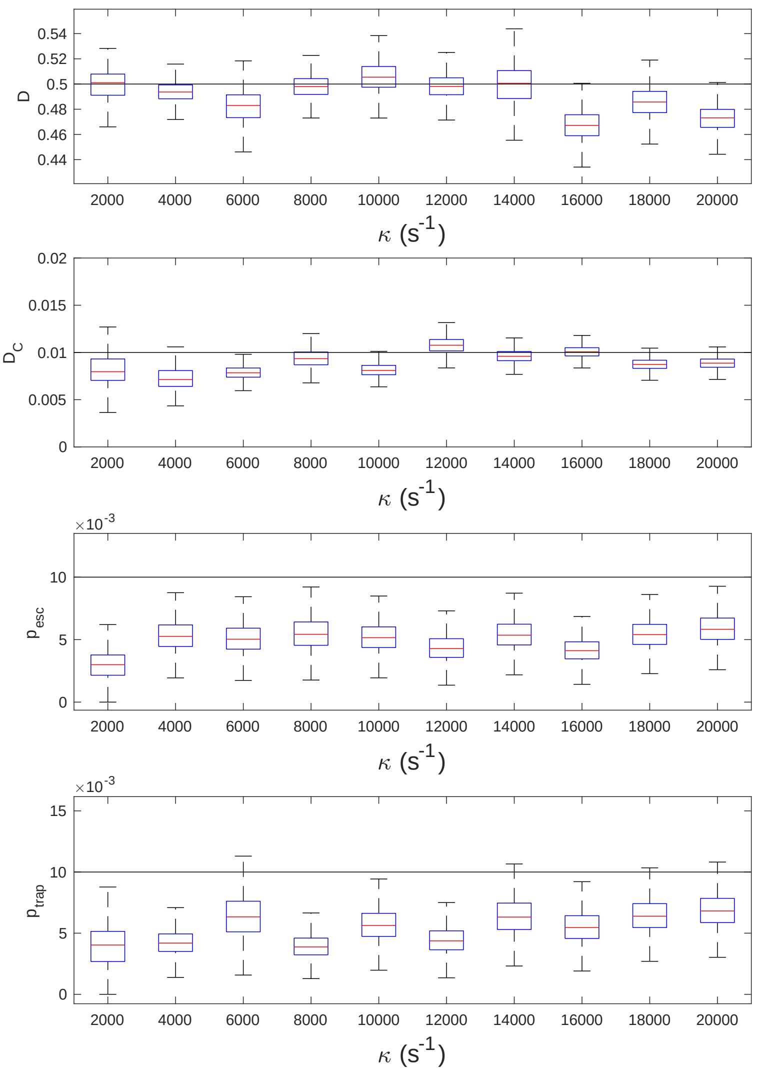

Parameter posterior distributions for harmonic potential well model applied to simulated trajectories with varying confinement strength κ. Black lines represent simulated parameter values ($D = 0.5\,\mu\text{m}^2\,\text{s}^{-1}$, $D_C = 0.01\,\mu\text{m}^2\,\text{s}^{-1}$, $p_{esc} = 0.01$, $p_{trap} = 0.01$).

13 Fig. S13: Varying confinement centre diffusion coefficient in simulations

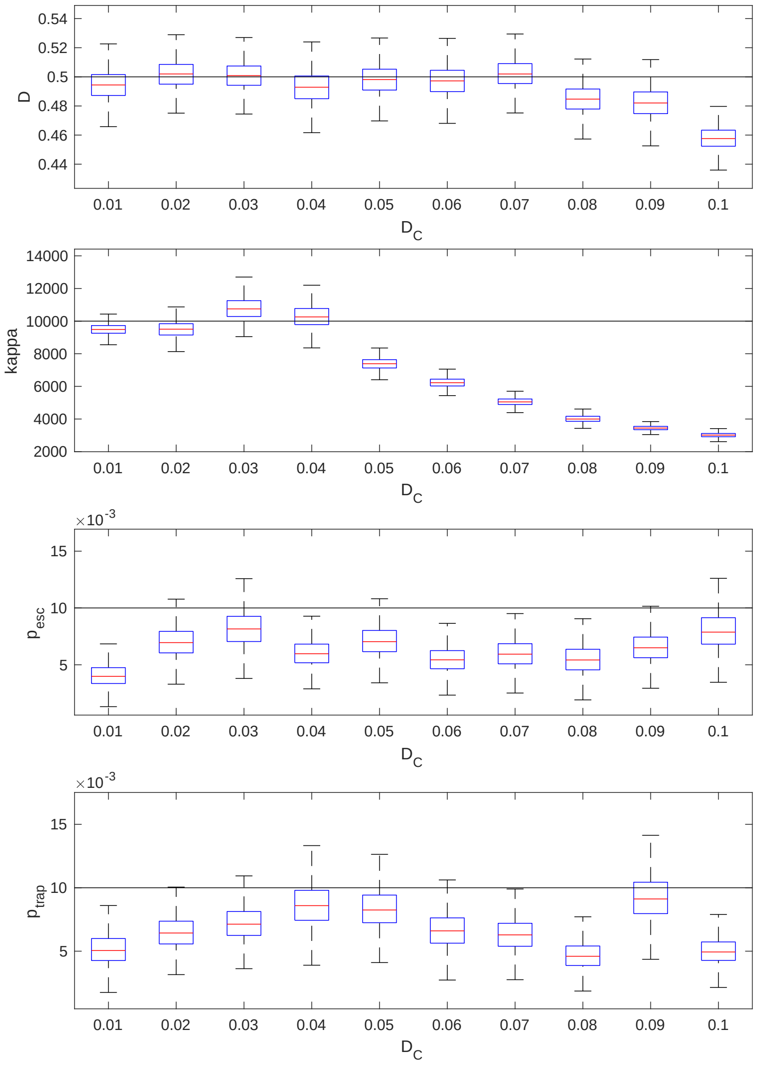

Parameter posterior distributions for harmonic potential well model applied to simulated trajectories with varying confinement centre diffusion coefficient D_C. Black lines represent simulated parameter values ($D = 0.5\,\mu\mathrm{m}^2\,\mathrm{s}^{-1}$, $\kappa = 10\,000\,\mathrm{s}^{-1}$, $p_{esc} = 0.01$, $p_{trap} = 0.01$).

14 Fig. S14: Fit of HPW model to a 20 nm AuNP/CTxB/GM1 trajectory in a model membrane on glass.

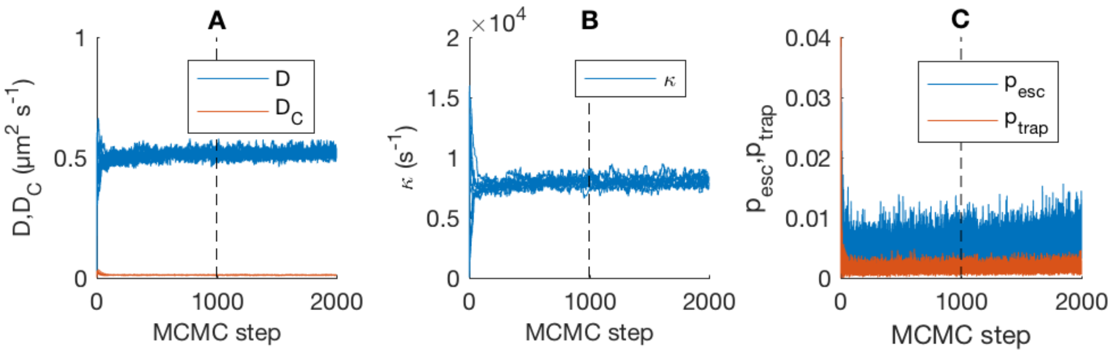

(A) MCMC chains for D (blue) and D_C (red). (B) MCMC chain for κ. (C) MCMC chains for p_{esc} and p_{trap}. For each parameter 12 independent MCMC runs are shown. Corresponding parameter posterior distributions are shown in Fig. 5 in the main text.

Fig. S15: 3D representation of HPW model applied to a 20 nm AuNP/CTxB/GM1 trajectory.

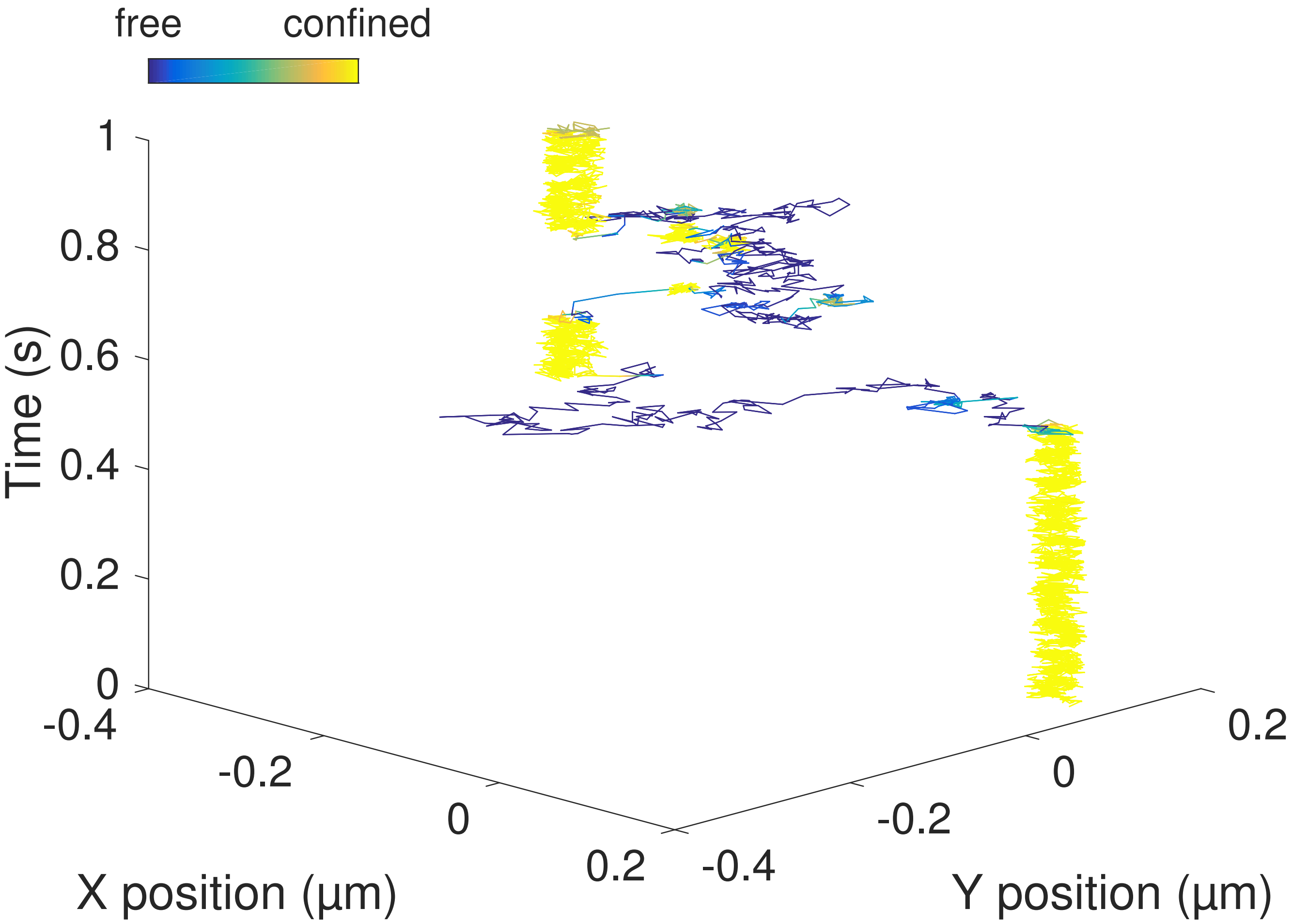

Colour represents the inferred hidden state. Corresponding hidden state and parameter posteriors are shown in Figs. 4 and 5 (main text) respectively.

16 Fig. S16: Parameter distributions across the population of trajectories.

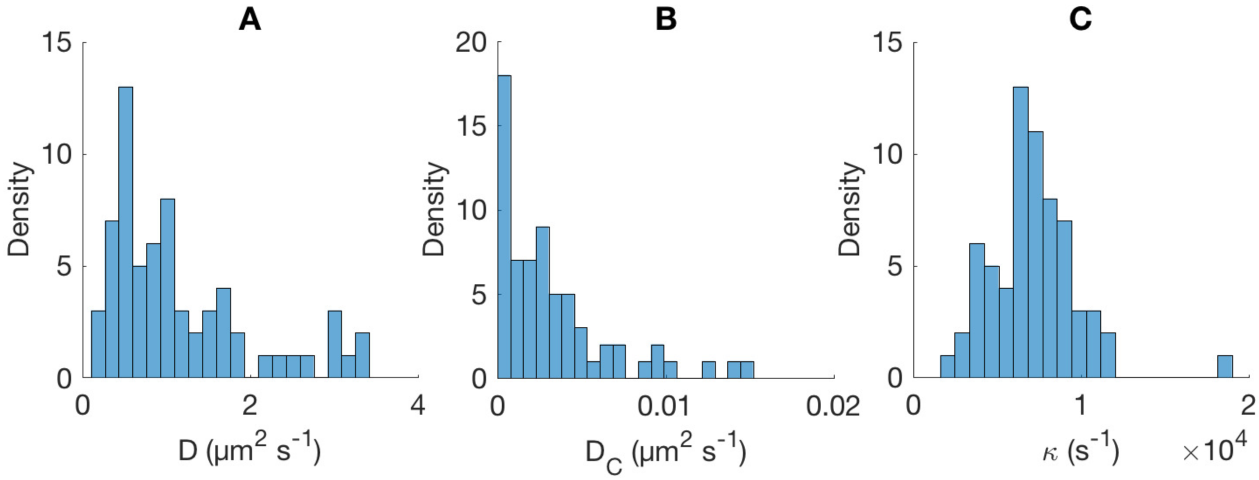

Histogram of the mean (posterior) parameters for HPW model applied to $20\,\mathrm{nm}$ AuNP/CTxB/GM1 on glass trajectories. A) Particle diffusion coefficient, B) harmonic well centre diffusion coefficient, C) harmonic well strength. D shows distinct deviation from being Gaussian ($p < 0.001$, Lilliefors test), although κ does not ($p = 0.21$).

17 Fig. S17: Variation in parameter posteriors across trajectories.

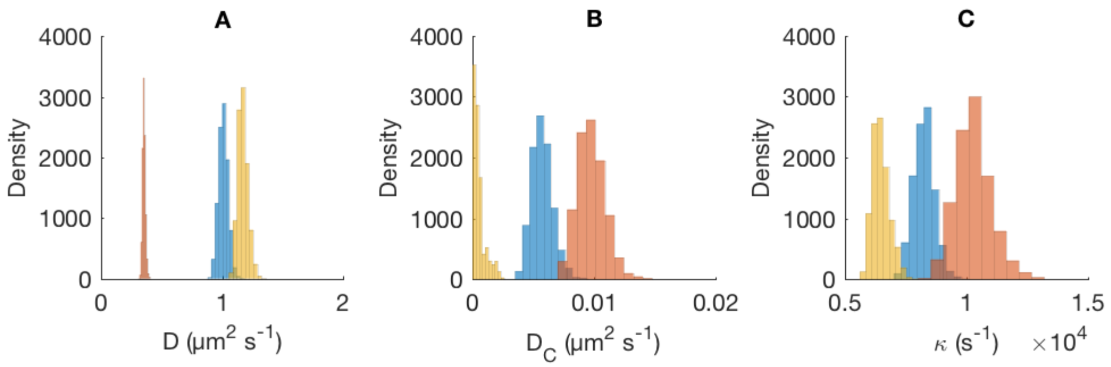

Histogram of parameter posterior samples for HPW model applied to three $20\,\mathrm{nm}$ AuNP/CTxB/GM1 on glass trajectories. A) Particle diffusion coefficient, B) harmonic well centre diffusion coefficient, C) harmonic well strength.

18 Fig. S18: Pooled confinement histograms for all 20 nm AuNP/CTxB/GM1 trajectories ordered by confinement size.

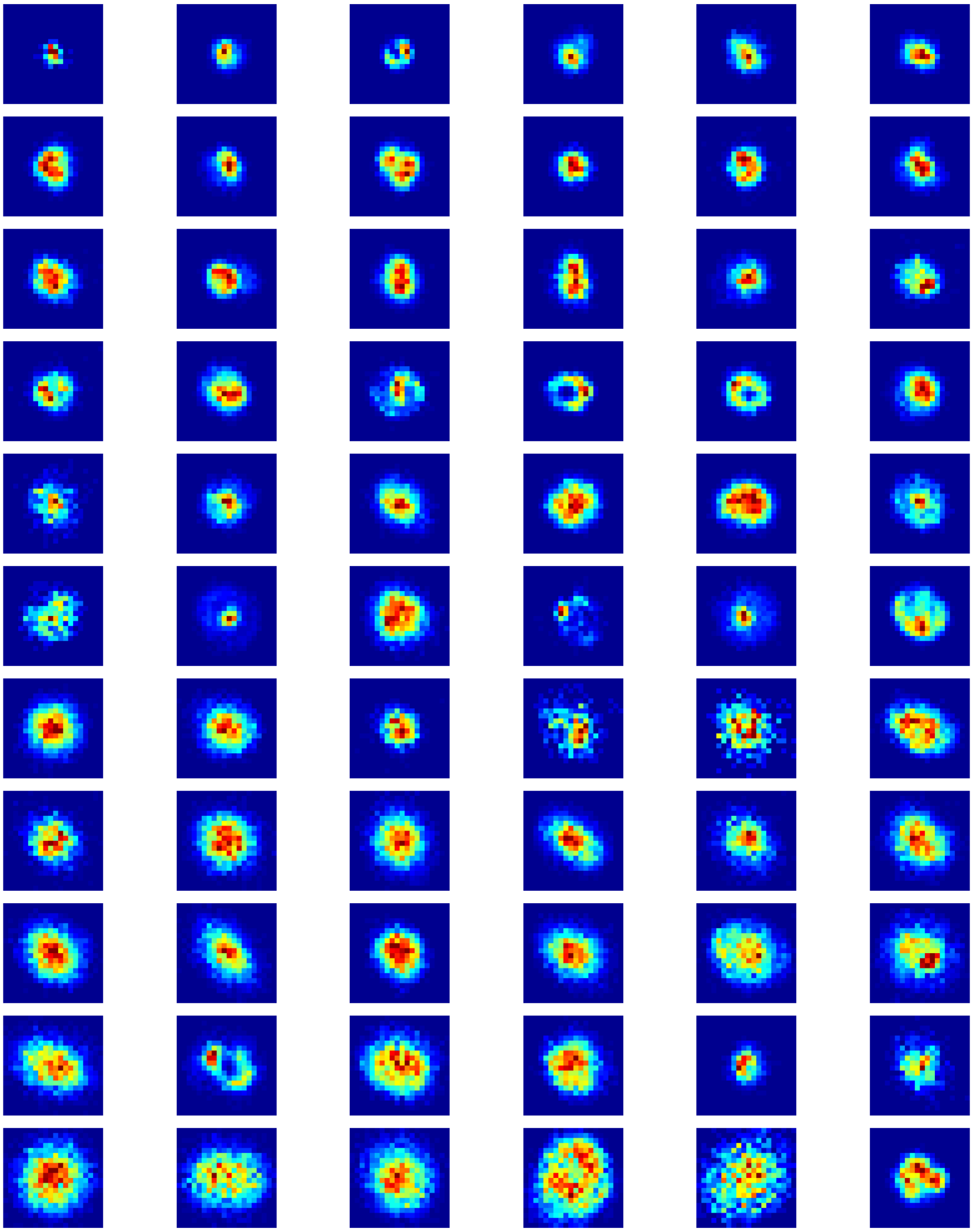

Histograms of particle positions pooled over confinement events for a trajectory. Trajectories are ordered (from smallest to largest) by the average of the mean confinement radius statistic ($\bar{R}_{lm}$) over all events (i.e. the order in Fig. 7C, main text). Confinement events were included based on the criteria in Table 1 in the main text, except that we included events revisiting a previous trapping zone (i.e. we did not enforce the condition that confinement event centres have to be $30\,\mathrm{nm}$ away from all previous centres in that trajectory). Each plot has side length $0.1\,\mu\mathrm{m}$.

19 Fig. S19: Pooled confinement histograms for all 20 nm AuNP/CTxB/GM1 trajectories ordered by spatial skew.

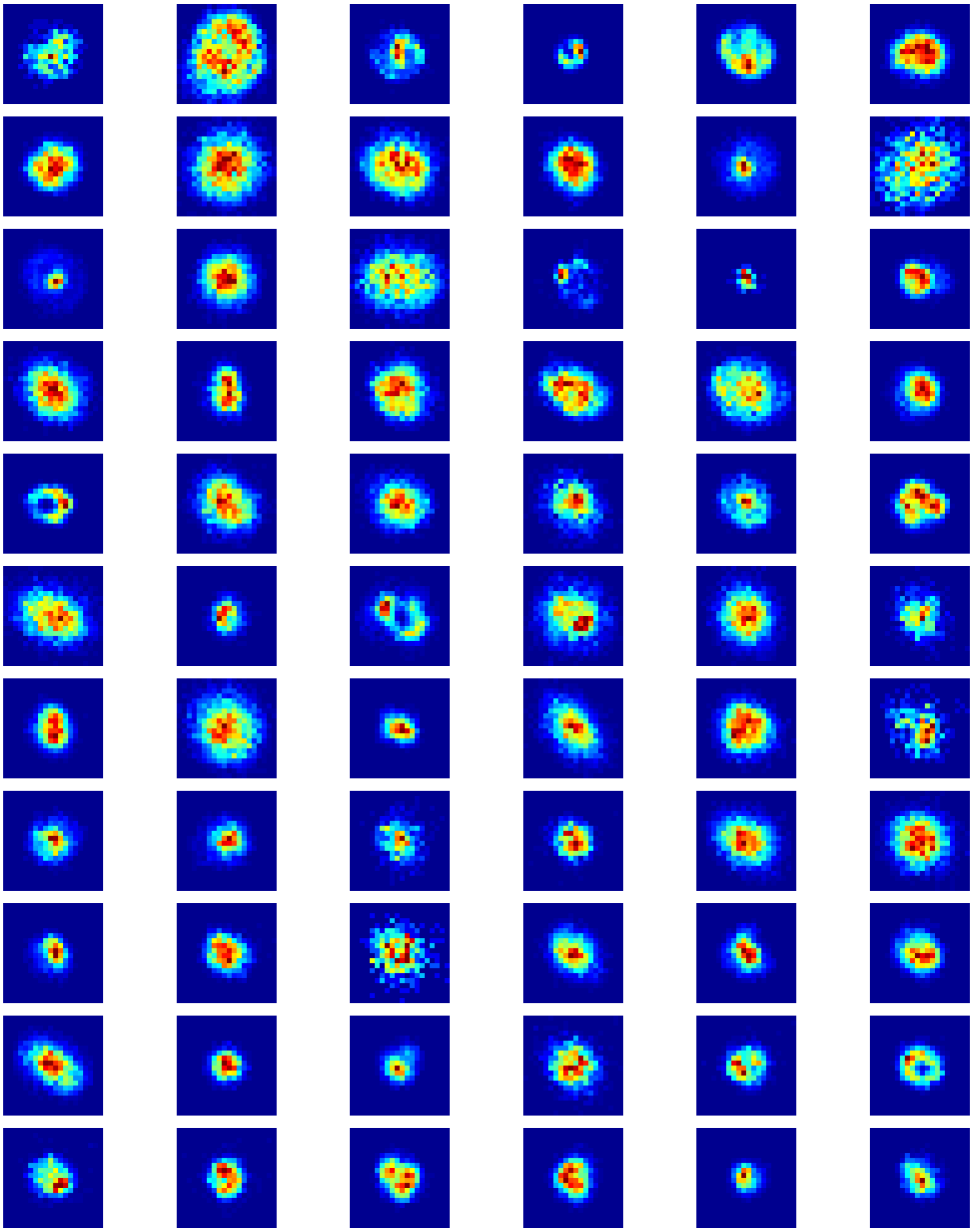

Histograms of particle positions pooled over confinement events for a trajectory. Trajectories are ordered (from smallest to largest) by the average of the radial skewness statistic (S_{lm}) over all events (i.e. the order in Fig. 7D, main text). Confinement events were included based on the criteria in Table 1 in the main text, except that we included events revisiting a previous trapping zone (i.e. we did not enforce the condition that confinement event centres have to be 30 nm away from all previous centres in that trajectory). Each plot has side length 0.1 μm.

20 Fig. S20: GM1 trajectories in SLBs on a mica substrate show no confinement.

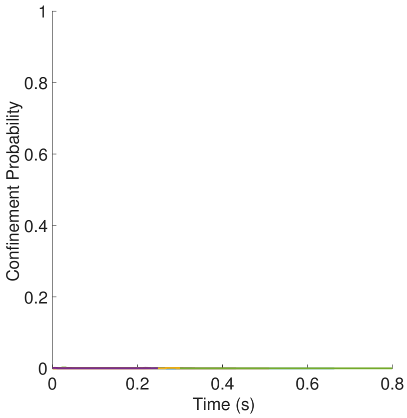

Inferred mean confinement state (average from 12 MCMC chains) from the harmonic potential well model applied to $40\,\mathrm{nm}$ AuNP/CTxB/GM1 trajectories in SLBs on mica. 18 trajectories of varying lengths (0.16-0.8s) each plotted in a different colour. The confinement (posterior) probability in all trajectories, was practically 0 at all times.
