# Supplementary material for: A Hidden Markov Model for Detecting Confinement in Single-Particle Tracking Trajectories
Source: Biophys J. 2018 Sep 13;115(9):1741–54. doi: 10.1016/j.bpj.2018.09.005 (PMC6226389; doi:10.1016/j.bpj.2018.09.005)
Supplement: Document S2. Article plus Supporting Material [file mmc2.pdf]

Biophysical Journal
Article

Biophysical Society

A Hidden Markov Model for Detecting Confinement in Single-Particle Tracking Trajectories

Paddy J. Slator[1,2] and Nigel J. Burroughs[3,*]
[1]Centre for Medical Image Computing and Department of Computer Science, University College London, London, United Kingdom; [2]Systems Biology Doctoral Training Centre, University of Warwick, Coventry, United Kingdom; and [3]Mathematics Institute, University of Warwick, Coventry, United Kingdom

ABSTRACT State-of-the-art single-particle tracking (SPT) techniques can generate long trajectories with high temporal and spatial resolution. This offers the possibility of mechanistically interpreting particle movements and behavior in membranes. To this end, a number of statistical techniques have been developed that partition SPT trajectories into states with distinct diffusion signatures, allowing a statistical analysis of diffusion state dynamics and switching behavior. Here, we develop a confinement model, within a hidden Markov framework, that switches between phases of free diffusion and confinement in a harmonic potential well. By using a Markov chain Monte Carlo algorithm to fit this model, automated partitioning of individual SPT trajectories into these two phases is achieved, which allows us to analyze confinement events. We demonstrate the utility of this algorithm on a previously published interferometric scattering microscopy data set, in which gold-nanoparticle-tagged ganglioside GM1 lipids were tracked in model membranes. We performed a comprehensive analysis of confinement events, demonstrating that there is heterogeneity in the lifetime, shape, and size of events, with confinement size and shape being highly conserved within trajectories. Our observations suggest that heterogeneity in confinement events is caused by both individual nanoparticle characteristics and the binding-site environment. The individual nanoparticle heterogeneity ultimately limits the ability of interferometric scattering microscopy to resolve molecule dynamics to the order of the tag size; homogeneous tags could potentially allow the resolution to be taken below this limit by deconvolution methods. In a wider context, the presented harmonic potential well confinement model has the potential to detect and characterize a wide variety of biological phenomena, such as hop diffusion, receptor clustering, and lipid rafts.

INTRODUCTION

Single-particle tracking (SPT) experiments directly observe the motion of single molecules and hence offer a powerful method to analyze the membrane environment. For instance, detection and characterization of heterogenous diffusion behaviors yields information on membrane structure (1,2). However, SPT methods require the molecule of interest to be tagged with a trackable label that is imaged over a number of time steps. A number of experimental design limitations constrain the amount of information that can be extracted from such data, including spatial accuracy, temporal resolution, and the tracking period. New technologies are capable of extending the trajectory length while retaining high sampling rates and high spatial resolution. For example, interferometric scattering microscopy (iSCAT) can generate very long (50,000 step) trajectories, with high spatial (<2 nm) and temporal (up to 500 kHz) resolution (3–6). However, a fundamental problem that impacts interpretation is the effect of the tag itself (7). This is particularly relevant for iSCAT because the gold nanoparticle (AuNP) tags are 20–40 nm in diameter, whereas spatial resolution is estimated to be ~2 nm for a 20-nm AuNP (4,5); relative movements between the AuNP and the bound GM1 will thus convolve with the movement of the GM1. For example, an iSCAT study on model membranes demonstrated both Gaussian-like and ring-like confinement events, which was ascribed to transient multivalent binding of the tag (4). Thus, to extend this technique to in vivo experiments, there is a need to deconvolve the tag signature from the environment signal. Failure to achieve this separation means that interpretation of the high-resolution dynamics measured by these techniques may be limited to the order of the tag's size.

Analysis of SPT data is not straightforward primarily because of the stochastic nature of diffusion. This has led

Submitted February 27, 2018, and accepted for publication September 4, 2018.

*Correspondence: n.j.burroughs@warwick.ac.uk

Editor: Markus Deserno.

https://doi.org/10.1016/j.bpj.2018.09.005

© 2018 The Authors.

This is an open access article under the CC BY license (http://creativecommons.org/licenses/by/4.0/).

Check for updates

to the development of a range of statistical methods that detect deviations from Brownian motion, such as mean-square displacement (MSD) (8–13) and confinement (14–19) analyses. A new breed of methods model switching of the movement dynamics between various dynamic states (20–24), often within a hidden Markov chain framework (25–31). For high-resolution data, the latter techniques can utilize the high level of information present in the trajectory to extract detailed motion characteristics and potentially infer underlying biophysical mechanisms.

However, the majority of existing hidden Markov approaches only incorporate changes in the particle diffusivity and (or) drift. Such methods can only approximate confinement through a change in the particle's effective diffusion coefficient. To our knowledge, the only exception is the work of Bernstein et al. (31) who explicitly model confinement using a hidden Markov model (HMM) within a maximal likelihood framework. In this article, we develop an HMM harmonic potential well (HPW) confinement analysis method using a Bayesian approach. Specifically, the particle moves between two states hidden to the observer: free diffusion with (to be determined) diffusion coefficient D and confinement in an HPW (center location and well strength to be determined). We developed a Markov chain Monte Carlo (MCMC) algorithm to infer model parameters and hidden states from a single trajectory. We tested the algorithm on simulated data, then applied it to previously published experimental iSCAT trajectories of GM1 lipids diffusing in model membranes (4). Specifically, a (20 or 40 nm) AuNP was coated in cholera toxin B subunits (CTxBs) by streptavidin binding, with each CTxB then binding 5 GM1 molecules in the lipid membrane to form an AuNP/CTxB/GM1 complex. In trajectories of 20 nm AuNP/CTxB/GM1 diffusing in model membranes on a glass substrate, we detected clear periods of trapping in wells of a mean radius of 18 nm with a mean trapping time of 0.024 s. However, we also observed inherent heterogeneities in both AuNP/CTxB/GM1 particles and trapping sites, which ultimately affect trajectory characteristics.

This article is organized as follows. In Methods, we introduce the HPW confinement model and an associated inference (MCMC) algorithm. The full derivation of the MCMC algorithm is described in Note S1 of the Supporting Materials and Methods. In Results, we demonstrate accurate inference of model parameters and hidden states on simulated trajectories, then apply the algorithm to iSCAT trajectories of AuNP/CTxB/GM1 diffusing in model membranes.

METHODS

We implemented the following methods in MATLAB (The MathWorks, Natick, MA). The source code, documentation, trajectory data, and working examples are freely available (https://doi.org/10.5281/zenodo.1405647).

HPW model

We developed a model for a particle that switches between a freely diffusing state and a confinement state localized around a slowly diffusing center. The state is encoded by a hidden variable z, with $z_i = 0$ if the particle is freely diffusing at time t_i and $z_i = 1$ if confined, where $i = 1..N$ denotes the time point (i.e., frame). The state z_{i+1} depends only on z_i with transition probabilities (constant frame rate):

$$\text{free}(z = 0) \underset{p_{trap}}{\overset{p_{esc}}{\leftrightarrows}} \text{confined}(z = 1), \tag{1}$$

where p_{trap} and p_{esc} are the per-frame probabilities of switching into and out of confinement, respectively. The probability of being in state z_{i+1} given state z_i is therefore as follows:

$$\pi(z_{i+1} \mid z_i) = \text{Bernoulli}\big(z_{i+1}; z_i(1 - p_{esc}) + (1 - z_i)p_{trap}\big), \tag{2}$$

where Bernoulli $(x; p)$ denotes the Bernoulli probability distribution with variable x and parameter p. In the free state, the particle diffuses freely with diffusion coefficient D. In the confined state, the particle is assumed to have a directed component to its diffusive motion, proportional to the distance from the well center C_i, i.e., the force is proportional to $X_i - C_i$ where X_i is the particle position at time t_i. (Note that X_i and C_i are two-dimensional (2D) vectors). During confinement, the center diffuses much slower than the particle itself (diffusion coefficient $D_C \ll D$). When the particle is free, C diffuses with diffusion coefficient D_{est}, where D_{est} is sufficiently high that the center can relocate between different confinement sites. The center is thus still present even when it is not affecting the particle. The stochastic differential equations for this model are as follows:

$$dX_t = -\kappa z_t(X_t - C_t)dt + \sqrt{2D}dW_t \text{ and} \tag{3}$$

$$dC_t = \sqrt{2(D_C z_t + D_{est}(1 - z_t))}dW_t^{(C)}, \tag{4}$$

where $W_t, W_t^{(C)}$ are independent Weiner processes. During confinement, X_t has Ornstein-Uhlenbeck (OU) dynamics with center C_t. We assume that switching can only occur at the sampling points. We also assume that C_t is slowly varying and therefore ignore its time dependence over the time step Δt. The frame-to-frame dynamics are hence,

$$\begin{aligned} X_{i+1} - X_i \sim N\Big(&z_i(C_i - X_i)(1 - e^{-\kappa\Delta t_i}), \\ &D\Big((1 - z_i)2\Delta t_i + \frac{z_i}{\kappa}(1 - e^{-2\kappa\Delta t_i})\Big)\Big), \text{ and} \end{aligned} \tag{5}$$

$$C_{i+1} - C_i \sim N(0, 2\Delta t_i(D_C z_i + D_{est}(1 - z_i))). \tag{6}$$

See Note S1 of the Supporting Materials and Methods for full details. If the step size is sufficiently small relative to the confinement strength ($\kappa\Delta t \ll 1$), an Euler-Maruyama approximation is justified, but if the particle explores the well over Δt, this OU solution is required. We refer to this discrete-time stochastic model as the HPW confinement model.

The model has two hidden states to be inferred at all trajectory time points $i = 1, .., N$: the state z_i (confined or free) and the position of the HPW center C_i when confined. There are also five parameters to be inferred: two diffusion coefficients (D and D_C), the strength of the HPW (κ), and two transition probabilities (p_{esc} and p_{trap}). D_{est} is treated separately because it only weakly affects the trajectory and does not affect the likelihood or parameter estimates provided it is sufficiently high. Fig. 1 *A* shows a simulated HPW model trajectory. In the simulation, we include a drift

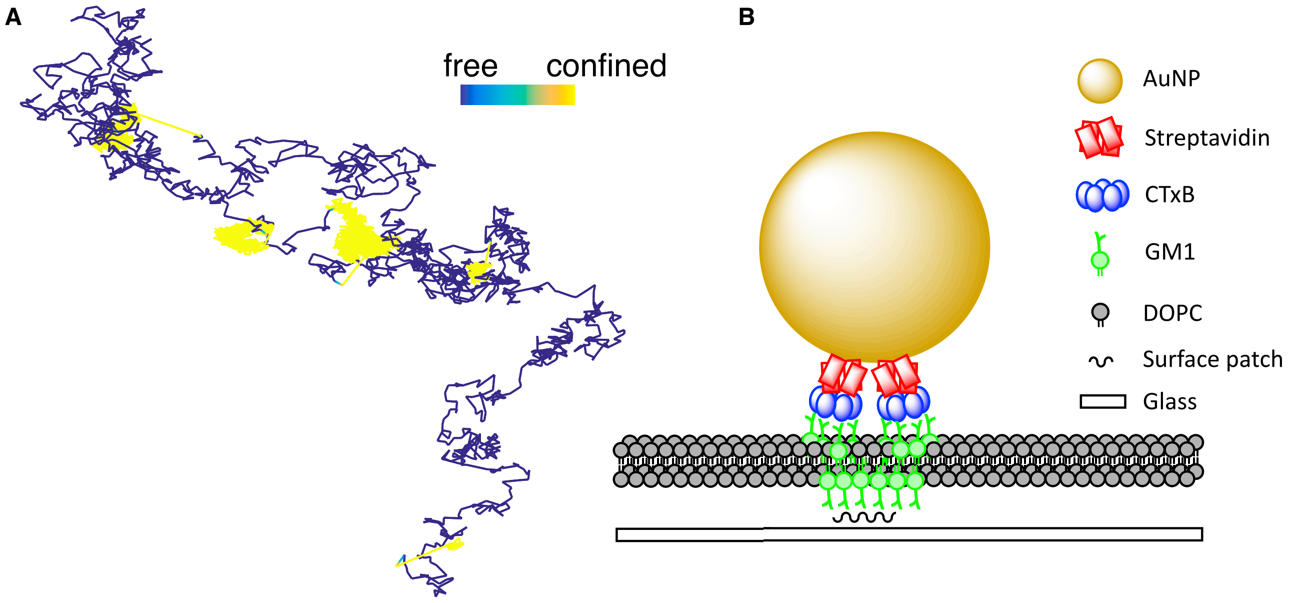

FIGURE 1 Simulated harmonic potential well (HPW) model trajectory. (*A*) A simulated trajectory colored by state. Model parameters are $D = 0.5\ \mu m^2 s^{-1}$, $D_{est} = 0.5\ \mu m^2 s^{-1}$, $D_C = 0.01\ \mu m^2 s^{-1}$, $\kappa = 3000\ s^{-1}$, $p_{esc} = 0.001$, $p_{trap} = 0.002$, time step 2×10^4 s, and $N = 5000$ frames. The simulation was performed using Eq. 5 and a modified version of Eq. 6 as discussed in the main text. Trajectory colored blue, denoting free diffusion or yellow, denoting the confined state. Color bar length, 0.1 μm. (*B*) A schematic of AuNP/CTxB/GM1 complex in 1,2-dioleoyl-sn-glycero-3-phosphocholine (DOPC) lipid bilayer, based on figure 5 in (4).

term for the center so that C tracks X when not confined. This ensures that C is close to X when the particle switches from free diffusion to confinement, and therefore confinement zones remain within a reasonable field of view. This tracking of X by C is not included in the inference algorithm because diffusion alone is sufficient to allow the Markov chain to find high-probability paths.

MCMC sampler

There are a number of MCMC samplers for linear switching models in the literature; the main distinction is whether variables are integrated out using an inverse Wishart prior (32), or a Markov chain incorporating all variables is used. The latter approach allows greater control of prior information, including use of uninformative priors, whereas the Wishart distribution, motivated by computational convenience, imposes a dependence between variable correlations and scale, which is a concern for inference (33). We developed an MCMC algorithm (Supporting Materials and Methods, Note S1) for the full system of variables to fit the HPW model (Eqs. 5 and 6) to 2D trajectory data, $\mathbf{X} = \{X_i, t_i\}_{i=1}^{N}$. We chose uninformative priors for all parameters except for the transition probabilities, where we use an informative prior to restrict rapid switching between states (details in Note S1 of the Supporting Materials and Methods). For an SPT trajectory, the algorithm samples the posterior distribution, $\pi(\theta, \mathbf{z}, \mathbf{C} \mid \mathbf{X})$, giving K samples of the parameters $\theta^{(k)} = \{D^{(k)}, D_C^{(k)}, \kappa^{(k)}, p_{esc}^{(k)}, p_{trap}^{(k)}\}_{k=1}^{K}$ and hidden states $\{\mathbf{z}^{(k)}, \mathbf{C}^{(k)}\}_{k=1}^{K}$. Here, for each sample k, $\mathbf{z} = \{z_i\}_{i=1}^{N-1}$ and $\mathbf{C} = \{C_i\}_{i=1}^{N-1}$ are the set of hidden states and center locations (2D vectors) throughout the trajectory.

We determined convergence of the MCMC sampler by calculating the Gelman potential scale reduction factor (PSRF) (34), considering a run converged provided the PSRF was below a threshold in all variables, set to 1.2 on experimental trajectories. The MCMC run length was increased up to a maximum of 4×10^5 steps on trajectories that failed the convergence criteria on shorter runs.

GM1 molecules diffusing in model membranes

We applied the MCMC algorithm to previously published iSCAT SPT data (4), where CTxB-coated AuNPs were introduced to a 1,2-dioleoyl-sn-glycero-3-phosphocholine lipid bilayer containing 0.03% GM1 lipids (Fig. 1 *B*). A confinement event corresponds to an interaction between an AuNP/CTxB/GM1 complex on the upper leaflet with a lower leaflet GM1 that is immobilized on a glass surface. This was previously referred to as "inter-leaflet coupling and molecular pinning" (4). Both Gaussian and non-Gaussian confinement events were observed; we investigated these events in greater detail using our HPW model.

The data set includes 71 trajectories of 20-nm AuNP/CTxB/GM1 diffusing in a model membrane on a glass substrate and 18 trajectories of 40-nm AuNP/CTxB/GM1 in a model membrane on a mica substrate. There is a dynamic error in the localization accuracy at the 50-kHz sampling rate resulting in apparent superdiffusive behavior, which we removed by sub-sampling down to 5 kHz (Figs. S1 and S2; Note S2 of the Supporting Materials and Methods). We also removed trajectory artifacts because of multiple AuNPs in the focal area (Supporting Materials and Methods, Note S2). The MCMC algorithm did not converge on five 20-nm AuNP/CTxB/GM1 trajectories (PSRF convergence criteria of 1.2) leaving a set of 66 trajectories for further analysis. MCMC runs on all 18 40-nm AuNP/CTxB/GM1 on mica trajectories converged.

Thresholding hidden states for lifetime analysis

For each trajectory, at each time point i, we computed the probability of confinement $\pi(z_i|\mathbf{X})$ from the MCMC posterior distribution samples. This probability distribution is concentrated near 0 and 1 on experimental trajectories (only 2.7% of the state probabilities were between 0.2 and 0.8; Fig. S3), indicating high confidence in confinement state estimates. To annotate the trajectory by state, we define the binary signal, $z_i^B = 0$ or $z_i^B = 1$, for free diffusion and confinement respectively, using a threshold of 0.5 on $\pi(z_i|\mathbf{X})$. We then identify confinement events as a series of

ones in the (posterior) binary state vector $\mathbf{z}^B$ and free diffusions as a series of zeros, allowing event lifetimes (and per-event spatial statistics) to be computed. When considering event lifetimes, we exclude those containing either the first or last time point of the trajectory, because the full event is not witnessed, hence the state lifetime is unknown.

Confinement event profiling

To analyze confinement events in 20-nm AuNP/CTxB/GM1 trajectories, we utilized spatial statistics (including the mean confinement radius and radial skewness, defined in Table 1) based on the Euclidean distance between the particle and the confinement center. We calculate these statistics for all events of at least 0.01 s (50 frames). Unlike the event lifetime analysis, we allow events that contain either the first or last (or both) time points. Furthermore, we compute statistics including events revisiting a previous confinement zone where applicable (details in Table 1). These restrictions left a set of 271 confinement events when excluding repeat events and 427 when including them. The number of events within a trajectory ranges from 1 (there were six examples in which the particle remained trapped for the entire trajectory) to 11 (without repeats) or 25 (with repeats).

RESULTS

MCMC on simulated data

The HPW model sampler was extensively tested on simulated data. Figs. 2 and 3 show an MCMC run on the simulated trajectory of Fig. 1 *A*. The parameter posteriors are consistent with the true (i.e., simulation) values (Fig. 2). When confined, the inferred center closely tracks the simulated center (Fig. 3, *A* and *B*), and every confinement event is accurately inferred (Fig. 3 *C*). The inferred model parameters are independent of the algorithm parameter D_{est} in both the noiseless case (Fig. S5) and in the presence of static localization error at the same level as the 20-nm AuNP/CTxB/GM1 trajectories (Fig. S6); p_{trap}, and p_{esc} are typically underestimated because of their informative priors. There also appears to be a small underestimation in the confinement strength κ in the presence of localization error, which we further investigated by increasing the localization error's SD (Fig. S7). This revealed a clear trend, with an increase in confinement strength underestimation bias with localization error. However, the effect is relatively small at the noise level (SD 2.7 nm) of the experimental data presented in this article. Performance was robust to changes in trajectory length and number of events (Figs. S8 and S9). Estimation of D, D_C, κ, $\mathbf{z}$, and switching rates were robust to the time series subsampling rate (Figs. S10 and S11); in particular, most events were still detected even with a 10-fold subsampling (Fig. S11). Parameter estimation is also robust to changes in confinement strength κ (Fig. S12). However, we found that confinement strength estimation accuracy decreases dramatically when the confinement center diffusivity D_C approaches 10% of the free diffusivity D (Fig. S13), reflecting the degeneracy of the problem when $D_C \approx D$.

MCMC on 20-nm AuNP/CTxB/GM1 on glass trajectories

An example of the model fit is shown in Fig. 4, with the segmented trajectory shown in Fig. 4 *E*. Fig. 5 shows the associated parameter posterior estimates. Particle state (confined or free diffusion) is well determined, with state probabilities near zero or one (Fig. 4 *C*). The parameter estimates for D and κ are tight (low relative SD), whilst the diffusion coefficient of the center is very low, $D_C = 0.010 \pm 0.0009\ \mu\text{m}^2\text{s}^{-1}$ (mean $\pm$ SD) compared to $D = 0.52 \pm 0.017\ \mu\text{m}^2\text{s}^{-1}$, indicating near complete immobilization of the well. The inferred position of the well center is also practically stationary in both coordinates during periods of confinement consistent with immobilization (Fig. 4, *A* and *B*). As an independent measure of changes in mobility, we estimated the effective local diffusion coefficient (Fig. 4 *D*), which demonstrates a clear shift at around 0.5 s (i.e., the first inferred switch point). By color coding the trajectory according to the probability of being confined per frame (Fig. 4 *E*), we can extract periods of confinement with non-Gaussian occupation profiles (Fig. 4, *F* and *H*). In this trajectory, we observed that one confinement zone is visited twice (Fig. S15) and that the repeat confinements had remarkably similar occupation profiles (Fig. 4, *G* and *H*). The probability per frame of switching is reasonably well inferred (Fig. 5 *C*) despite the small number of events. The probability of escape from a confinement zone is smaller than the probability of trapping, reflecting the short periods of time that the AuNP/CTxB/GM1 complex undergoes free diffusion.

Applying our MCMC algorithm to the 66 trajectories, we obtain parameter estimates across the population (Fig. S16). The mean value of D over all trajectories was 1.15 $\pm$ 0.106 $\mu\text{m}^2\text{s}^{-1}$ (mean $\pm$ standard error (SE); population SD 0.86 $\mu\text{m}^2\text{s}^{-1}$); MSD analysis (using the @msdanalyzer package (35)) gave a smaller estimate,

TABLE 1 Calculation of Confinement Event Statistics

Statistic	Calculation
Confinement radius	$\mathbf{R}_{lm} = \{R_i\}_{i \in T_{lm}}, R_i = \lVert X_i - \overline{C}_{lm} \rVert$
Mean confinement radius	$\overline{R}_{lm} = \frac{1}{M_{lm}} \sum_{i \in T_{lm}} R_i$
Radial skewness	$S_{lm} = \frac{\frac{1}{M_{lm}} \sum_{i \in T_{lm}} (R_i - \overline{R}_{lm})^3}{\left[\sqrt{\frac{1}{M_{lm}} \sum_{i \in T_{lm}} (R_i - \overline{R}_{lm})^2}\right]^3}$
Radial mean-median distance	$\lvert \overline{R}_{lm} - \widehat{R}_{lm} \rvert$
Radial SD	$\sqrt{\mathrm{var}[\mathbf{R}_{lm}]}$

We denote the time points of the mth trapping event in the lth trajectory T_{lm}. Events have associated particle positions $\mathbf{X}_{lm} = \{X_i\}_{i \in T_{lm}}$ and harmonic well center positions $\mathbf{C}_{lm} = \{C_i\}_{i \in T_{lm}}$. The mean posterior harmonic well center is given by $\overline{C}_{lm} = (1/M_{lm}) \sum_{i \in T_{lm}} C_i$, where M_{lm} is the number of time points in T_{lm}. To remove events that revisit a previous trapping zone, we did not include events if $\overline{C}_{lm}$ was within 30 nm of a previous confinement center ($\overline{C}_{ln}$, $n < m$) within trajectory l. $\lVert . \rVert$ denotes the Euclidean distance, and $\widehat{R}_{lm}$ denotes the median.

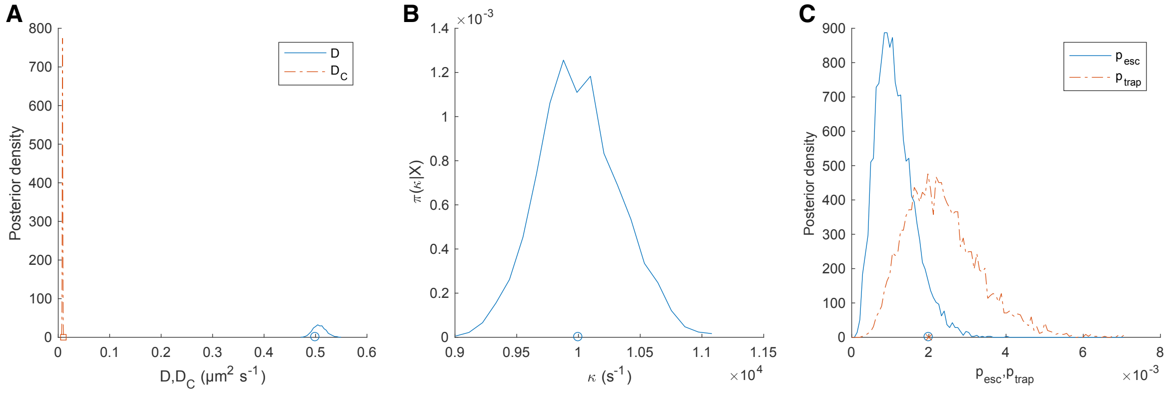

FIGURE 2 Posterior parameter distributions of the HPW model for a simulated trajectory. (*A*) The posterior distribution for D (*blue, solid line*) and D_C (*red, dashed line*), with simulation values indicated (*circles*) (*B*) The posterior for κ and simulation value (*circle*). (*C*) The posterior for p_{esc} (*blue, solid line*) and p_{trap} (*red, dashed line*), with simulation values (cross, *circle respectively*). The trajectory is as in Fig. 1. MCMC priors are as in Note S1 of the Supporting Materials and Methods. Corresponding MCMC runs are shown in Fig. S4. Data are based on the pooling of five independent chains of 2000 steps with a 1000-step burn-in. MCMC priors are as in Note S1 of the Supporting Materials and Methods. To see this figure in color, go online.

0.0525 ± 0.017 $\mu m^2 s^{-1}$ (mean $\pm$ SE). This difference reflects the fact that MSD does not account for confinement, which is the dominant state, whereas our diffusion coefficient estimate does. Our Bayesian analysis provides estimates of parameter confidence per trajectory, which are in fact substantially smaller than the spread between trajectories (Fig. S17); specifically, the ratio of the population variances of D and κ are 257 and 59 times larger than the average trajectory posterior variances, respectively. This indicates the presence of system variability, giving rise to trajectory heterogeneity. To understand its cause, we investigate whether heterogeneity is manifest in the

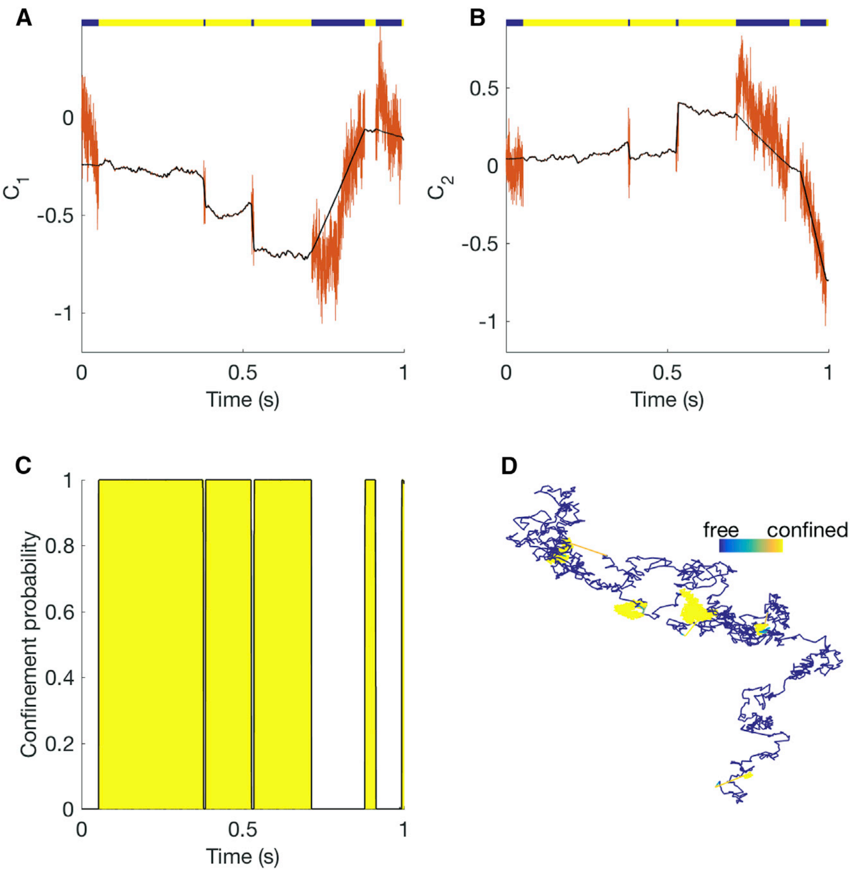

FIGURE 3 Hidden state inference for the HPW model for a simulated trajectory. (*A* and *B*) The mean inferred position of the harmonic potential center in x and y directions (*black*) and simulated (true) center (*red*). The colored line at the top represents the following particle states: free diffusion (*blue*) and confinement (*yellow*). (*C*) The inferred confinement probability (*black line*) and simulated (true) confinement state (*yellow area*). (*D*) The trajectory is colored by mean inferred confinement state, from $\pi(z_i|\mathbf{X}) = 0$ (*blue*, free) to $\pi(z_i|\mathbf{X}) = 1$ (*yellow*, confined). Color bar length, 0.1 μm. MCMC is as in Fig. 2.

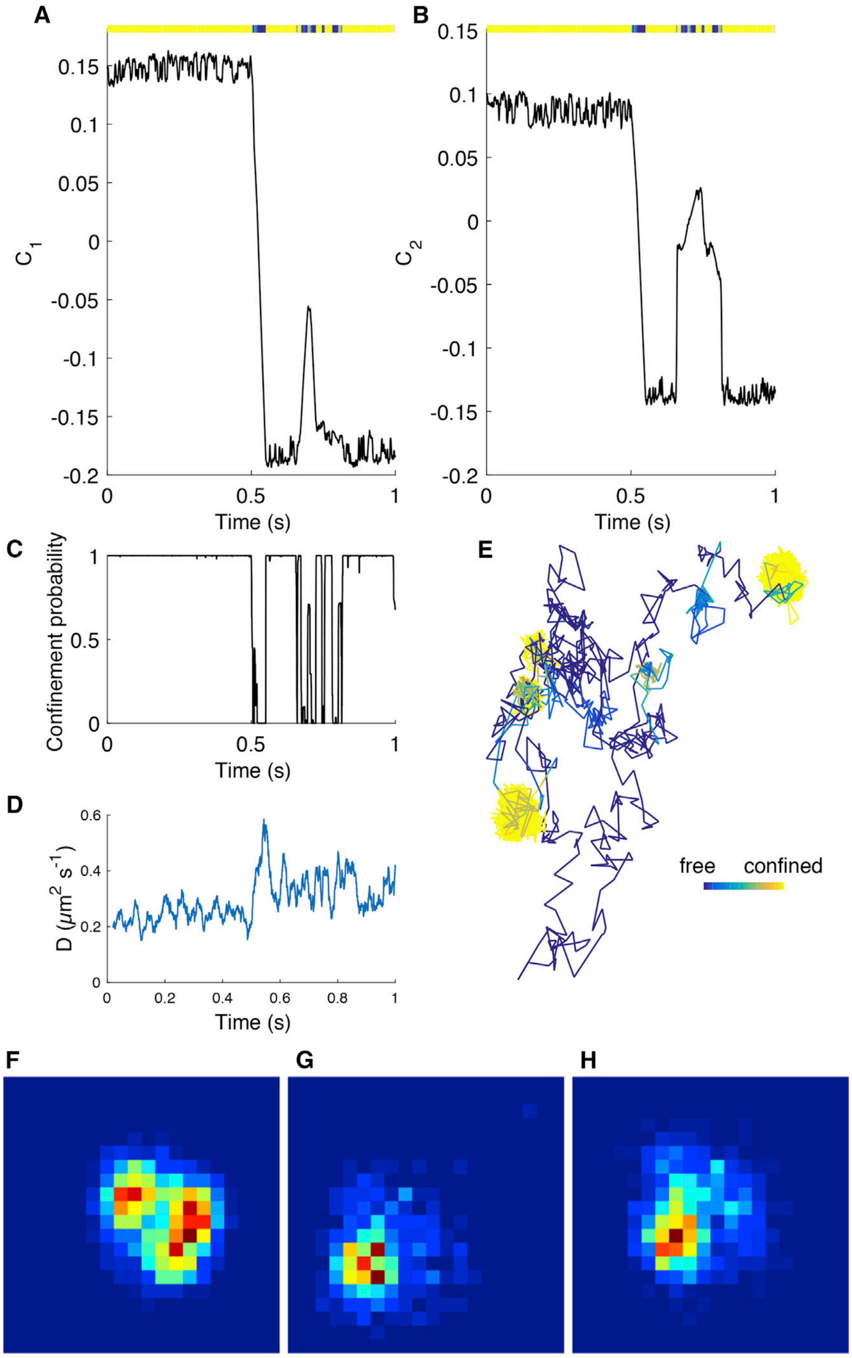

FIGURE 4 Hidden state inference for the HPW model applied to a 20-nm AuNP/CTxB/GM1 trajectory. (*A* and *B*) The mean inferred position of the HPW center *C* (*x*, *y* components) and upper colored bar representing $\pi(\mathbf{z}|\mathbf{X})$, (color scale goes from $\pi(z_i|\mathbf{X}) = 0$ (*blue*, free) to $\pi(z_i|\mathbf{X}) = 1$ (*yellow*, confined)). (*C*) The inferred mean confinement state and (*D*) moving average of local maximal likelihood diffusion coefficient estimate (window size: 100 subsampled frames). (*E*) The trajectory colored by mean inferred confinement state (color bar length, 0.1 μm). (*F–H*) Density-colored 2D spatial histograms of confinement events. The two events in (*G*) and (*H*) are spatially colocated. Data is based on the pooling of 10 independent chains. MCMC priors and convergence criteria are as in Note S1 of the Supporting Materials and Methods.

confinement events of individual trajectories, specifically the size, shape, and lifetime of these events.

Lifetime and shape analysis of confinement events

The mean confinement state lifetime (as defined in Methods) is 0.024 s, but there is a large variation in event lifetimes across trajectories (Fig. 6, *A* and *B*) and significant heterogeneity across trajectories ($p = 0.02$, Kruskal-Wallis test, 1779 events across 60 trajectories). The lifetimes of free-diffusion events (mean 0.002 s) did not show significant heterogeneity across trajectories, ($p = 0.86$, Kruskal-Wallis test on 60 trajectories, 1770 events). Further, we examined if the population of lifetimes across trajectories conform to an exponential waiting time model, i.e., whether switching between states obey first-order kinetics. A quantile-quantile plot demonstrates that there is a distinct deviation from an

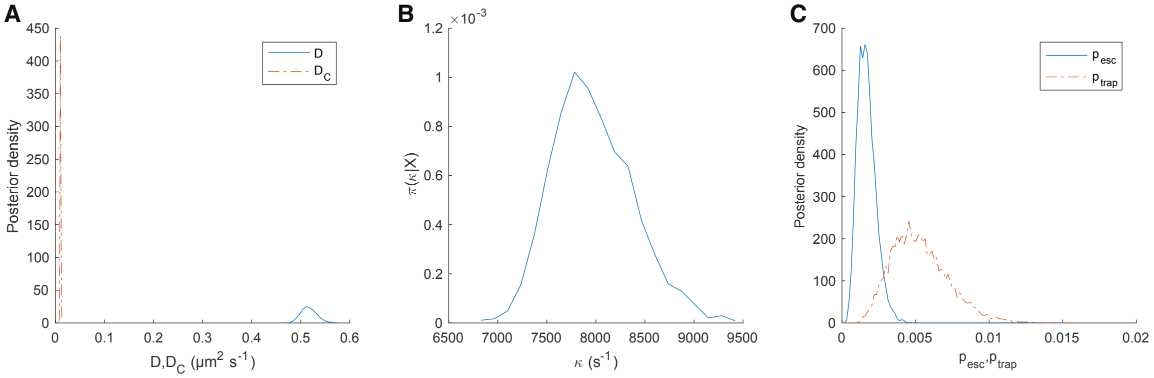

FIGURE 5 Posterior parameters of the HPW model applied to a 20-nm AuNP/CTxB/GM1 trajectory. (*A*) Posterior distributions for D (*blue, solid line*) and D_C (*red, dashed line*). (*B*) The posterior for κ. (*C*) The posteriors for p_{esc} (*blue, solid line*) and p_{trap} (*red, dashed line*). Distributions consist of samples pooled from 12 independent runs. Corresponding MCMC chains are shown in Fig. S14. Trajectory and MCMC runs are as in Fig. 4. To see this figure in color, go online.

exponential distribution fit (mean event time $\mu = 0.024$ s); specifically, there are a far higher proportion of longer trapping events, indicative of heterogeneity. A mixture of two exponentials is a better fit (Fig. 6 *D*) suggesting that the confinement events derive from a heterogeneous population with at least two components with short and long average lifetimes. The minor population of long lifetime events are dispersed over trajectories (Fig. 6 *B*); in particular,

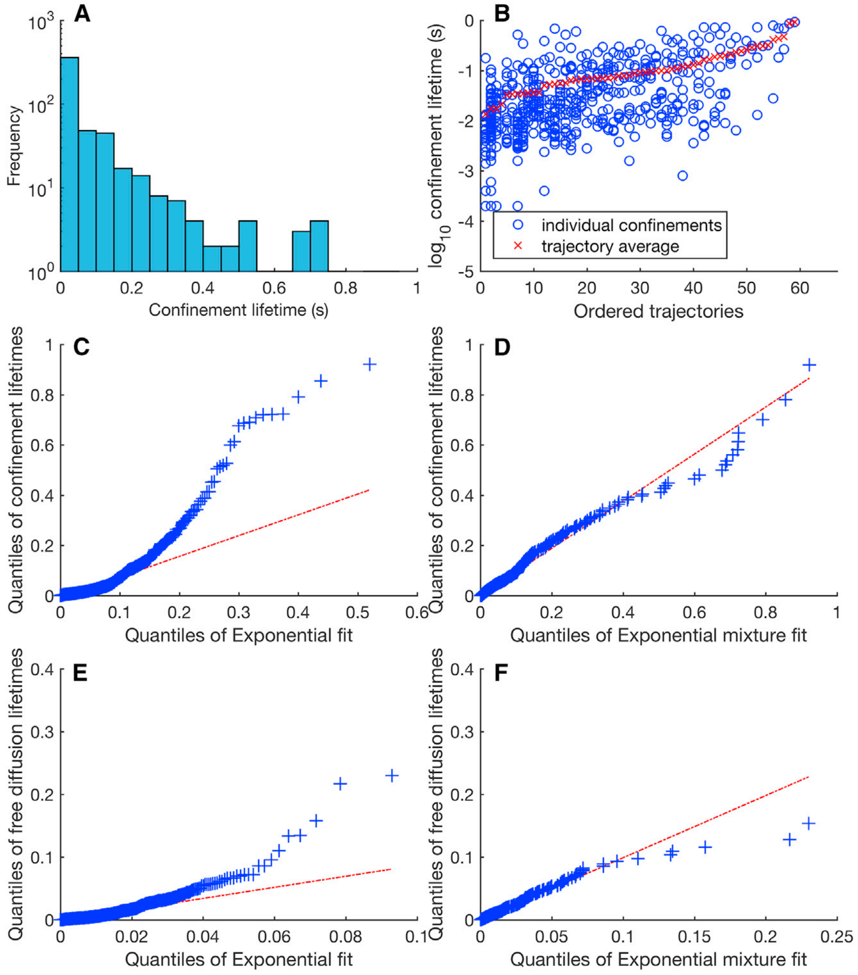

FIGURE 6 Confinement event lifetimes are not exponentially distributed. (*A*) A histogram of all confinement lifetimes ($n = 1959$ events). (*B*) A scatterplot of confinement lifetimes against trajectories ordered by mean confinement lifetimes. (*C–F*) Quantile-quantile plots of state lifetimes against exponential fits. (*C*) Confinement events against the exponential distribution ($\mu = 0.024$ s, $R^2 = 0$) and (*D*) confinement events against samples ($n = 10^4$) from a mixture of two exponentials ($\mu_1 = 0.004$ s, $\mu_2 = 0.1$ s; weights 0.80 and 0.20, respectively; $R^2 = 0.98$) are shown. (*E*) Free diffusion lifetimes ($n = 2011$ events) against the exponential distribution ($\mu = 0.002$ s, $R^2 = 0.98$) and (*F*) free diffusion lifetimes against samples ($n = 10^4$) from a mixture of two exponentials ($\mu_1 = 0.002$ s, $\mu_2 = 0.01$ s; weights 0.99 and 0.01, respectively; $R^2 = 0.98$) are shown. The red line is an extrapolated linear fit to the first and third quantiles. Plots include all confinement events except those that contained the trajectories' first or last time point. To see this figure in color, go online.

trajectories are not split into two groups with long and short mean confinement times. In contrast, the free diffusion state lifetimes closely follow a mono-exponential distribution (Fig. 6, *E* and *F*).

We next analyzed confinement event shape using the spatial statistics defined in Table 1. The mean confinement radius over all trajectories is 18 nm, comparable to the size of the AuNP, although estimator inflation is likely to be present (36). The mean radial skewness is 0.88; for comparison, a 2D Gaussian distribution gives a radial displacement (from the mean) that is Rayleigh distributed with skew $(2\sqrt{\pi}(\pi-3)/(4-\pi)^{3/2}) \approx 0.63$. Mean confinement radius and radial skewness show a wide distribution of values across confinement events (Fig. 7, *A* and *B*), with significant (one-way analysis of variance: mean confinement radius $p = 1.2 \times 10^{-6}$; radial skewness $p = 9.1 \times 10^{-4}$; 271 events grouped by 66 trajectories) heterogeneity across trajectories (Fig. 7, *C* and *D*). Confinement event spatial histograms for all 66 20-nm AuNP/CTxB/GM1 trajectories, ordered by the average within-trajectory mean confinement radius (Fig. S18) and average radial skewness (Fig. S19) demonstrate the wide variety of confinement shapes.

The observed heterogeneity in confinement time, size, and (previously reported (4)) shape raises two key questions:

1) Does the shape of confinement events determine their lifetime?
2) Does heterogeneity predominately arise from a mechanism operating at individual confinement sites (local environment dependent) or at the level of trajectories (AuNP/CTxB/GM1 nanoparticle dependent)?

To probe the relationship between confinement event shape and lifetime (the first question), we examined their correlation. We found no correlation between confinement state lifetime and mean confinement radius (Fig. 7 *E*; Table S1) but a weak (and significant) negative correlation between lifetime and radial skewness (Fig. 7 *F*; Table S1). This suggests that the mixed-exponential nature of the

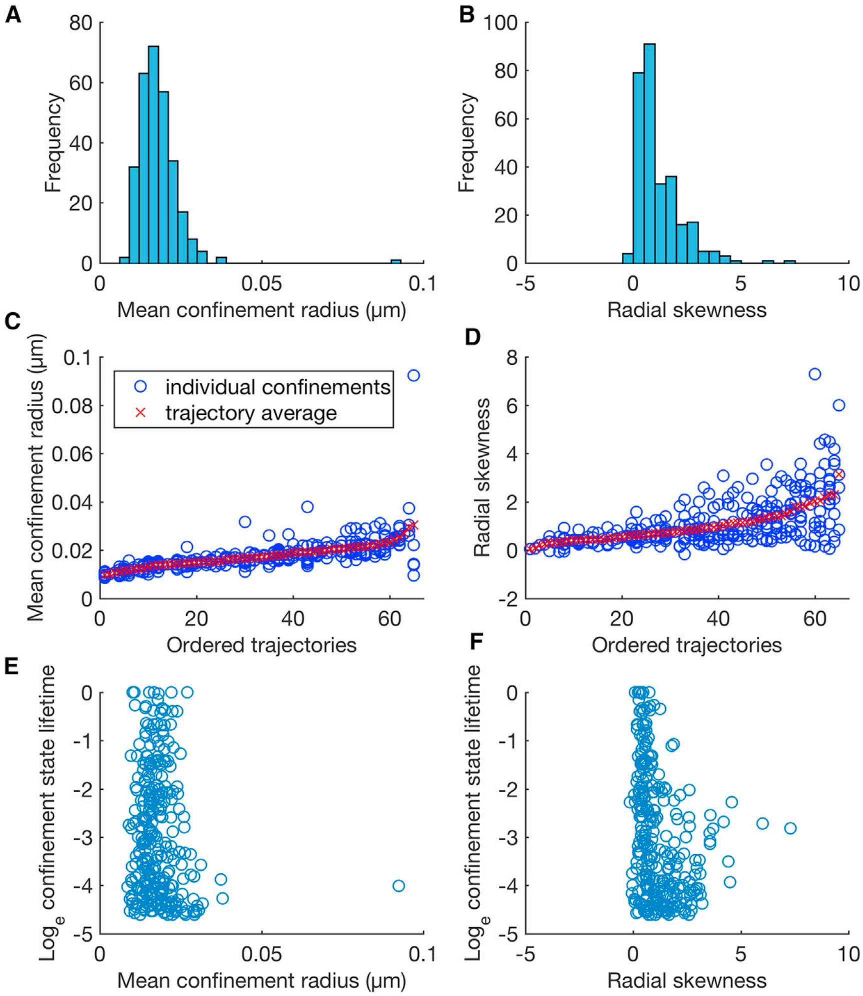

FIGURE 7 Shape statistics for confinement events in 20-nm AuNP/CTxB/GM1 trajectories. (*A* and *B*) Histograms over confinement events. (*C* and *D*) Spatial statistics for all confinement events, ordered by the average within trajectory statistic. Plots include all confinement events of at least 0.01 s, with events revisiting a previous trapping zone removed (giving 271 events). (*E* and *F*) Scatterplots of state lifetime against the given spatial statistic for the same 271 confinement events. To see this figure in color, go online.

binding lifetime is only weakly related to the shape of the binding event, i.e., these arise from different physical mechanisms.

Regarding the second question, the heterogeneity analysis above (see also Fig. 7, *C* and *D*) indicates that confinement events are statistically more similar within trajectories than across trajectories. Additionally, the ratio of the mean variance within trajectories to the variance across all events is 0.6 for both mean confinement radius and radial skewness (Table S2). To determine which confinement statistic is most strongly conserved within trajectories, we clustered events by each confinement statistic and quantified the similarity of events within single trajectories (Fig. 8). Confinement size is the most conserved, followed by confinement lifetime when excluding events revisiting a previous confinement zone (Fig. 8 *A*). Incorporating revisiting events dramatically improves the conservation of confinement size and shape statistics relative to lifetime (Fig. 8 *B*); this suggests that, although shape is conserved, confinement time is variable between events at the same location. This shape conservation at the same site is evident from the confinement event spatial histograms for long events (Fig. 9). Of note, the mean-median distance statistic failed to show significant heterogeneity across trajectories ($p = 0.06$, one-way analysis of variance) reflecting its lack of conservation when excluding events revisiting a previous confinement zone (Fig. 8 *A*) but was as conserved as lifetime when all events were included (Fig. 8 *B*). Thus, in answer to the second question, heterogeneity arises at both the trajectory- and confinement-site level, with different effects on confinement lifetime and shape. This suggest that nanoparticle confinement events are described by two degrees of freedom.

Analysis of 40-nm AuNP/CTxB/GM1 trajectories on mica

As a control, we analyzed 18 trajectories of 40-nm AuNP/CTxB/GM1 diffusing in supported lipid bilayers (SLBs) on a mica substrate. The previous analysis demonstrated that no confinement for this treatment was present (4). We applied our HPW model MCMC algorithm to this data and detected no confinement events (Fig. S20); the posterior confinement probability was <0.01 for all time, in all trajectories. The mean D was 1.2048 ± 0.09 $\mu m^2 s^{-1}$ (mean $\pm$ SE), comparable to 20-nm AuNP/CTxB/GM1 on glass trajectories (1.15 ± 0.20 $\mu m^2 s^{-1}$). The mean MSD-derived (with @msdanalyzer (35)) D was 0.87 ± 0.12 $\mu m^2 s^{-1}$. These values are in closer agreement than for the 20-nm AuNP/CTxB/GM1 on glass data set, which is expected because of the lack of confinement. However, we again observed trajectory heterogeneity in the diffusion coefficients with a ratio of population variance/mean trajectory variance of 123, indicative of individual AuNP-dependent diffusion coefficients.

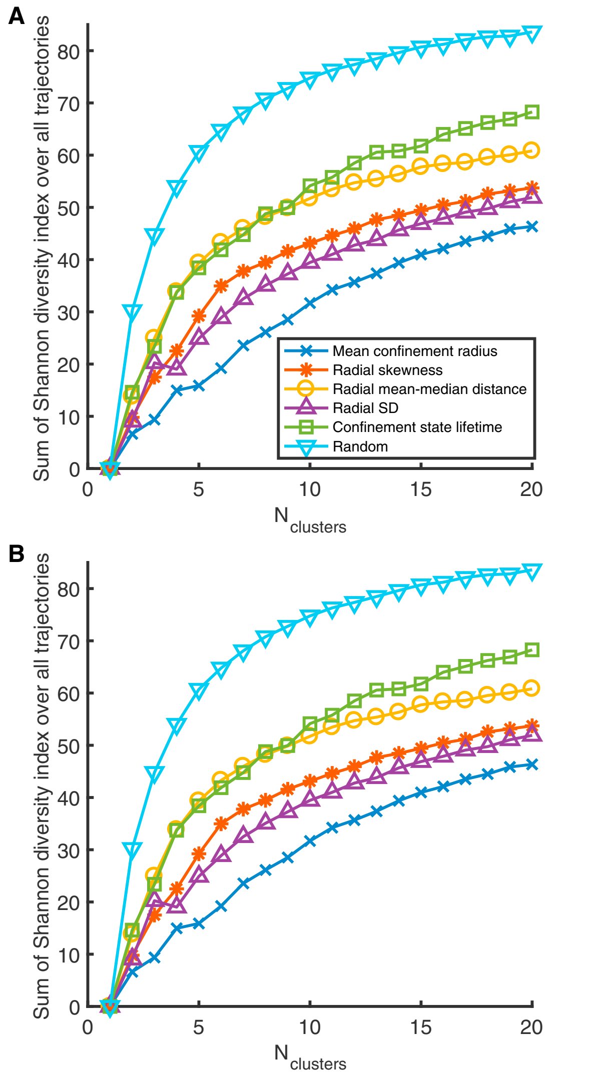

FIGURE 8 Clustering of confinement event statistics across individual confinement events were clustered (k-means++ algorithm (42) with squared Euclidean distance metric) based on event statistics. For each trajectory, *l*, the Shannon diversity index, $H_l = \sum_{j=1}^{N_{clusters}} p_j \log p_j$, was calculated ($p_j$ is the proportion of the events in trajectory *l* that appeared in cluster *j*). The sum of the Shannon diversity index over all trajectories is then a measure of the dissimilarity of events within trajectories (the lower the Shannon index the higher the similarity). The event statistics (shown in the legend) are defined in Table 1. For each choice of $N_{clusters}$, 50 separate clusterings were performed (because the k-means++ algorithm stochastically assigns initial values for cluster centroids), and the sum of the Shannon diversity index was averaged over these clusterings. (*A*) The clustering of events was obtained as described in "Confinement Event Profiling" in the main text, except with events containing the first or last time points excluded (214 events total). (*B*) The clustering of events is the same as (*A*), except with events that revisited a previous confinement zone included. To see this figure in color, go online.

DISCUSSION

We developed a Bayesian algorithm to infer an HPW confinement HMM and used it to partition SPT trajectories into periods of free diffusion and confinement. When

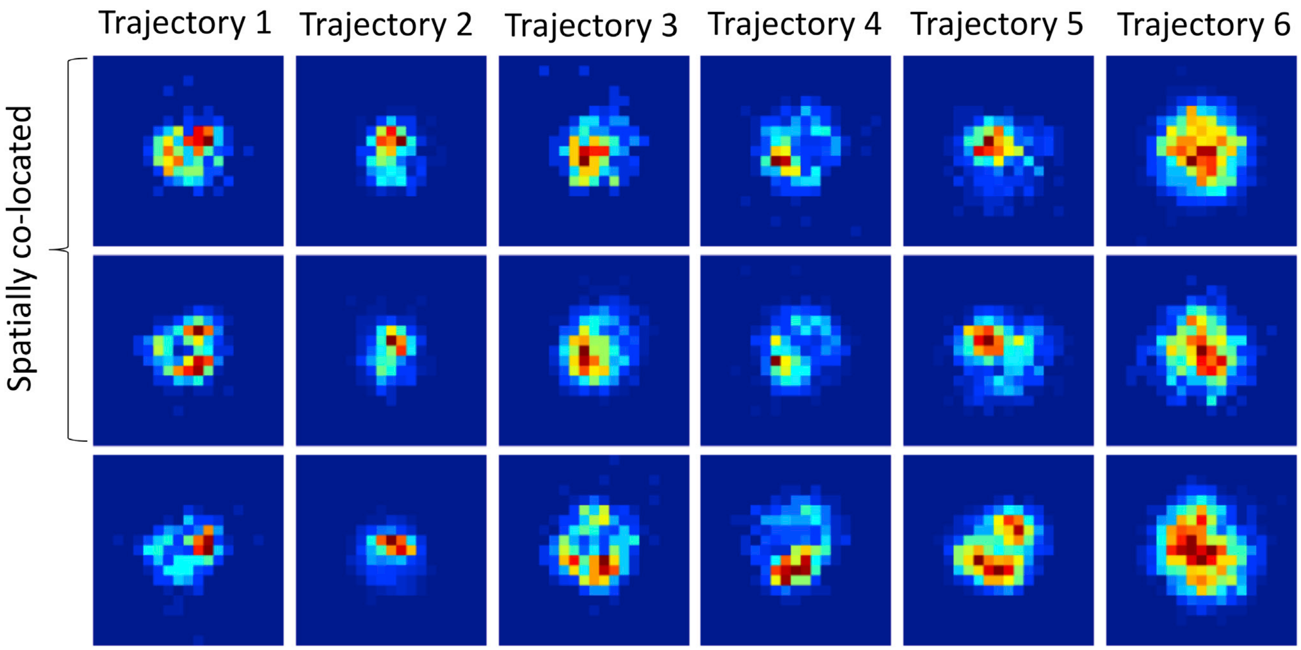

FIGURE 9 Spatial conservation of confinement events revisiting the same site. Each column shows particle position histograms for two spatially colocated confinement events and one event at a different location in the same trajectory. The spatially colocated confinement events are distinct, i.e., the particle moved away from the trapping zone between the displayed events. Each plot has a side length of 0.1 μm.

applied to experimental AuNP/CTxB/GM1 trajectories, we detected clear periods of confinement and free diffusion (Fig. 4). It was previously proposed that confinement event shape heterogeneity (Gaussian versus non-Gaussian confinement) in this data set was due to transient multivalent binding of the tag (4). Our analysis of confinement events attained using the HPW model revealed the following heterogeneity trends:

(1) Confinement size and shape are conserved within trajectories (Fig. 7, C and D), and repeat events at the same site show similarities (Figs. 5, G and H and 9).
(2) Confinement event lifetimes are heterogeneous across trajectories and comprise a mixture of at least two exponentials with short (4 ms) and long (100 ms) mean lifetimes (Fig. 6, C and D).
(3) Spatial heterogeneity and lifetime heterogeneity are effectively uncorrelated, suggesting they arise from different mechanisms.

Based on these observations, we propose a refinement to the transient multivalent tag-binding hypothesis. Namely, the characteristics of individual confinement events are determined by the following two factors: the size and geometry of the GM1 platform on the lower leaflet (determining residence times) and the number and distribution of CTxB complexes bound to the surface of the AuNP (determining size and shape of confinement event) (Fig. 1 *B*).

These dependencies are consistent with CTxBs remaining attached to the surface via GM1s throughout the entire trajectory (Fig. 10); this is supported by the high affinity of the CTxB/GM1 bond with a dissociation rate in SLBs of $(2.8 \pm 0.1) \times 10^{-4}$ s^{-1}, giving a mean binding lifetime of 3.6×10^{4} s (37). We propose that differences in the geometry of bound CTxB on the surface of the nanoparticle causes trajectory-conserved variation in the observed confinement as follows: tightly packed (or single) CTxBs have more freedom to "wobble" (Fig. 10 *B*), and broadly spaced, multiple (bound) CTxBs have less freedom (Fig. 10 *C*), giving a large, respectively small confinement radius for binding events. Additionally, non-Gaussian confinement events occur when there is a second (or potentially multiple) CTxB/GM1 attachment that is not immobilized, which restricts movement to a rotation or nonuniform "wobbling" around the immobilized binding site (Fig. 10 *D*). These hypotheses are consistent with the fact that there are around 25 CTxBs per 20-nm AuNP (4); it is expected that there will be variability in both their number and spatial distribution. We observe a strong correlation of the diffusion coefficient with the mean confinement radius ($r = 0.61$; Fig. 11 *A*), but not with the confinement event lifetime ($r = 0.27$; Fig. 11 *B*). This is consistent with the hypothesis that with more attachments, the AuNP experiences higher drag, whereas the mean confinement radius decreases because of stronger geometric constraints. Our analysis thus suggests that variation in the number and spatial configuration of bound CTxB contributes to the nature of the AuNP interaction with the upper leaflet of the bilayer, thereby giving each individual AuNP/CTxB/GM1 complex a confinement signature (Figs. 8, S18, and S19) and diffusion coefficient, with the latter also being evident in the confinement-free 40-nm AuNP data.

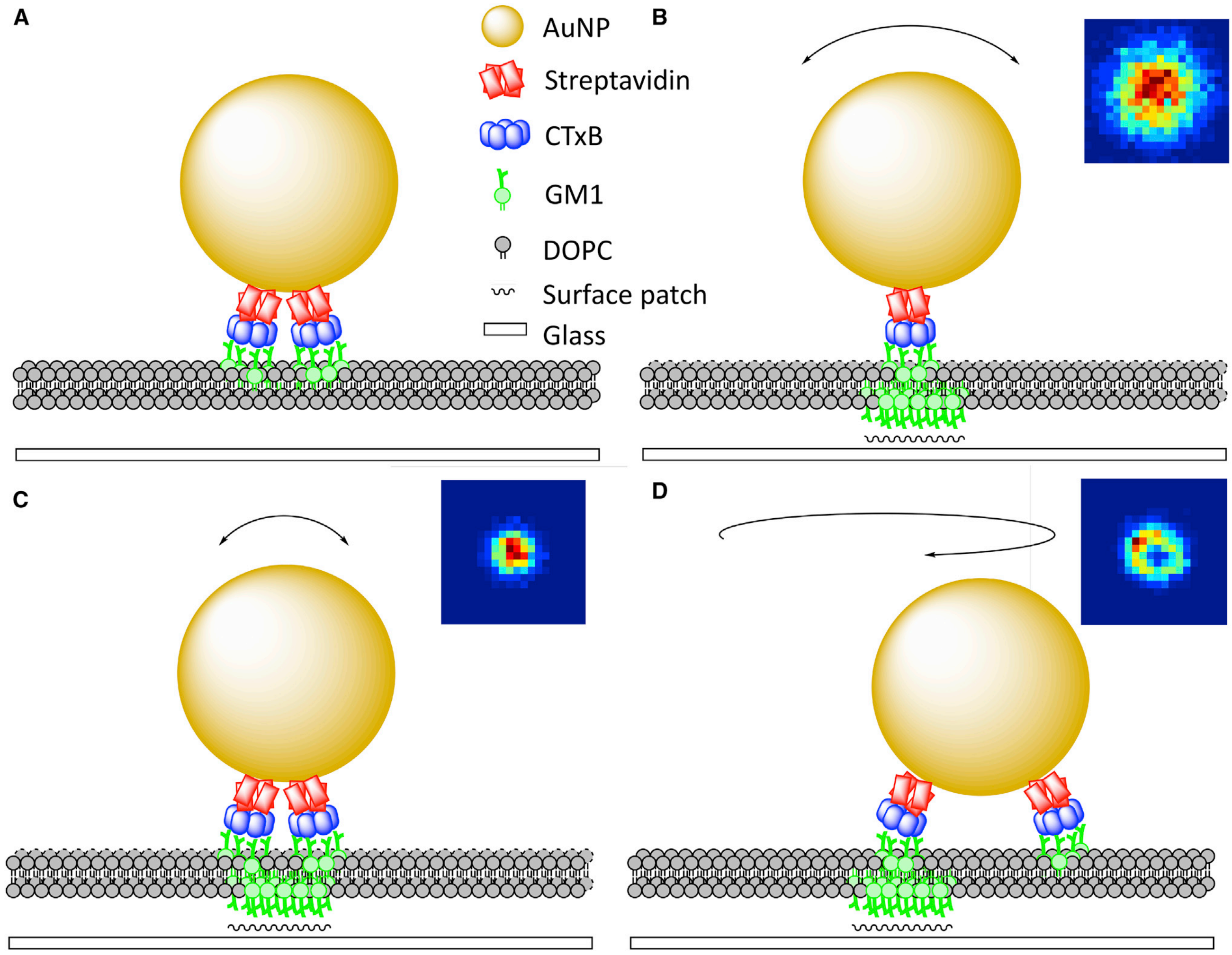

FIGURE 10 Schematic of AuNP/CTxB/GM1 structures leading to Gaussian and non-Gaussian confinement profiles. (*A*) Free diffusion, (*B*) wide Gaussian-like confinement, (*C*) narrow Gaussian-like confinement, and (*D*) non-Gaussian confinement. Insets in (*B*)–(*D*) are example histograms of particle positions pooled over confinement events within selected trajectories (e.g., Figs. S18 and S19). Insets have a side length of 0.1 μm. The schematic is based on a figure in (4). To see this figure in color, go online.

The characteristics of the lower-leaflet GM1 platform contribute a confinement site dependence, with conservation of shape and size upon revisiting the same site (Figs. 8 and 9). On the other hand, confinement lifetime at the same site is variable. The GM1 in the lower leaflet is immobilized by hydroxyl pinning sites on the glass surface, with these sites having an estimated size of <10 nm (4). However, aggregation of GM1 with domain sizes of 15–60 nm in SLBs has been observed in atomic force microscopy experiments (38). Large sites consist of more aggregated GM1 in the lower leaflet. Our mean confinement radius is 18 nm, which would comprise both AuNP/GM1/CTxB nanoparticle degrees of freedom around the binding site and displacements of the GM1 platforms between the leaflets. This suggests that either pinning sites are small, or no relative movement is possible. Larger pinning sites may trap multiple CTxB molecules on the AuNP, leading to more Gaussian behavior as rotational degrees of freedom are lost and possibly longer (on average) trapping times. This could be the cause of the negative correlation between non-Gaussian confinement shape and event lifetime (the lower the radial skewness statistic, the longer the typical confinement time (Fig. 7 *F*)). However, the double exponential mixture distribution of confinement lifetimes cannot be explained by these mechanisms. Mean lifetime does not partition by trajectories (with long event lifetimes being distributed throughout the trajectories (Fig. 6 *B*)) suggesting a random process is responsible. The simplest explanation is that binding of the AuNP/CTxB/GM1 at the pinning sites is heterogeneous, e.g. there could be a multistep binding sequence with the second (long lifetime) step proceeding in only a fraction of the binding events. We note that the six trajectories that remain confined throughout are a third population, because even on the long-lifetime distribution, observing binding events of 1 s or longer is negligible (probability 4.5×10^{-5}).

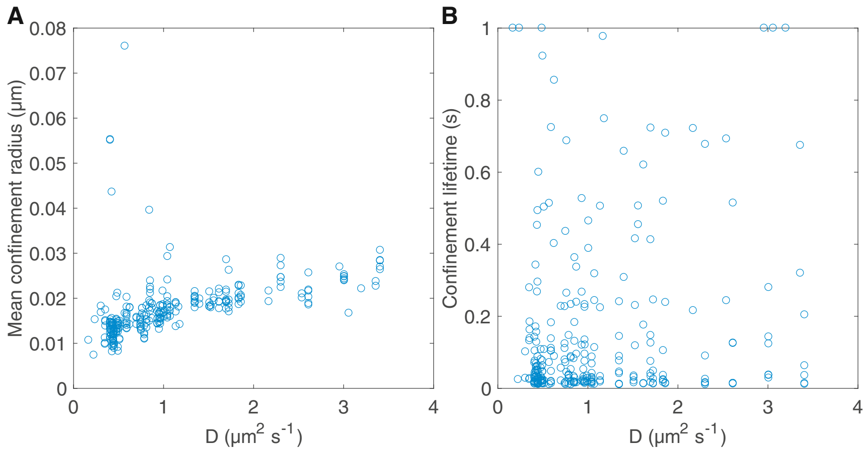

FIGURE 11 Correlation between D and confinement statistics. (*A*) The mean confinement radius and (*B*) confinement lifetime. Plots include all confinement events of at least 0.01 s. To see this figure in color, go online.

Outlook and future work

Analysis of SPT trajectories with HMMs has advantages over other methods for detecting confinement in single trajectories. In particular, they do not rely on tuning algorithm parameters through a comparison with Brownian motion. Additional parameters (such as the confinement strength κ, center C, and switching times as inferred here) can also be extracted, which allows for interpretation and comparison of confinement event characteristics across and within trajectories. However, appropriate HMMs are necessary for successful analysis. Specifically, models must approximate well the behavior of different dynamic states in the data. For instance, confinement is often associated with a decrease in the effective diffusion coefficient, suggesting that models that switch diffusivities (25–30) should also be able to detect confinement in these iSCAT particle trajectories. However, we found that a two-state diffusion coefficient switching HMM (30) could not segment these trajectories (data not shown). This implies that the effective diffusion coefficient of the AuNP/CTxB/GM1 complex does not change sufficiently during confinement events. The effective diffusion coefficient under confined Brownian motion is dependent on the temporal and spatial resolution of the data, suggesting that the high temporal sampling rate and positional accuracy of iSCAT data do not reduce the effective diffusion coefficient during confinement. In fact, the sampling timescale leads to frame-to-frame displacements that are on the order of the confinement radius, which required us to use OU dynamics in our MCMC algorithm. Failing to account for the effect of the frame-to-frame displacement/confinement size ratio on the integration scheme accuracy leads to a bias in the parameter estimates (data not shown).

This suggests that specifically modeling confinement, rather than modelling as a change in the diffusion coefficient, is necessary for data such as this. We believe the only other published confinement HMM for SPT analysis is by Bernstein et al. (31). Our models are similar, with both switching between free diffusion and confinement in a HPW. However, there are key differences. Firstly, we incorporate diffusion of the harmonic well to relax the constraint of a circular potential. The importance of this will depend on the spatial-temporal resolution of the data and whether the confinement zone is static or has time-dependent shape variation or drift. For instance, transient confinement in lipid rafts, which both diffuse and have an irregular shape, has been hypothesized. Secondly, the inference frameworks are different; we use a Bayesian approach to determine full posterior distributions for parameters and hidden states per trajectory, thus quantifying the level of uncertainty in these estimates. Bernstein et al. (31) use a maximal likelihood approach inferring a point estimate, using a particle filter within an expectation-maximization algorithm. Using a Bayesian analysis was essential in our study to show that significant intertrajectory heterogeneity was present. Such information was key to identifying the AuNP signatures and separating environment effects from movement characteristics specific to individual AuNPs. In absence of this information, we would only be able to pool the trajectory estimates to define the population statistics. Furthermore, Bernstein et al. (31) infer multiple, static confinement zones separately, as opposed to our single, moving confinement-zone solution. The merits of these distinct approaches to the problem of multiple confinement zones in different experimental applications is a rich area for future work. Finally, we comment that we have implemented a "hop diffusion" model for multiple adjacent confinement zones using reversible-jump MCMC (39), which may be more appropriate for some systems.

As with all analysis, model accuracy needs to be balanced against computational complexity. In practice, this balance is also affected by the data; data at higher spatial and temporal resolution allows more detailed models to be inferred because subtle model differences

can then be distinguished. We demonstrated our algorithm on high-resolution iSCAT data, with static localization error 2.7 nm, mean particle diffusivity 1.15 $\pm$ 0.106 $\mu m^2 s^{-1}$, and exposure time equal to 10^{-5} s (4). By subsampling the data at rate 10 (Figs. S1 and S2; Note S2 of the Supporting Materials and Methods), we mitigate any effects due to dynamic localization error. Under these conditions, confinement models and switching-diffusivity models can be distinguished. For other data types with lower localization accuracy and shorter trajectories, such as fluorescent probes, explicit consideration of measurement noise may be necessary. This is suggested by our simulations, in which we observed a bias in confinement strength κ as the measurement error increased beyond that of iSCAT data (Fig. S7). Our MCMC algorithm could be extended using an HMM with an unknown (hidden) particle position to model measurement noise as in (30); static and dynamic errors could also be separated (21,40).

The observation that individual AuNP/CTxB/GM1 complexes have a specific spatial signature means that distinguishing the effects of the tag from other factors, such as the cell membrane environment, is difficult. It follows that homogenous tags should improve characterization of the membrane environment. Because the variability in the tag signature presumably arises from the random placing of CTxB molecules on the AuNP surface, using particles with a structured surface is predicted to reduce or potentially eliminate this problem. Virions are ideal, given their highly geometric 3D structure. Interferometric label-free tracking of virions has been demonstrated at 3 s temporal resolution (41); thus, achieving the high spatial and temporal resolution of recent iSCAT (such as in the data set explored here) with viral particle tags is a distinct possibility. The resolution of the tag's signature and the length of events will then determine the resolution of that trajectory and thus the length scale to which SPT can discriminate different types of particle movement. Whether this can be taken below the size of the tag, reminiscent of super resolution, remains to be ascertained.

CONCLUSIONS

We use an HMM-based analysis to partition SPT trajectories into periods of free diffusion and confinement. Our algorithm infers the switching times between these two states, the diffusion coefficient D, and the characteristics of the following confinement events: the HPW strength κ, the position of the HPW center C, and the center diffusion coefficient D_C. We demonstrate the utility of the method on simulated and experimental data; on simulated data, confinement zones were accurately detected, and HPW centers accurately tracked while experimental trajectories were partitioned with high confidence. The model could potentially detect various biological phenomena such as lipid microdomains (or "rafts"), receptor clustering, and hop diffusion.

SUPPORTING MATERIAL

Supporting Materials and Methods, twenty figures, and two tables are available at http://www.biophysj.org/biophysj/supplemental/S0006-3495(18)31056-7.

AUTHOR CONTRIBUTIONS

Analyzed the data, P.J.S. and N.J.B.; Wrote the article, P.J.S. and N.J.B.; Developed the software, P.J.S.

ACKNOWLEDGMENTS

We thank Philipp Kukura for providing the data set for analysis and for helpful discussions and suggestions. We also thank Christian Eggeling and Gabrielle de Wit for helpful discussions and suggestions.

P.J.S. received a grant from the University of Warwick to study at Warwick Systems Biology Doctoral Training Centre funded by the Engineering and Physical Sciences Research Council and Biotechnology and Biological Sciences Research Council.

REFERENCES

1. Manzo, C., and M. F. Garcia-Parajo. A review of progress in single particle tracking: from methods to biophysical insights. *Rep. Prog. Phys.* 78:124601.
2. Shen, H., L. J. Tauzin, ..., C. F. Landes. Single particle tracking: from theory to biophysical applications. *Chem. Rev.* 117:7331–7376.
3. Ortega-Arroyo, J., and P. Kukura. Interferometric scattering microscopy (iSCAT): new frontiers in ultrafast and ultrasensitive optical microscopy. *Phys. Chem. Chem. Phys.* 15625.
4. Spillane, K. M., J. Ortega-Arroyo, ..., P. Kukura. 2014. High-speed single-particle tracking of GM1 in model membranes reveals anomalous diffusion due to interleaflet coupling and molecular pinning. *Nano Lett.* 14:5390–5397.
5. Hsieh, C. L., S. Spindler, ..., V. Sandoghdar. 2014. Tracking single particles on supported lipid membranes: multimobility diffusion and nanoscopic confinement. *J. Phys. Chem. B.* 118:1545–1554.
6. Lin, Y. H., W. L. Chang, and C. L. Hsieh. 2014. Shot-noise limited localization of single 20 nm gold particles with nanometer spatial precision within microseconds. *Opt. Express.* 22:9159–9170.
7. Clausen, M. P., and B. C. Lagerholm. 2011. The probe rules in single particle tracking. *Curr. Protein Pept. Sci.* 12:699–713.
8. Qian, H., M. P. Sheetz, and E. L. Elson. 1991. Single particle tracking. Analysis of diffusion and flow in two-dimensional systems. *Biophys. J.* 60:910–921.
9. Michalet, X. 2010. Mean square displacement analysis of single-particle trajectories with localization error: Brownian motion in an isotropic medium. *Phys. Rev. E Stat. Nonlin. Soft Matter Phys.* 82:041914.
10. Michalet, X., and A. J. Berglund. 2012. Optimal diffusion coefficient estimation in single-particle tracking. *Phys. Rev. E Stat. Nonlin. Soft Matter Phys.* 85:061916.
11. Monnier, N., S. M. Guo, ..., M. Bathe. 2012. Bayesian approach to MSD-based analysis of particle motion in live cells. *Biophys. J.* 103:616–626.
12. Clausen, M. P., and B. C. Lagerholm. 2013. Visualization of plasma membrane compartmentalization by high-speed quantum dot tracking. *Nano Lett.* 13:2332–2337.
13. Lagerholm, B. C., D. M. Andrade, ..., C. Eggeling. 2017. Convergence of lateral dynamic measurements in the plasma membrane of live cells

from single particle tracking and STED-FCS. *J. Phys. D Appl. Phys.* 50:063001–063020.

14. Saxton, M. J. 1993. Lateral diffusion in an archipelago. Single-particle diffusion. *Biophys. J.* 64:1766–1780.
15. Simson, R., E. D. Sheets, and K. Jacobson. 1995. Detection of temporary lateral confinement of membrane proteins using single-particle tracking analysis. *Biophys. J.* 69:989–993.
16. Meilhac, N., L. Le Guyader, …, N. Destainville. 2006. Detection of confinement and jumps in single-molecule membrane trajectories. *Phys. Rev. E Stat. Nonlin. Soft Matter Phys.* 73:011915.
17. Rajani, V., G. Carrero, …, C. W. Cairo. 2011. Analysis of molecular diffusion by first-passage time variance identifies the size of confinement zones. *Biophys. J.* 100:1463–1472.
18. Coppola, S., G. Caracciolo, and T. Schmidt. 2014. Exact occupation probabilities for intermittent transport and application to image correlation spectroscopy. *New J. Phys.* 16:113057.
19. Renner, M., L. Wang, …, A. Triller. A simple and powerful analysis of lateral subdiffusion using single particle tracking. *Biophys. J.* 113:2452–2463.
20. Koo, P. K., M. Weitzman, …, S. G. Mochrie. Extracting diffusive states of Rho GTPase in live cells: towards in vivo biochemistry. *PLoS Comput. Biol*. 11:e1004297.
21. Calderon, C. P., and K. Bloom. Inferring latent states and refining force estimates via hierarchical dirichlet process modeling in single particle tracking experiments. *PLoS One*. 10:e0137633.
22. Koo, P. K., and S. G. Mochrie. Systems-level approach to uncovering diffusive states and their transitions from single-particle trajectories. *Phys. Rev. E Stat. Nonlin. Soft Matter Phys*. 94:052412.
23. Wagner, T., A. Kroll, …, M. Wiemann. Classification and segmentation of nanoparticle diffusion trajectories in cellular micro environments. *PLoS One*. 12:e0170165.
24. Yin, S., N. Song, and H. Yang. Detection of velocity and diffusion coefficient change points in single-particle trajectories. *Biophys. J.* 115:217–229.
25. Das, R., C. W. Cairo, and D. Coombs. 2009. A hidden Markov model for single particle tracks quantifies dynamic interactions between LFA-1 and the actin cytoskeleton. *PLoS Comput. Biol.* 5:e1000556.
26. Chung, I., R. Akita, …, I. Mellman. 2010. Spatial control of EGF receptor activation by reversible dimerization on living cells. *Nature.* 464:783–787.
27. Ott, M., Y. Shai, and G. Haran. 2013. Single-particle tracking reveals switching of the HIV fusion peptide between two diffusive modes in membranes. *J. Phys. Chem. B.* 117:13308–13321.
28. Persson, F., M. Lindén, …, J. Elf. 2013. Extracting intracellular diffusive states and transition rates from single-molecule tracking data. *Nat. Methods.* 10:265–269.
29. Monnier, N., Z. Barry, …, M. Bathe. 2015. Inferring transient particle transport dynamics in live cells. *Nat. Methods.* 12:838–840.
30. Slator, P. J., C. W. Cairo, and N. J. Burroughs. 2015. Detection of diffusion heterogeneity in single particle tracking trajectories using a hidden Markov model with measurement noise propagation. *PLoS One.* 10:e0140759.
31. Bernstein, J., and J. Fricks. 2016. Analysis of single particle diffusion with transient binding using particle filtering. *J. Theor. Biol.* 401:109–121.
32. Fox, E., E. B. Sudderth, …, A. S. Willsky. 2011. Bayesian nonparametric inference of switching dynamic linear models. *IEEE Trans. Signal Process.* 59:1569–1585.
33. Alvarez, I., J. Niemi, and M. Simpson. 2014. Bayesian inference for a covariance matrix. *Ann. Conf. on Applied Statistics in Agriculture.* 26:71–82.
34. Gelman, A., J. B. Carlin, …, D. B. Rubin. 2013. Bayesian Data Analysis, Third Edition. CRC Press, Boca Raton, FL.
35. Tarantino, N., J. Y. Tinevez, …, E. Laplantine. 2014. TNF and IL-1 exhibit distinct ubiquitin requirements for inducing NEMO-IKK supramolecular structures. *J. Cell Biol.* 204:231–245.
36. Churchman, L. S., H. Flyvbjerg, and J. A. Spudich. 2006. A non-Gaussian distribution quantifies distances measured with fluorescence localization techniques. *Biophys. J.* 90:668–671.
37. Margheri, G., R. D'Agostino, …, M. Del Rosso. 2014. The β-subunit of cholera toxin has a high affinity for ganglioside GM1 embedded into solid supported lipid membranes with a lipid raft-like composition. *Lipids.* 49:203–206.
38. Shi, J., T. Yang, …, P. S. Cremer. 2007. GM1 clustering inhibits cholera toxin binding in supported phospholipid membranes. *J. Am. Chem. Soc.* 129:5954–5961.
39. Slator, P. J. 2015. Characterising cell membrane heterogeneity through analysis of particle trajectories. Ph.D. thesis (University of Warwick).
40. Berglund, A. J. 2010. Statistics of camera-based single-particle tracking. *Phys. Rev. E Stat. Nonlin. Soft Matter Phys.* 82:011917.
41. Ewers, H., V. Jacobsen, …, V. Sandoghdar. 2007. Label-free optical detection and tracking of single virions bound to their receptors in supported membrane bilayers. *Nano Lett.* 7:2263–2266.
42. Arthur, D., and S. Vassilvitskii. 2007. K-Means++: the advantages of careful seeding. Proceedings of the 18th Annual ACM-SIAM Symposium on Discrete algorithms. 8:1027–1025.

Biophysical Journal, Volume *115*

Supplemental Information

A Hidden Markov Model for Detecting Confinement in Single-Particle Tracking Trajectories

Paddy J. Slator and Nigel J. Burroughs

Supporting Material: A Hidden Markov Model for Detecting Confinement in Single Particle Tracking Trajectories

PJ Slator, NJ Burroughs

1 Note S1: MCMC algorithm for harmonic potential confinement HMM

We implemented the following MCMC algorithm in Matlab. The source code, documentation, trajectory data, and working examples are freely available (doi:10.5281/zenodo.1405647).

For a 2D trajectory $\mathbf{X} = \{X_i, t_i\}_{i=1}^{N+1}$ the switching harmonic confinement model with parameters $\{\kappa, D, D_C\}$ and hidden states $\{\mathbf{z}, \mathbf{C}\}$ has dynamics

$$\begin{aligned} dX_t &= -\kappa z_t (X_t - C_t) dt + \sqrt{2D} dW_t \\ dC_t &= \sqrt{2\big(D_C z_t + D_{est}(1 - z_t)\big)} dW_t^{(C)}. \end{aligned} \tag{1}$$

Where (as in the main text) $X_i = (X_i^{(1)}, X_i^{(2)})$ and $C_i = (C_i^{(1)}, C_i^{(2)})$ are 2D vectors. During confinement X_t has Ornstein-Uhlenbeck dynamics with centre C_t. This model assumes that switching can only occur at sampling points and that measurement noise is negligible. Measurement noise can be incorporated by introducing a model for the measured position Y_t dependent on the true position X_t, e.g. a Gaussian variate. This would allow complex measurement noise, such as raster scan anisotropic measurement errors, to be incorporated. However, the inference algorithm would be substantially more complex because of the additional hidden Markov chain X_t, so is not pursued here.

To integrate over a time step Δt when confined ($z_t = 1$), we can use the OU solution in X_t (assuming C_t is constant over a time step). Thus, ignoring time dependence of C_t over Δt we get,

$$X_{t+\Delta t}\Big|_{X_t, z_t=1} \sim N\left(C_t + (X_t - C_t)e^{-\kappa \Delta t}, \frac{D}{\kappa}\left(1 - e^{-2\kappa \Delta t}\right)\right) \tag{2}$$

and $C_{t+\Delta t} \sim N(C_t, 2D_C \Delta t)$, where $N(x; \mu, \sigma)$ is the normal PDF with mean μ and variance σ, (we use this parameterisation and notation throughout). If $\Delta t \to 0$ we obtain the simplification $X_{t+\Delta t}\Big|_{X_t, z_t=1} \sim N\left(X_t - \kappa \Delta t (X_t - C_t), 2D\Delta t\right)$ as expected under Euler-Maruyama integration of the SDE. The latter is valid provided $\kappa \Delta t << 1$, or $D\Delta t << D/\kappa$, i.e. the diffusional displacement over Δt is substantially smaller than the confinement width of the Harmonic potential well.

Using this solution (Equation 2) we can state the following: for a 2D trajectory $\mathbf{X} = \{X_i, t_i\}_{i=1}^{N+1}$ the probability of observing $\mathbf{X}$ given the parameters (θ) and hidden states ($\mathbf{z}$, $\mathbf{C}$) is (recall that $\Delta X_i = X_{i+1} - X_i, \Delta t_i = t_{i+1} - t_i$)

$$\begin{aligned} \pi(\mathbf{X}|\theta, \mathbf{z}, \mathbf{C}) &= \prod_{i=1|z_i=0}^{N} N(\Delta X_i; 0, 2D\Delta t_i) \prod_{i=1|z_i=1}^{N} N\left(X_{i+1}; C_i + (X_i - C_i)e^{-\kappa \Delta t_i}, \frac{D}{\kappa}\left(1 - e^{-2\kappa \Delta t_i}\right)\right) \\ &= \prod_{i=1}^{N} N\left(\Delta X_i; z_i\left((C_i - X_i)\left(1 - e^{-\kappa \Delta t_i}\right)\right), D\left((1 - z_i)2\Delta t_i + \frac{z_i}{\kappa}\left(1 - e^{-2\kappa \Delta t_i}\right)\right)\right). \end{aligned} \tag{3}$$

Since ΔX_i is 2D this can be rewritten as a product over each coordinate. Using Bayes' rule we can write the posterior ($\Delta C_i = C_{i+1} - C_i$)

$$\pi(\theta, \mathbf{z}, \mathbf{C}|\mathbf{X}) \propto \pi(\theta, z_1, C_1) \prod_{i=1}^{N-1} \text{Bernoulli}(z_{i+1}; z_i(1-p_{esc}) + (1-z_i)p_{trap})$$
$$\times \prod_{i=1}^{N} N\left(\Delta X_i; z_i\left(C_i - X_i + (X_i - C_i)e^{-\kappa\Delta t_i}\right), D\left((1-z_i)2\Delta t_i + \frac{z_i}{\kappa}\left(1 - e^{-2\kappa\Delta t_i}\right)\right)\right)$$
$$\times \prod_{i=1}^{N-1} N\left(\Delta C_i; 0, 2\Delta t_i(D_C z_i + D_{est}(1-z_i))\right), \tag{4}$$

where $\pi(\theta, z_1, C_1)$ is the prior. We use conjugate priors for all parameters, specifically

$$\pi(\theta, z_1, C_1) = \text{Unif}(D; D_{min}, D_{max})\text{Unif}(D_C; D_{C_{min}}, D_{max}/D_{ratio})\text{Unif}(\kappa; \kappa_{min}, \kappa_{max})$$
$$\times \text{Beta}(p_{esc}; a_{esc}, b_{esc})\text{Beta}(p_{trap}; a_{trap}, b_{trap})$$
$$\times \text{Bernoulli}(z_1; \pi_{trap})N(C_1; \mu_{C_1}, 1/\tau_{C_1}). \tag{5}$$

We choose prior parameters which enforce model conditions, for example a large D_{ratio} implies $D >> D_C$.

1.1 MCMC sampler

We developed an MCMC sampler which draws samples from this distribution as follows.

Parameter updates A Gibbs sampler can be used (under conjugate priors) for conditional posterior sampling of the diffusion coefficients (D and D_C) and transition probabilities (p_{esc}, p_{trap}). Specifically, the updates for D and D_C are inverse-Gamma

$$D^{-1}|_{D_C,\kappa,p_{esc},p_{trap},\mathbf{X},\mathbf{C},\mathbf{z}} \sim \text{Gamma}_T\left(N-1, \frac{1}{2}\sum_{i=1}^{N} \frac{\left(\Delta X_i - z_i\left(C_{t_i} - X_i + (X_{t_i} - C_{t_i})e^{-\kappa\Delta t_i}\right)\right)^2}{(1-z_i)2\Delta t_i + \frac{z_i}{\kappa}\left(1 - e^{-2\kappa\Delta t_i}\right)}, D_{min}, D_{max}\right) \tag{6}$$

$$D_C^{-1}|_{D,\kappa,p_{esc},p_{trap},\mathbf{X},\mathbf{C},\mathbf{z}} \sim \text{Gamma}_T\left(1 + \sum_{i|z_i=1} 1, \frac{1}{4}\sum_{i|z_i=1} \frac{\Delta C_i^2}{\Delta t_i}, D_{C_{min}}, D_{max}/D_{ratio}.\right) \tag{7}$$

where $\text{Gamma}_T(\alpha, \beta, x_{min}, x_{max})$ denotes a truncated Gamma distribution with parameters α and β, truncated at x_{min} and x_{max}. We enforce the truncation by rejecting any moves which lie outside this region. If $\sum_{i|z_i=1} 1 = 0$ then the conditional for D_C reduces to the prior, so we update by sampling from $\text{Unif}(D_{C_{min}}, D_{max}/D_{ratio})$.

The updates for the transition probabilities are from Beta distributions

$$p_{esc}|_{D,D_C,\kappa,p_{trap},\mathbf{X},\mathbf{z},\mathbf{C}} \sim \text{Beta}\left(a_{esc} + n_{10}, b_{esc} + n_{11}\right) \tag{8}$$

$$p_{trap}|_{D,D_C,\kappa,p_{esc},\mathbf{X},\mathbf{z},\mathbf{C}} \sim \text{Beta}\left(a_{trap} + n_{01}, b_{trap} + n_{00}\right) \tag{9}$$

where n_{ml} is the number of transitions from state m to state l, i.e.

$$n_{ml} = \sum_{i|z_{i-1}=m, z_i=l} 1. \tag{10}$$

The harmonic potential well strength (κ) is updated with a Metropolis-Hastings step. The conditional distribution is

$$\pi(\kappa|D, D_C, p_{esc}, p_{trap}, \mathbf{X}, \mathbf{C}, \mathbf{z}) \propto \prod_{z_i=1} N\left(\Delta X_i; (C_{t_i} - X_{t_i})\left(1 - e^{-\kappa\Delta t_i}\right), \frac{D}{\kappa}\left(1 - e^{-2\kappa\Delta t_i}\right)\right) \tag{11}$$

We use a Metropolis-Hastings move with Gaussian proposal

$$\kappa' \sim N(\kappa, s_\kappa) \tag{12}$$

where s_κ was chosen empirically to give an acceptance rate of approximately 0.3. The acceptance probability is

$$\alpha(\kappa'|\kappa) = \frac{\pi(\kappa'|D, D_C, p_{esc}, p_{trap}, \mathbf{X}, \mathbf{C}, \mathbf{z})}{\pi(\kappa|D, D_C, p_{esc}, p_{trap}, \mathbf{X}, \mathbf{C}, \mathbf{z})}. \tag{13}$$

We enforce the prior, Unif$(\kappa; \kappa_{min}, \kappa_{max})$, by rejecting any values of κ lying outside this interval.

Gibbs update for the Harmonic Potential well centre. For the centre, $\mathbf{C}$, we update using a blocked Gibbs move. We report the update here without calculation; the full derivation is given in section “Full derivation of Gibbs move for harmonic well centre”. Let $\mathbf{C}_{j,n}$ be a block of length n starting at j, i.e. $\mathbf{C}_{j,n} = \{C_i\}_{i=j}^{j+n}$. The update is

$$\mathbf{C}_{j,n} \sim N(\boldsymbol{\mu}_{C_{j,n}}, \boldsymbol{\Sigma}^{-1}_{C_{j,n}}) \tag{14}$$

where the $n \times n$ precision matrix is

$$\boldsymbol{\Sigma}^{-1}_{C_{j,n}} = \begin{pmatrix} \Sigma^{-1}_{j,j} & \Sigma^{-1}_{j,j+1} & & & & \\ \Sigma^{-1}_{j+1,j} & \Sigma^{-1}_{j+1,j+1} & \Sigma^{-1}_{j+1,j+2} & & & \\ & \Sigma^{-1}_{j+2,j+1} & \ddots & & & \\ & & & \ddots & & \\ & & & & \ddots & \Sigma^{-1}_{j+n-1,j+n} \\ & & & & \Sigma^{-1}_{j+n,j+n-1} & \Sigma^{-1}_{j+n,j+n} \end{pmatrix} \tag{15}$$

with elements

$$\Sigma^{-1}_{i,i-1} = \Sigma^{-1}_{i-1,i} = \frac{-1}{2\Delta t_i(D_{est}(1-z_i) + D_C z_i)} \tag{16}$$

$$\Sigma^{-1}_{i,i}\big|_{2\le i\le N-1} = \frac{z_i(1-e^{\kappa\Delta t_i})^2}{\frac{D}{\kappa}(1-e^{-2\kappa\Delta t_i})} + \frac{1}{2\Delta t_{i-1}(D_C z_{i-1} + D_{est}(1-z_{i-1}))} + \frac{1}{2\Delta t_i(D_C z_i + D_{est}(1-z_i))}. \tag{17}$$

If the block contains the first timepoint, i.e. $j = 1$, then the first element is

$$\Sigma^{-1}_{1,1} = \frac{z_1(1-e^{\kappa\Delta t_1})^2}{\frac{D}{\kappa}(1-e^{-2\kappa\Delta t_1})} + \tau_{C_1} + \frac{1}{2\Delta t_1(D_C z_1 + D_{est}(1-z_1))} \tag{18}$$

and if the block contains the last timepoint, i.e. $j + n = N$, then the last element is

$$\Sigma^{-1}_{N,N} = \frac{z_N(1-e^{\kappa\Delta t_N})^2}{\frac{D}{\kappa}(1-e^{-2\kappa\Delta t_N})} + \frac{1}{2\Delta t_{N-1}(D_C z_{N-1} + D_{est}(1-z_{N-1}))}. \tag{19}$$

The mean $\boldsymbol{\mu}_{C_{j,n}}$ is given by solving

$$\boldsymbol{\Sigma}^{-1}_{C_{j,n}}\boldsymbol{\mu}_{C_{j,n}} = \mathbf{b}_{j,n} \tag{20}$$

where $\mathbf{b}_{j,n} = \{b_i\}_{i=j}^{j+n}$ is an n-vector with elements

$$b_i\bigg|_{j+1\le i\le j+n-1} = \frac{z_i(1-e^{-\kappa\Delta t_i})^2)}{\frac{D}{\kappa}(1-e^{-2\kappa\Delta t_i})}\frac{\Delta X_i + X_i(1-e^{-\kappa\Delta t_i})}{1-e^{-\kappa\Delta t_i}} \tag{21}$$

$$b_j\bigg|_{j\ne 1} = \frac{C_{j-1}}{2\Delta t_{j-1}(D_C z_{j-1} + D_{est}(1-z_{j-1}))} + \frac{z_i(1-e^{-\kappa\Delta t_i})^2)}{\frac{D}{\kappa}(1-e^{-2\kappa\Delta t_i})}\frac{\Delta X_i + X_i(1-e^{-\kappa\Delta t_i})}{1-e^{-\kappa\Delta t_i}} \tag{22}$$

$$b_{j+n}\bigg|_{j+n\ne N} = \frac{C_{j+n+1}}{2\Delta t_{j+n}(D_C z_{j+n} + D_{est}(1-z_{j+n}))} + \frac{z_i(1-e^{-\kappa\Delta t_i})^2)}{\frac{D}{\kappa}(1-e^{-2\kappa\Delta t_i})}\frac{\Delta X_i + X_i(1-e^{-\kappa\Delta t_i})}{1-e^{-\kappa\Delta t_i}}. \tag{23}$$

If the block contains the first timepoint then

$$b_1 = \tau_{C_1}\mu_{C_1} + \frac{z_i(1-e^{-\kappa\Delta t_i})^2)}{\frac{D}{\kappa}(1-e^{-2\kappa\Delta t_i})}\frac{\Delta X_i + X_i(1-e^{-\kappa\Delta t_i})}{1-e^{-\kappa\Delta t_i}} \tag{24}$$

and if the block contains the last timepoint,

$$b_N = \frac{z_i(1-e^{-\kappa\Delta t_i})^2)}{\frac{D}{\kappa}(1-e^{-2\kappa\Delta t_i})}\frac{\Delta X_i + X_i(1-e^{-\kappa\Delta t_i})}{1-e^{-\kappa\Delta t_i}}. \tag{25}$$

Since $\boldsymbol{\Sigma}^{-1}_{C_{j,n}}$ is tridiagonal, Equation (20) can be efficiently solved for $\boldsymbol{\mu}_{C_{j,n}}$, for example using the left matrix division function in Matlab (with $\boldsymbol{\Sigma}^{-1}_{C_{j,n}}$ encoded as a sparse matrix). Hence the update can be sampled from Equation (14).

Metropolis-Hastings move for $\mathbf{z}$ *and* $\mathbf{C}$. Since the hidden states in the model are highly correlated, we developed a joint update for $\mathbf{z}$ and $\mathbf{C}$, a blocked Metropolis-Hastings move. To simulate values from $\pi(\mathbf{z}, \mathbf{C}|\theta, \mathbf{X})$, we first propose new values of $\{\mathbf{z}, \mathbf{C}\}$ for a block of length n, let $\{\mathbf{z}_{j,n}, \mathbf{C}_{j,n}\} = \{z_i, C_i\}_{i=j}^{j+n}$ denote this block. We use a joint proposal distribution

$$q(\mathbf{z}'_{j,n}, \mathbf{C}'_{j,n}|\mathbf{z}_{j,n}) = q(\mathbf{z}'_{j,n}|\mathbf{z}_{j,n})q(\mathbf{C}'_{j,n}|\mathbf{z}'_{j,n}). \tag{26}$$

For $\mathbf{z}_{j,n}$ we use a proposal distribution $q(\mathbf{z}'_{j,n}|\mathbf{z}_{j,n})$ which is weighted towards complete confinement or free diffusion. Specifically, let 0^n and 1^n denote a sequence of 0's or 1's of length n, we propose $\mathbf{z}'_{j,n} = 0^n$ and $\mathbf{z}'_{j,n} = 1^n$ both with probability 1/3. Otherwise, with probability 1/3, we propose a random sequence generated from by the Markov chain with parameters p_{esc}, p_{trap}. The proposal density is hence

$$q(\mathbf{z}'_{j,n}|\mathbf{z}_{j,n})\Big|_{j\neq 1} = $$

$$\begin{cases} \frac{1}{3} + \frac{1}{3}\prod_{i=j}^{n+j+1} \text{Bernoulli}\left(z_i; z_{i-1}(1-p_{esc}) + (1-z_{i-1})p_{trap}\right) & \text{if } \mathbf{z}'_{j,n} = 0^n \\ \frac{1}{3} + \frac{1}{3}\prod_{i=j}^{n+j+1} \text{Bernoulli}\left(z_i; z_{i-1}(1-p_{esc}) + (1-z_{i-1})p_{trap}\right) & \text{if } \mathbf{z}'_{j,n} = 1^n \\ \frac{1}{3}\prod_{i=j}^{n+j+1} \text{Bernoulli}\left(z_i; z_{i-1}(1-p_{esc}) + (1-z_{i-1})p_{trap}\right) & \text{any other } \mathbf{z}'_{j,n}. \end{cases} \tag{27}$$

If $j = 1$, then the first term in the product in Equation (27) is undefined, so we propose z_1 from the prior distribution, Bernoulli$(z_1; p_{trap}/(p_{trap} + p_{esc}))$, giving

$$q(\mathbf{z}'_{1,n}|\mathbf{z}_{1,n}) = \begin{cases} \frac{1}{3} + \frac{1}{3}\left(1 - \frac{p_{trap}}{p_{trap}+p_{esc}}\right) \\ \quad \times \prod_{i=2}^{n+2} \text{Bernoulli}\left(z_i; z_{i-1}(1-p_{esc}) + (1-z_{i-1})p_{trap}\right) & \text{if } \mathbf{z}'_{1,n} = 0^n \\ \frac{1}{3} + \frac{1}{3}\frac{p_{trap}}{p_{trap}+p_{esc}} \\ \quad \times \prod_{i=2}^{n+2} \text{Bernoulli}\left(z_i; z_{i-1}(1-p_{esc}) + (1-z_{i-1})p_{trap}\right) & \text{if } \mathbf{z}'_{1,n} = 1^n \\ \frac{1}{3}\text{Bernoulli}\left(z_1; \frac{p_{trap}}{p_{trap}+p_{esc}}\right) \\ \quad \times \prod_{i=2}^{n+2} \text{Bernoulli}\left(z_i; z_{i-1}(1-p_{esc}) + (1-z_{i-1})p_{trap}\right) & \text{any other } \mathbf{z}'_{1,n}. \end{cases} \tag{28}$$

The right hand terms in the top two cases of Equations (27) and (28) are required because the sequences 0^n and 1^n are possible when simulating a Markov chain with parameters p_{esc} and p_{trap}.

We next propose a value for $\mathbf{C}_{j,n}$ using the block Gibbs update density derived earlier (Equations (14)-(25))

$$q(\mathbf{C}'_{j,n}|\mathbf{z}'_{j,n}) = N(\mathbf{C}'_{j,n}; \boldsymbol{\mu}_{C_{j,n}}, \boldsymbol{\Sigma}^{-1}_{C_{j,n}}) \tag{29}$$

where $\boldsymbol{\mu}_{C_{j,n}}$, $\boldsymbol{\Sigma}^{-1}_{C_{j,n}}$ are calculated (using Equations (15)-(20)) with the proposed value $\mathbf{z}'_{j,n}$. Up to proportionality, the density of interest is

$$\pi(\mathbf{z}, \mathbf{C}|\theta, \mathbf{X}) \propto \prod_{i=1}^{N} N\left(\Delta X_i; z_i\left(C_i - X_i\right)\left(1 - e^{-\kappa \Delta t_i}\right), D\left((1-z_i)2\Delta t_i + \frac{z_i}{\kappa}\left(1 - e^{-2\kappa\Delta t_i}\right)\right)\right)$$

$$\times N(C_1, \mu_{C_1}, 1/\tau_{C_1}) \prod_{i=1}^{N-1} N\left(\Delta C_i; 0, 2\Delta t_i(D_C z_i + D_{est}(1 - z_i))\right) \quad (30)$$

$$\times \text{Bernoulli}\left(z_1; \frac{p_{trap}}{p_{trap} + p_{esc}}\right) \prod_{i=1}^{N-1} \text{Bernoulli}(z_{i+1}; z_i(1 - p_{esc}) + (1 - z_i)p_{trap}). \quad (31)$$

which we call $P(\mathbf{z}, \mathbf{C})$. The acceptance probability is thus

$$\alpha(\mathbf{z}', \mathbf{C}'|\mathbf{z}, \mathbf{C}) = \min\left\{1, \frac{P(\mathbf{z}', \mathbf{C}')q(\mathbf{z}_{j,n}|\mathbf{z}'_{j,n})q(\mathbf{C}_{j,n}|\mathbf{z}_{j,n})}{P(\mathbf{z}, \mathbf{C})q(\mathbf{z}'_{j,n}|\mathbf{z}_{j,n})q(\mathbf{C}'_{j,n}|\mathbf{z}'_{j,n})}\right\}. \quad (32)$$

By sequentially calculating these MCMC updates, we can sample from the posterior distribution $\pi(\theta, \mathbf{z}, \mathbf{C}|\mathbf{X})$. Algorithm 1 (in Supporting Material) details this HPW model MCMC algorithm in pseudocode.

Blocking choice. The two blocked moves require a choice of block size n and starting point j. For the blocked Gibbs move for $\mathbf{C}$ we found that updating the whole Markov chain at once, i.e. $j = 1$ and $n = N - 1$ was most efficient. For the blocked Metropolis-Hastings move for $\{\mathbf{z}, \mathbf{C}\}$ we sample the block size n from Unif(B_{min}, B_{max}), and then sample j from Unif$(1, N - n)$. For all MCMC runs on real data we set $B_{min} = 2$ and $B_{max} = 200$. For MCMC runs on simulated data we heuristically tuned.

Algorithm runtime and convergence. The main factors which affect the algorithm runtime are the trajectory length and hidden state update blocking choices, detailed above. The main bottlenecks in the blocked hidden state updates are the calculation of $\mu_{C_{j,n}}$ and sampling from Equation (29). These require solving Equation (20) and Cholsky decomposition of $\Sigma^{-1}_{C_{j,n}}$ (note that inversion of $\Sigma^{-1}_{C_{j,n}}$ is not necessary) respectively. Clearly, the larger the blocks, the longer the runtime, although we were able to obtain speed ups by using sparse matrices in Matlab. However, runtime is not the same as MCMC algorithm convergence - which is affected by many factors and will vary depending on the data; the complexity of the confinement state sequence strongly affects the convergence rate as the algorithm needs to "find" the correct confinement sequence through hidden state updates. We advise trying different hidden state update blocking choices on new data sets to determine acceptable chain mixing and runtimes.

Algorithm 1 Metropolis-within-Gibbs sampler for harmonic potential well model

$K \leftarrow$ number of MCMC steps
$D_{min}, D_{C_{min}}, D_{max}, D_{ratio}, \kappa_{min}, \kappa_{max}, a_{esc}, b_{esc}, a_{trap}, b_{trap} \leftarrow$ prior parameters
$B_{max}, B_{min} \leftarrow$ maximum and minimum Metropolis-Hastings block sizes
$D_{est} \leftarrow$ estimated value for D (Equation (34))
Choose initial values - $D^{(0)}, D_C^{(0)}, \kappa^{(0)}, p_{esc}^{(0)}, p_{trap}^{(0)}, z^{(0)}$ (e.g. sample from Equation (5))
Calculate $\boldsymbol{\mu}_{C_{1,N-1}}$ (mean vector) and $\boldsymbol{\Sigma}^{-1}_{C_{1,N-1}}$ (precision matrix) using Equations (15)-(20)
$\mathbf{C}^{(0)} \leftarrow$ random number drawn from $N(\boldsymbol{\mu}_{C_{1,N-1}}, \boldsymbol{\Sigma}^{-1}_{C_{1,N-1}})$
for $k = 1$ to $k = K$ **do**
  $1/D^{(k)} \leftarrow$ random number drawn from $\Gamma\left(N-1, \frac{1}{2}\sum_{i=1}^{N} \frac{(\Delta X_i - z_i\left(C_{t_i} - X_i + (X_{t_i} - C_{t_i})e^{-\kappa \Delta t_i}\right)^2}{(1-z_i)2\Delta t_i + \frac{z_i}{\kappa}\left(1-e^{-2\kappa\Delta t_i}\right)}\right)$
  if $D < D_{min}$ **or** $D > D_{max}$ **then**
    $D^{(k)} \leftarrow D^{(k-1)}$
  end if
  if $\sum_{i|z_i=1} 1 = 0$ **then**
    $1/D_C^{(k)} \leftarrow$ random number drawn from Unif$(D_{C_{min}}, D_{max}/D_{ratio})$
  else
    $1/D_C^{(k)} \leftarrow$ random number drawn from $\Gamma\left(1 + \sum_{i|z_i=1} 1, \frac{1}{4}\sum_{i|z_i=1} \frac{\Delta C_i^2}{\Delta t_i}\right)$
  end if
  if $D_C < D_{C_{min}}$ **or** $D_C > D_{max}/D_{ratio}$ **then**
    $D_C^{(k)} \leftarrow D_C^{(k-1)}$
  end if
  $\kappa' \leftarrow$ random number drawn from $N(\kappa^{(k-1)}, s_\kappa)$
  if $\kappa' > \kappa_{min}$ **and** $\kappa' < \kappa_{max}$ **then**
    Calculate $\alpha(\kappa'|\kappa)$ using Equation (13)
    $u \leftarrow$ random number drawn from Unif$(0,1)$
    if $u < \alpha(\kappa'|\kappa)$ **then**
      $\kappa^{(k)} \leftarrow \kappa'$
    end if
  end if
  Calculate $n_{00}, n_{01}, n_{10}, n_{11}$ using Equation (10)
  $p_{esc}^{(k)} \leftarrow$ random number drawn from Beta$\left(a_{esc} + n_{10}, b_{esc} + n_{11}\right)$
  $p_{trap}^{(k)} \leftarrow$ random number drawn from Beta$\left(a_{trap} + n_{01}, b_{trap} + n_{00}\right)$
  $n \leftarrow$ random integer drawn from $\{B_{min}, B_{min}+1, ..., B_{max}\}$
  $j \leftarrow$ random integer drawn from $\{1, ..., N-n\}$
  Calculate $\boldsymbol{\mu}_{C_{j,n}}$ (mean vector) and $\boldsymbol{\Sigma}^{-1}_{C_{j,n}}$ (precision matrix) using Equations (15)-(20)
  $\mathbf{C} \leftarrow$ random number drawn from $N(\boldsymbol{\mu}_{C_{1,N-1}}, \boldsymbol{\Sigma}^{-1}_{C_{1,N-1}})$
  $n \leftarrow$ random integer drawn from $\{B_{min}, B_{min}+1, ..., B_{max}\}$
  $j \leftarrow$ random integer drawn from $\{1, ..., N-n\}$
  $\mathbf{z}' \leftarrow$ proposed value drawn from $q(\mathbf{z})$ (Equation (27))
  Calculate $\boldsymbol{\mu}_{C_{j,n}}$ and $\boldsymbol{\Sigma}^{-1}_{C_{j,n}}$ (given $\mathbf{z}'$) using Equations (15)-(20)
  $\mathbf{C}'_{j,n} \leftarrow$ random number drawn from $N(\boldsymbol{\mu}_{C_{j,n}}, \boldsymbol{\Sigma}^{-1}_{C_{j,n}})$
  calculate $\alpha(\mathbf{z}', \mathbf{C}'|\mathbf{z}, \mathbf{C})$ using Equation (32)
  $u \leftarrow$ random number drawn from Unif$(0,1)$
  if $u < \alpha(\mathbf{z}', \mathbf{C}'|\mathbf{z}, \mathbf{C})$ **then**
    $\mathbf{z} \leftarrow \mathbf{z}'$
    $\mathbf{C} \leftarrow \mathbf{C}'$
  end if
  $\mathbf{z}^{(k)} \leftarrow \mathbf{z}$
  $\mathbf{C}^{(k)} \leftarrow \mathbf{C}$
end for

Initial values and priors. For all MCMC runs on simulated and experimental data the priors were as follows

$$\begin{aligned}
\pi(D) &= \text{Unif}(D; 0, 4\,\mu\text{m}^2\,\text{s}^{-1}) \\
\pi(D_C) &= \text{Unif}(D_C; 0, 0.04\,\mu\text{m}^2\,\text{s}^{-1}) \\
\pi(\kappa) &= \text{Unif}(\kappa; 0\,\text{s}^{-1}, 20\,000\,\text{s}^{-1}) \\
\pi(p_{esc}) &= \text{Beta}(p_{esc}; 1, 1000) \\
\pi(p_{trap}) &= \text{Beta}(p_{trap}; 1, 1000).
\end{aligned} \tag{33}$$

We chose non-zero $D_{C_{min}}$ because very low D_C values occasionally caused computational overflow in the blocked update covariance matrix $\mathbf{\Sigma}_{C_{k,n}}$. We chose non-zero κ_{min} because MCMC chains were occasionally very slow to converge from very low values of κ. Inferred posterior distributions for both these parameters were much higher than these minimum values. We chose an informative prior, Beta$(1, 1000)$, on the transition probabilities to inhibit rapid switching and short confinement events. These events are also inhibited by the implicit prior penalty against a transition to a confinement state, due to the diffusion of the confinement centre C_t when the particle X_t is not confined. The probability of C_t and X_t being in close proximity is proportional to $\frac{1}{(D_{est}+D)t}$, where t is the time since the end of the last confinement event. This prior dependence is weak and no issues were detected due to it, see Fig. S5. We initialised D, D_C, κ, p_{esc} and p_{trap} by sampling from the prior distributions. For D_{est} (the diffusion coefficient of C when X is confined) we used the Brownian motion maximum likelihood estimate

$$D_{est} = \frac{1}{4N} \sum_{i=1}^{N} \frac{\Delta X_i^2}{\Delta t_i}. \tag{34}$$

For $\mathbf{z}$ we initialised by simulating a Markov chain using the initial p_{esc} and p_{trap}. For $\mathbf{C}$ we initialised by a Gibbs block update given $\mathbf{z}$.

Convergence diagnostics. For the parameters, we ran multiple chains and assessed convergence using the Gelman point scale reduction factor (PSRF) (1). On real data we considered an MCMC run converged if the PSRF was less than 1.2 for all variables. We initialised the Markov chains by sampling from the prior distributions. Under the given prior parameters the initial values were over dispersed with respect to the target distributions on all trajectories (a requirement of the PSRF method).

Full derivation of Gibbs move for harmonic well centre

Here we calculate a Gibbs update for the harmonic well centre ($\mathbf{C}$). As described earlier in Note S1, we update in blocks of length n, $\mathbf{C}_{j,n} = \{C_i\}_{i=j}^{j+n}$, where $1 \le j \le N - n$. We derive an update by comparing the conditional distribution for a block (obtained from the posterior distribution, Equation (30) in the main text), and the multivariate normal PDF. On the one hand we have the conditional distribution for $\mathbf{C}_{j,n}$, for which there are two cases

$$\pi(\mathbf{C}_{j,n}|\mathbf{C}_{-j,n}, \mathbf{z}, \theta, \mathbf{X})\Big|_{j\neq 1} \propto \exp\left(\sum_{i=j}^{j+n} \frac{-\left(\Delta X_i - z_i(C_i - X_i + (X_i - C_i)e^{-\kappa\Delta t_i})\right)^2}{2D\left((1-z_i)2\Delta t_i + \frac{z_i}{\kappa}(1 - e^{-2\kappa\Delta t_i})\right)} + \sum_{i=j}^{j+n+1} \frac{-\Delta C_i^2}{4\Delta t_i(D_C z_i + D_{est}(1 - z_i))}\right) \tag{35}$$

$$\begin{aligned}
\pi(\mathbf{C}_{j,n}|\mathbf{C}_{-j,n}, \mathbf{z}, \theta, \mathbf{X})\Big|_{j=1} \propto \exp\Bigg(&\frac{-\tau_{C_1}}{2}\left(C_1 - \mu_{C_1}\right)^2 + \sum_{i=j}^{j+n} \frac{-\left(\Delta X_i - z_i(C_i - X_i + (X_i - C_i)e^{-\kappa\Delta t_i})\right)^2}{2D\left((1-z_i)2\Delta t_i + \frac{z_i}{\kappa}(1 - e^{-2\kappa\Delta t_i})\right)} \\
&+ \sum_{i=j}^{j+n+1} \frac{-\Delta C_i^2}{4\Delta t_i(D_C z_i + D_{est}(1 - z_i))}\Bigg)
\end{aligned} \tag{36}$$

where $\mathbf{C}_{-j,n} = \mathbf{C} \setminus \mathbf{C}_{j,n}$. On the other hand, the multivariate normal PDF with mean $\boldsymbol{\mu}_{C_{j,n}}$ and precision matrix $\boldsymbol{\Sigma}^{-1}_{C_{j,n}}$, up to proportionality with respect to $\mathbf{C}_{j,n}$, is

$$\begin{aligned}\exp\left[-\frac{1}{2}\left((\mathbf{C}_{j,n}-\boldsymbol{\mu}_{C_{j,n}})^T\boldsymbol{\Sigma}^{-1}_{C_{j,n}}(\mathbf{C}_{j,n}-\boldsymbol{\mu}_{C_{j,n}})\right)\right] &= \exp\left[-\frac{1}{2}\sum_{l=j}^{j+n}\sum_{m=j}^{j+n}(C_l-\mu_l)(C_m-\mu_m)\Sigma^{-1}_{l,m}\right]\\ &= \exp\left[-\frac{1}{2}\sum_{l=j}^{j+n}\sum_{m=j}^{j+n}(C_lC_m - C_l\mu_m - C_m\mu_l + \mu_l\mu_m)\Sigma^{-1}_{l,m}\right]\end{aligned} \tag{37}$$

where $\Sigma^{-1}_{l,m}$ denotes the (l,m)th element of the precision matrix and μ_l denotes the lth element of the mean vector. Thus we can calculate a multivariate normal update by comparing the coefficients in the exponential for Equations (35) and (37) (also using Equation (36) if $j=1$). For the squared and cross terms this gives

$$C_i^2\Big|_{2\le i\le N-1} : -\frac{1}{4\Delta t_{i-1}(D_C z_{i-1} + D_{est}(1-z_{i-1}))} - \frac{z_i(1-e^{\kappa\Delta t_i})^2}{2\frac{D}{\kappa}(1-e^{-2\kappa\Delta t_i})} - \frac{1}{4\Delta t_i(D_C z_i + D_{est}(1-z_i))} = -\frac{1}{2}\Sigma^{-1}_{i,i} \tag{38}$$

$$C_iC_{i-1} : \frac{1}{2\Delta t_{i-1}(D_C z_{i-1} + D_{est}(1-z_{i-1}))} = -\Sigma^{-1}_{i,i-1} = -\Sigma^{-1}_{i-1,i}. \tag{39}$$

And for $i=1$ and $i=N$ we have

$$C_1^2 : -\frac{\tau_{C_1}}{2} - \frac{z_i(1-e^{\kappa\Delta t_i})^2}{2\frac{D}{\kappa}(1-e^{-2\kappa\Delta t_i})} - \frac{1}{4\Delta t_1(D_C z_1 + D_{est}(1-z_1))} = -\frac{1}{2}\Sigma^{-1}_{1,1} \tag{40}$$

$$C_N^2 : -\frac{1}{4\Delta t_{N-1}(D_C z_{N-1} + D_{est}(1-z_{N-1}))} - \frac{z_i(1-e^{\kappa\Delta t_i})^2}{2\frac{D}{\kappa}(1-e^{-2\kappa\Delta t_i})} = -\frac{1}{2}\Sigma^{-1}_{N,N}. \tag{41}$$

By solving Equations (38)-(41) we can hence write the n by n precision matrix

$$\boldsymbol{\Sigma}^{-1}_{C_{j,n}} = \begin{pmatrix} \Sigma^{-1}_{j,j} & \Sigma^{-1}_{j,j+1} & & & \\ \Sigma^{-1}_{j+1,j} & \Sigma^{-1}_{j+1,j+1} & \Sigma^{-1}_{j+1,j+2} & & \\ & \Sigma^{-1}_{j+2,j+1} & \ddots & & \\ & & & \ddots & \\ & & & \ddots & \Sigma^{-1}_{j+n-1,j+n} \\ & & & \Sigma^{-1}_{j+n,j+n-1} & \Sigma^{-1}_{j+n,j+n} \end{pmatrix}. \tag{42}$$

The mean vector $\boldsymbol{\mu}_{C_{j,n}}$ can be calculated by comparing the C_i coefficients. For $j+1 \le i \le j+n-1$ this gives

$$C_i\Big|_{j+1\le i\le j+n-1} : \frac{z_i(1-e^{-\kappa\Delta t_i})^2)}{\frac{D}{\kappa}(1-e^{-2\kappa\Delta t_i})}\frac{\Delta X_i + X_i(1-e^{-\kappa\Delta t_i})}{1-e^{-\kappa\Delta t_i}} = \sum_{m=j}^{j+n}\mu_m\Sigma^{-1}_{i,m}. \tag{43}$$

And providing the block does not contain the first or last timepoint, we have

$$C_j : \frac{C_{j-1}}{2\Delta t_{j-1}(D_C z_{j-1} + D_{est}(1-z_{j-1}))} + \frac{z_j(1-e^{-\kappa\Delta t_j})^2)}{\frac{D}{\kappa}(1-e^{-2\kappa\Delta t_j})}\frac{\Delta X_j + X_j(1-e^{-\kappa\Delta t_j})}{1-e^{-\kappa\Delta t_j}} = \sum_{m=j}^{j+n}\mu_m\Sigma^{-1}_{j,m} \tag{44}$$

$$C_{j+n} : \frac{C_{j+n+1}}{2\Delta t_{j+n}(D_C z_{j+n} + D_{est}(1-z_{j+n}))} + \frac{z_{j+n}(1-e^{-\kappa\Delta t_{j+n}})^2)}{\frac{D}{\kappa}(1-e^{-2\kappa\Delta t_{j+n}})}\frac{\Delta X_{j+n} + X_{j+n}(1-e^{-\kappa\Delta t_{j+n}})}{1-e^{-\kappa\Delta t_{j+n}}} = \sum_{m=j}^{j+n}\mu_m\Sigma^{-1}_{j+n,m}. \tag{45}$$

If the block contains the first timepoint, i.e. $j=1$ we have

$$C_1 : \tau_{C_1}\mu_{C_1} + \frac{z_1(1-e^{-\kappa\Delta t_1})^2)}{\frac{D}{\kappa}(1-e^{-2\kappa\Delta t_1})}\frac{\Delta X_1 + X_1(1-e^{-\kappa\Delta t_1})}{1-e^{-\kappa\Delta t_1}} = \sum_{m=j}^{j+n}\mu_m\Sigma^{-1}_{1,m} \tag{46}$$

and if the block contains the last timepoint, i.e. $j+n=N$, then

$$C_N : \frac{z_i(1-e^{-\kappa\Delta t_i})^2)}{\frac{D}{\kappa}(1-e^{-2\kappa\Delta t_i})}\frac{\Delta X_i + X_i(1-e^{-\kappa\Delta t_i})}{1-e^{-\kappa\Delta t_i}} = \sum_{m=j}^{j+n}\mu_m\Sigma^{-1}_{N,m}. \tag{47}$$

To calculate $\boldsymbol{\mu}_{C_{j,n}}$ we solve the system of linear equations

$$\boldsymbol{\Sigma}^{-1}_{C_{j,n}}\boldsymbol{\mu}_{C_{j,n}} = \mathbf{b}_{j,n} \tag{48}$$

where $\mathbf{b}_{j,n}$ is a column vector with elements

$$b_i = \left\{\frac{z_i(1-e^{-\kappa\Delta t_i})^2)}{\frac{D}{\kappa}(1-e^{-2\kappa\Delta t_i})}\frac{\Delta X_i + X_i(1-e^{-\kappa\Delta t_i})}{1-e^{-\kappa\Delta t_i}}\right\}_{i=j+1}^{j+n-1} \tag{49}$$

and b_j, b_{j+n} from the left hand side of Equations (44) and (45). (Or Equation (46) or (47) if $j=1$ or $j+n=N$ respectively.) Since $\boldsymbol{\Sigma}^{-1}_{C_{j,n}}$ is tridiagonal this equation can be efficiently solved, for example using the left matrix division function (with $\boldsymbol{\Sigma}^{-1}_{C_{j,n}}$ as a sparse matrix) in Matlab. Given $\boldsymbol{\mu}_{C_{j,n}}$ and $\boldsymbol{\Sigma}^{-1}_{C_{j,n}}$ the Gibbs update is

$$\mathbf{C}_{j,n} \sim N(\boldsymbol{\mu}_{C_{j,n}}, \boldsymbol{\Sigma}^{-1}_{C_{j,n}}). \tag{50}$$

Pseudocode for harmonic potential confinement HMM simulation

In the simulations we include a drift term for the centre so that C tracks X when not confined; this ensures that when the particle switches from free diffusion to confinement the well centre C is close to X which is a realistic requirement. This allows for confinement zones to be small relative to the field of view. This tracking of X by C isn't included in the inference algorithm as diffusion alone is sufficient to allow the MC to find appropriate paths.

Algorithm 2 Simulation algorithm for harmonic potential well model.

$\{D, D_{est}, D_C, \kappa, p_{esc}, p_{trap}\} \leftarrow$ choice of model parameters
$\{\Delta t_i\}_{i=1}^{N+1} \leftarrow$ choice of time steps ($\Delta t_i = t_{i+1} - t_i$)
$X_1 \leftarrow$ initial particle position
$C_1 \leftarrow$ initial harmonic well centre position
$z_1 \leftarrow$ initial confinement state
for $i=1$ to $i=N-1$ **do**
  $z_{i+1} \leftarrow$ random number drawn from Bernoulli$(z_i(1-p_{esc})) + (1-z_i)p_{trap})$
  $C_{i+1} \leftarrow$ random number drawn from $N\left(C_i + \kappa\Delta t_i(1-z_i)(X_i - C_i), 2\Delta t_i(D_C z_i + D_{est}(1-z_i))\right)$
  $X_{i+1} \leftarrow$ random number drawn from $N\left(X_i + z_i(C_i - X_i + (X_i - C_i)e^{-\kappa\Delta t_i}), D\left((1-z_i)2\Delta t_i + \frac{z_i}{\kappa}(1-e^{-2\kappa\Delta t_i})\right)\right)$
end for
$X_{N+1} \leftarrow$ random number drawn from $N\Big(X_N + z_N(C_N - X_N + (X_N - C_N)e^{-\kappa\Delta t_N}),$
$D\left((1-z_N)2\Delta t_N + \frac{z_N}{\kappa}(1-e^{-2\kappa\Delta t_N})\right)\Big)$

2 Note S2: Data preprocessing

The initial analysis of this dataset (2) revealed superdiffusive behaviour at time delays less than 1×10^{-4} s, which was attributed to dynamic error in measurements due to sub-nanometre localisation precision. An analysis revealed that displacement angles showed a bias towards horizontal displacements (Fig. S1). We determined that this behaviour was a result of the raster scan whereby there was an inaccuracy in the x coordinate at these fast recording rates. This effect was statistically significant in a Chi-squared test (with the null hypothesis that angular displacements follow a uniform distribution), Fig. S2.

This bias was removed, in $40\,\mathrm{nm}$ AuNP/CTxB/GM1 trajectories on mica, by subsampling at rate 10, Fig. S2A. These trajectories were previously shown to display no trapping (2); hence the bias that remains even when subsampling at higher rates in displacement angles for $20\,\mathrm{nm}$ AuNP/CTxB/GM1 on glass trajectories is presumably due to directional displacements in trapping events, Fig. S2A. We also investigated the effect of window averaging, taking the average particle position over a window size n, and found similar trends to subsampling, Fig. S2B. This analysis therefore indicated that a subsampling rate of 10 is sufficient to remove these spatial localisation artifacts. This reduces the trajectory sampling rate from $50\,\mathrm{kHz}$ to $5\,\mathrm{kHz}$.

Additionally, a small number of $20\,\mathrm{nm}$ AuNP/CTxB/GM1 trajectories on glass (7 out of 71) had displacements that were unreasonably large, probably caused by an additional AuNP in the focal area. We dealt with this by visually inspecting the trajectories, and removing (before subsampling) the section of the trajectory with the artifacts. All presented analysis is on this subsampled, quality assured set.

Supporting References

1. Gelman, A., J. B. Carlin, H. S. Stern, D. B. Dunson, A. Vehtari, and D. B. Rubin, 2013. Bayesian Data Analysis, Third Edition. CRC Press.
2. Spillane, K. M., J. Ortega-Arroyo, G. de Wit, C. Eggeling, H. Ewers, M. I. Wallace, and P. Kukura, 2014. High-Speed Single-Particle Tracking of GM1 in Model Membranes Reveals Anomalous Diffusion due to Interleaflet Coupling and Molecular Pinning. *Nano Letters* 14:5390–5397.

1 Table S1: Correlations between confinement event statistics.

	Event lifetime	Mean confinement radius	Radial skewness
Mean confinement radius	0.01 ($p = 0.81$)		
Radial skewness	-0.13 ($p = 0.0003$)	-0.28 ($p < 0.0001$)	
Radial mean-median distance	-0.16 ($p < 0.0001$)	0.29 ($p < 0.0001$)	0.26 ($p < 0.0001$)
Radial SD	-0.09 ($p = 0.01$)	0.69 ($p < 0.0001$)	0.12 ($p = 0.0006$)

Pearson correlation coefficient between confinement statistics, for the set of all confinement events, except those which contained either the first or last timepoint. Exact calculations for confinement statistics are also given in Table 1, main text.

2 Table S2: Comparison of within and between trajectory variances for confinement event statistics.

Statistic	Mean within trajectory variance	Variance across all events	ANOVA p-value
Mean confinement radius ($\bar{R}_{lm}$)	$3.1 \times 10^{-5}\,\mu m^2$	$4.8 \times 10^{-5}\,\mu m^2$	1.2×10^{-6}
Radial skewness (S_{lm})	0.49	0.77	9.1×10^{-4}
Radial mean-median distance	$2.9 \times 10^{-7}\,\mu m^2$	$4.4 \times 10^{-7}\,\mu m^2$	0.06
Radial SD	$4.5 \times 10^{-6}\,\mu m^2$	$4.9 \times 10^{-6}\,\mu m^2$	3.0×10^{-30}
Confinement event lifetime	$0.025\,s^2$	$0.029\,s^2$	2.6×10^{-19}

Spatial statistics calculated on the set of 271 events obtained by applying the restrictions given in Table 1 in the main text. Lifetime statistics calculated on set of 214 events, also described in Table 1, main text. ANOVA calculated across all trajectories, not including events revisiting the same location.

1 Fig. S1: Radial histogram of angular displacements for a 40 nm AuNP/CTxB/GM1 trajectory on mica.

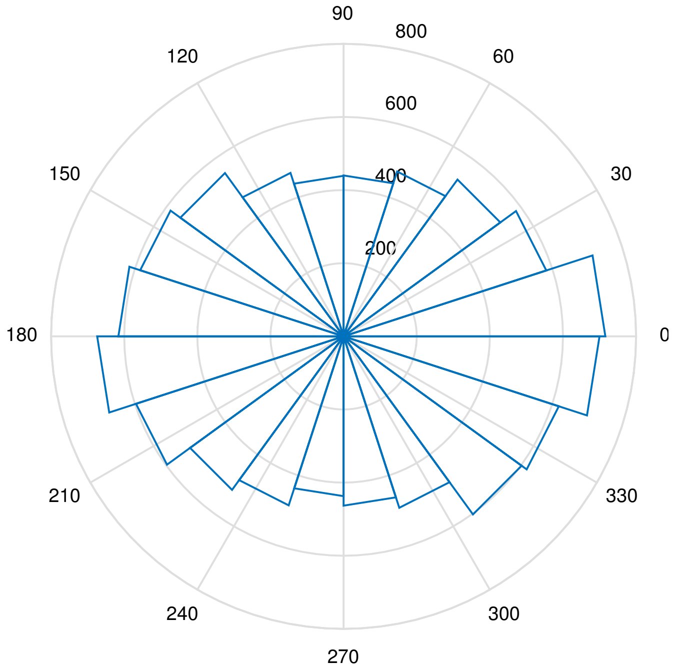

The angular displacement (plotted in degrees) in radians is $\phi_i = \arctan(\Delta X_{2i}/\Delta X_{1i}) + \pi$, where $\Delta X_i = \{\Delta X_{1i}, \Delta X_{2i}\}$.

2 Fig. S2: Subsampling removes the angular bias in trajectory displacements.

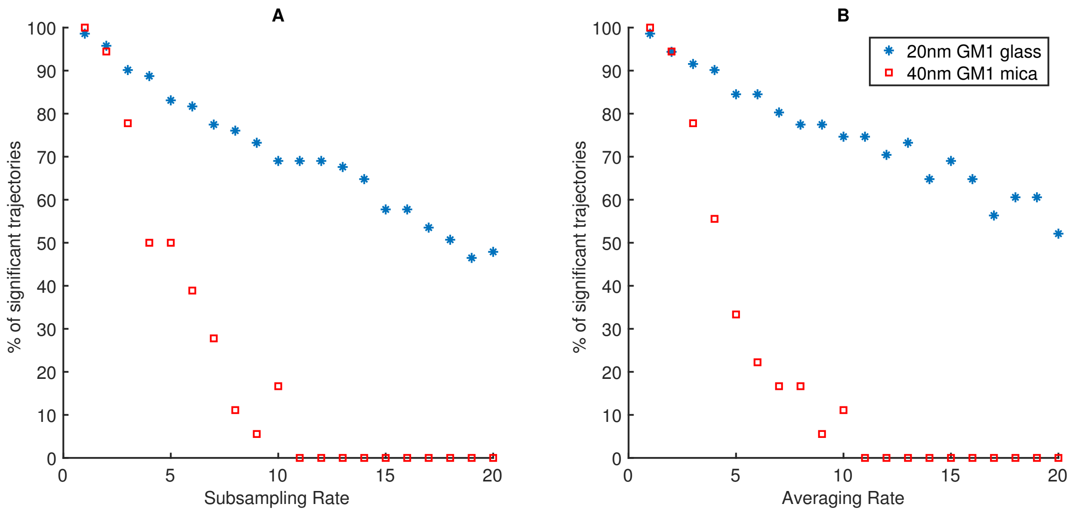

Percentage of GM1 trajectories where the null hypothesis (angular displacements follow a uniform distribution) was rejected in a Chi-square test, plotted against subsampling (A) or averaging rate (B). For each trajectory, the null hypothesis was rejected if $p < 0.0003$ (p=0.05 with Bonferroni correction, 169 trajectories in total).

3 Fig. S3: Confinement probabilities cluster around 0 and 1.

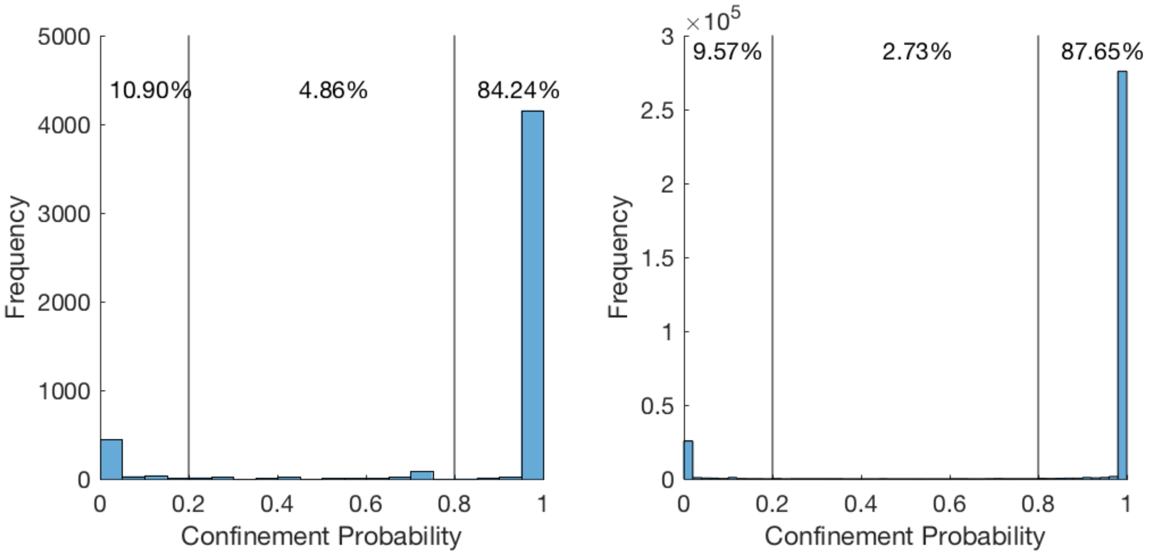

Histograms of inferred (posterior) probabilities for confinement state $\mathbf{z}$ on $20\,\mathrm{nm}$ AuNP/CTxB/GM1 trajectories. (A) Single representative trajectory. (B) Pooled probabilities across all trajectories (66). Percentages report the weight in each section (0-0.2, 0.2-0.8 and 0.8-1).

4 Fig S4: Fit of HPW model to a simulated trajectory

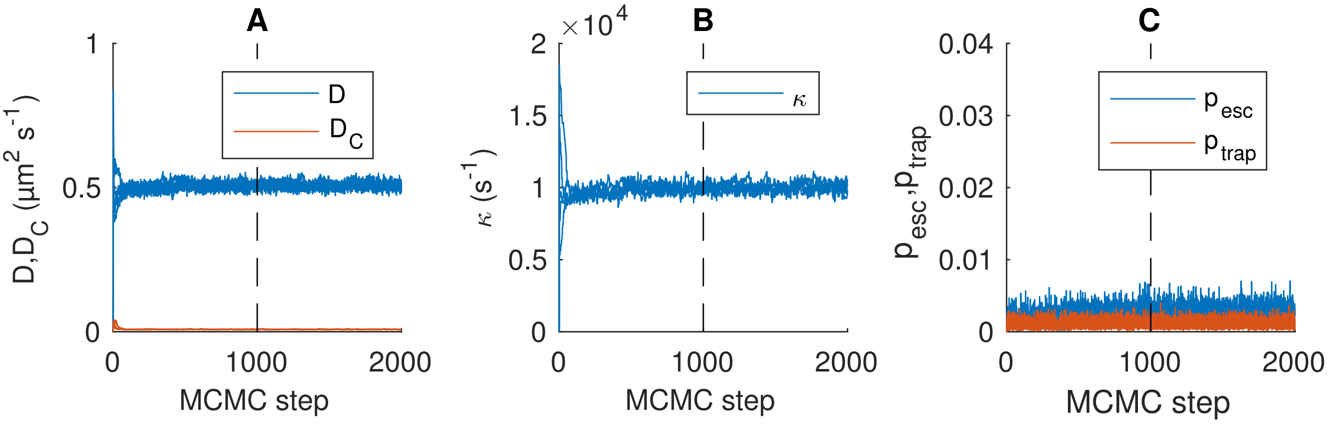

(A) MCMC chains for D (blue) and D_C (red). (B) MCMC chain for κ. (C) MCMC chains for p_{esc} and p_{trap}. For each parameter 12 independent MCMC runs are shown. Corresponding parameter posterior distributions are shown in Fig. 2 in the main text.

5 Fig. S5: Varying D_{est} in simulations: parameter estimates

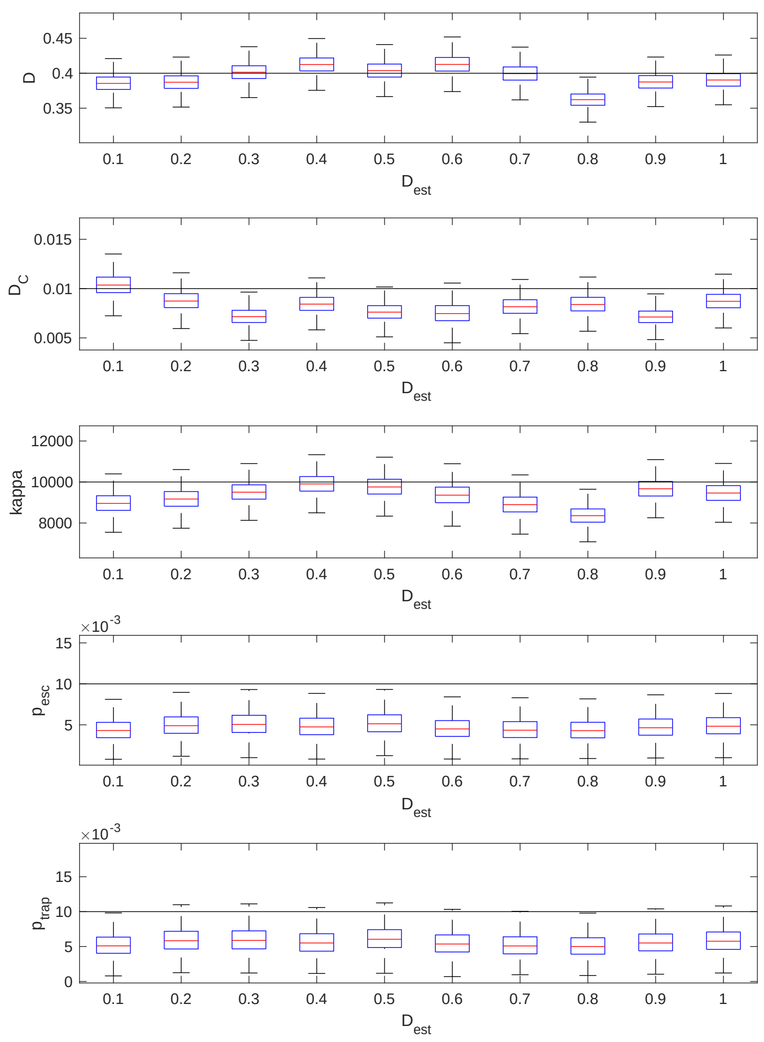

Parameter posterior samples for the harmonic potential well model applied to simulated trajectories with varying D_{est} values. Other simulation parameters: $D = 0.4\,\mu\mathrm{m}^2\,\mathrm{s}^{-1}$, $D_C = 0.01\,\mu\mathrm{m}^2\,\mathrm{s}^{-1}$, $\kappa = 10\,000\,\mathrm{s}^{-1}$, $p_{esc} = 0.01$, $p_{trap} = 0.01$.

6 Fig. S6: Varying D_{est} in simulations with localisation error: parameter estimates

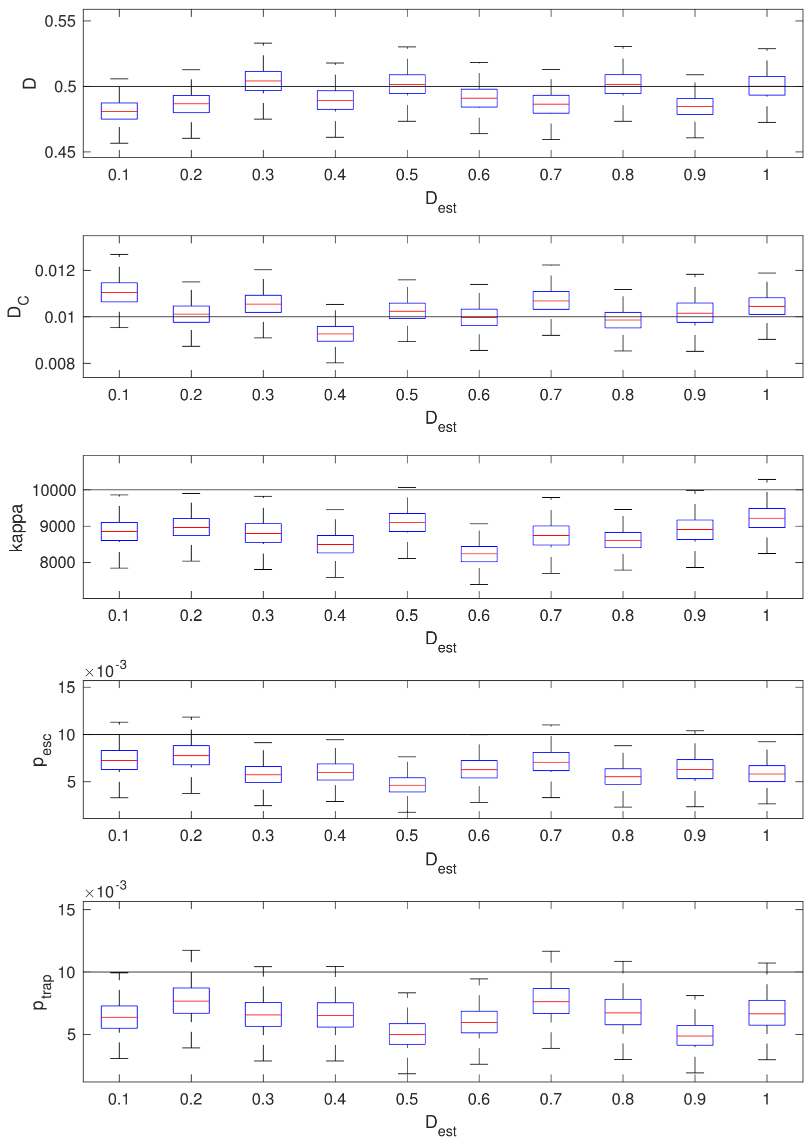

Parameter posterior samples for the harmonic potential well model applied to simulated trajectories with varying D_{est} values. Trajectories include static localisation (measurement) error with standard deviation $2.7\,\mathrm{nm}$, and were preprocessed by subsampling at rate 10. Other simulation parameters: $D = 0.5\,\mathrm{\mu m^2\,s^{-1}}$, $D_C = 0.01\,\mathrm{\mu m^2\,s^{-1}}$, $\kappa = 10\,000\,\mathrm{s^{-1}}$, $p_{esc} = 0.01$, $p_{trap} = 0.01$.

7 Fig. S7: Varying static localization error in simulations

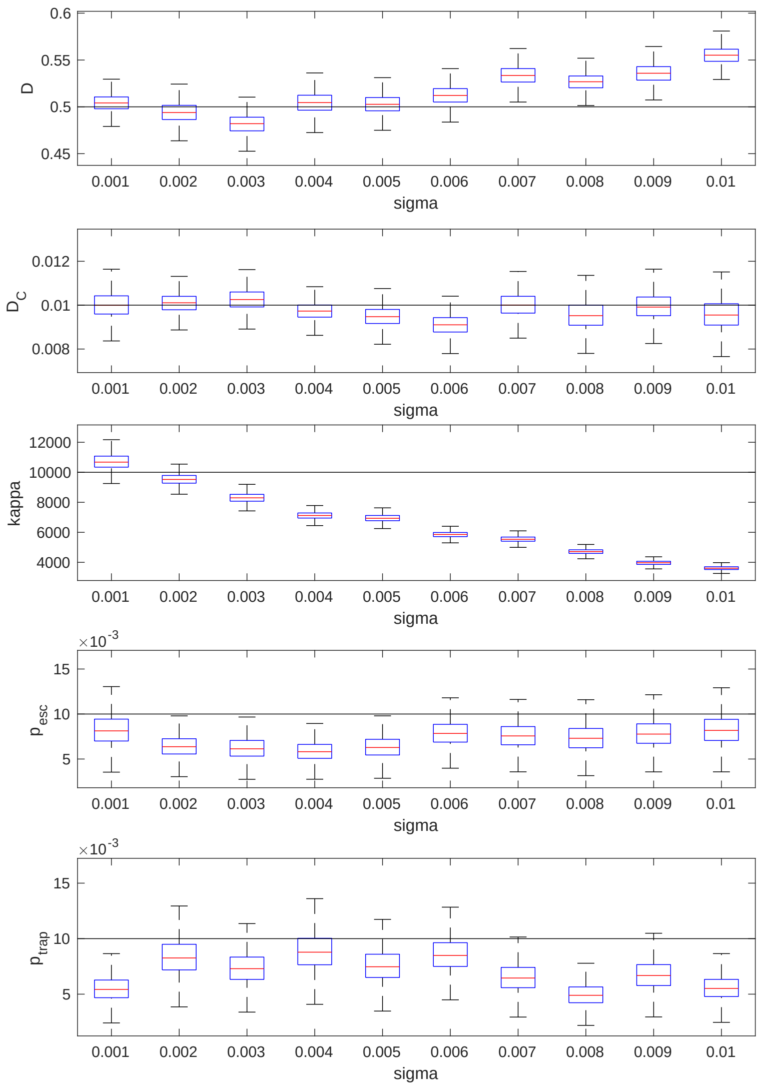

Parameter posterior distributions for harmonic potential well model applied to simulated trajectories including static localisation error with varying standard deviation σ (in microns). Trajectories were preprocessed by subsampling at rate 10. Black lines represent simulated parameter values ($D = 0.5\,\mu\mathrm{m}^2\,\mathrm{s}^{-1}$, $D_C = 0.01\,\mu\mathrm{m}^2\,\mathrm{s}^{-1}$, $\kappa = 10\,000\,\mathrm{s}^{-1}$, $p_{esc} = 0.01$, $p_{trap} = 0.01$).

8 Fig. S8: Varying trajectory length in simulations

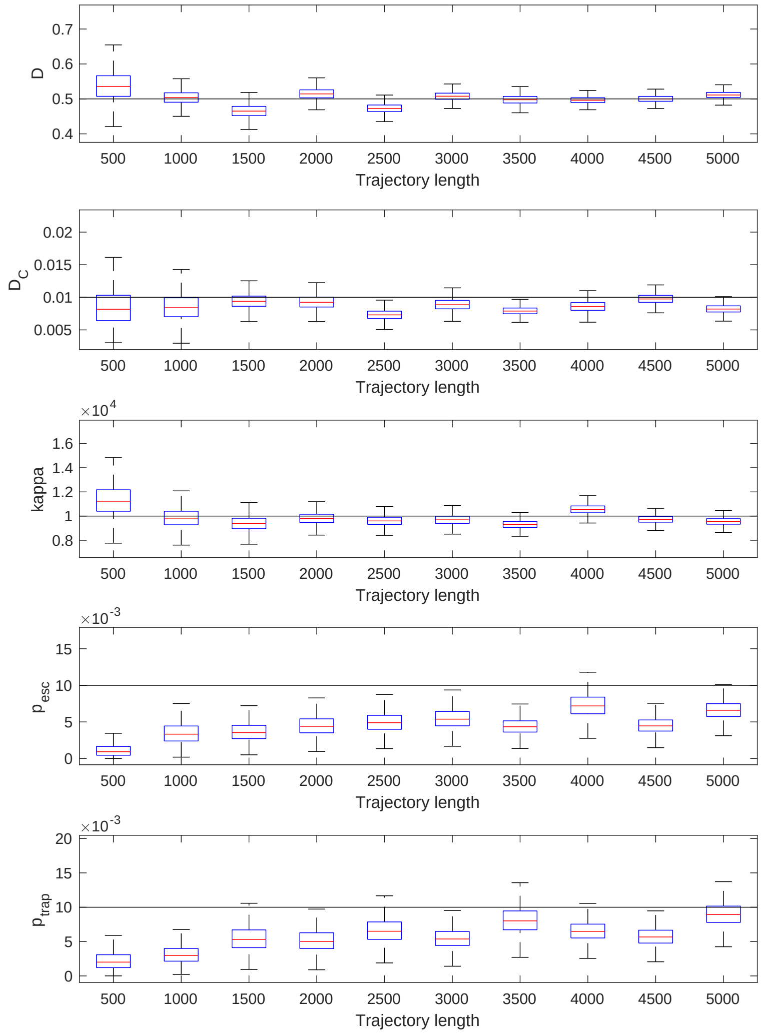

Parameter posterior samples for harmonic potential well model applied to simulated trajectories of varying length (between 500 and 5000 time steps). Black lines represent the simulated parameter values ($D = 0.5\,\mu\text{m}^2\,\text{s}^{-1}$, $D_C = 0.01\,\mu\text{m}^2\,\text{s}^{-1}$, $\kappa = 10\,000\,\text{s}^{-1}$, $p_{esc} = 0.01$, $p_{trap} = 0.01$).

9 Fig. S9: Varying number of confinement events in simulations

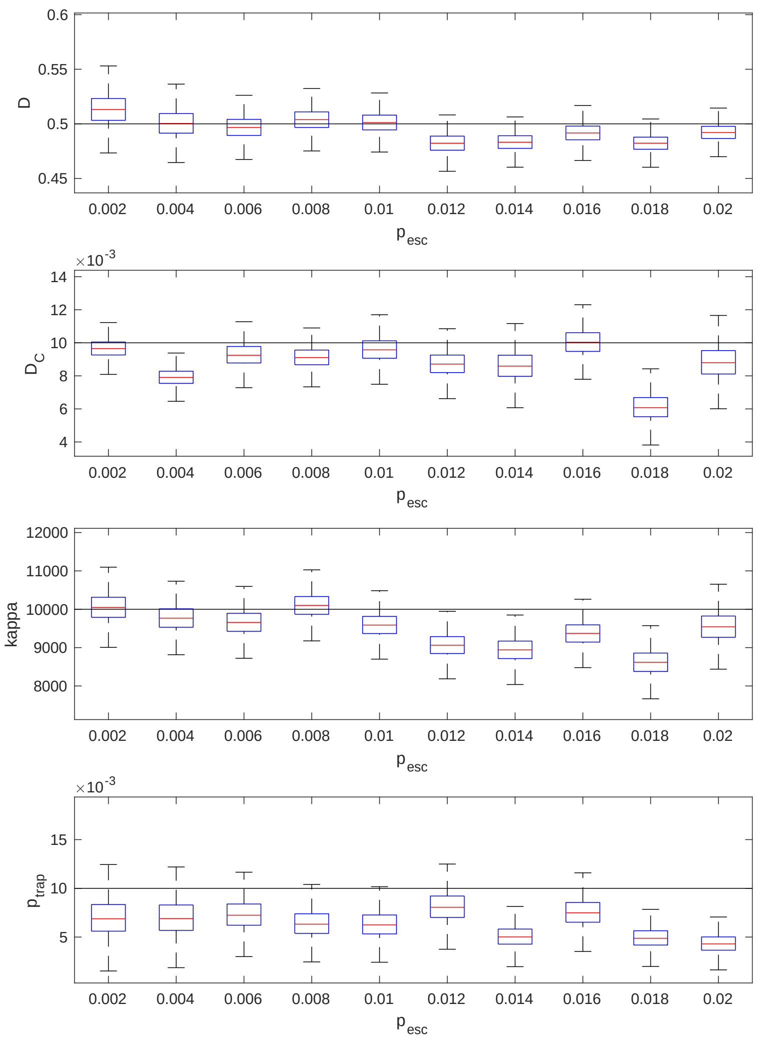

Parameter posterior distributions for harmonic potential well model applied to simulated trajectories with varying confinement event length. The event length was changed by adjusting the p_{esc} parameter between 0.002 and 0.02. Black lines represent simulated parameter values ($D = 0.5\,\mu\text{m}^2\,\text{s}^{-1}$, $D_C = 0.01\,\mu\text{m}^2\,\text{s}^{-1}$, $\kappa = 10\,000\,\text{s}^{-1}$, $p_{esc} = 0.01$, $p_{trap} = 0.01$).

10 Fig. S10: Varying subsampling rate in simulations: parameter estimates

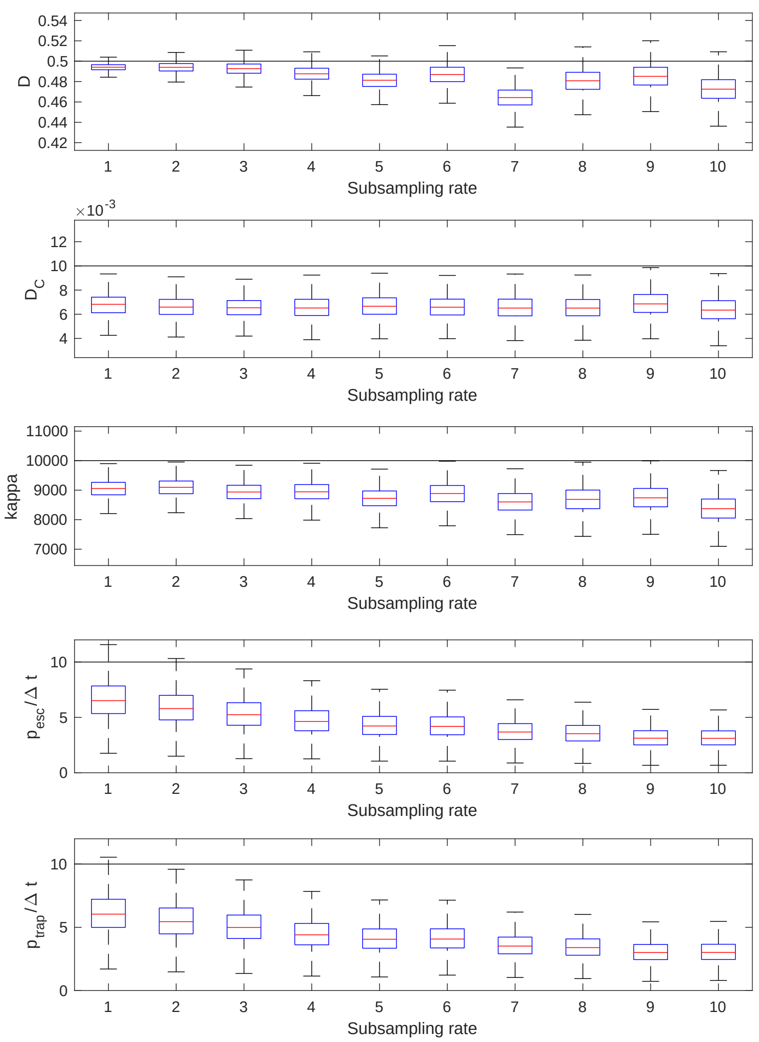

Parameter posterior samples for the harmonic potential well model applied to a single simulated trajectory (total length 20000 time steps) subsampled at different rates. We plot the rates, $p_{esc}/\Delta t$ and $p_{trap}/\Delta t$, instead of transition probabilities as these are invariant to the subsampling rate. Black lines represent simulated parameter values ($D = 0.5\,\mu\mathrm{m}^2\,\mathrm{s}^{-1}$, $D_C = 0.01\,\mu\mathrm{m}^2\,\mathrm{s}^{-1}$, $\kappa = 10\,000\,\mathrm{s}^{-1}$, $p_{esc}/\Delta t = 10\,\mathrm{s}^{-1}$, $p_{trap}/\Delta t = 10\,\mathrm{s}^{-1}$).

11 Fig. S11: Varying subsampling rate in simulations: confinement state

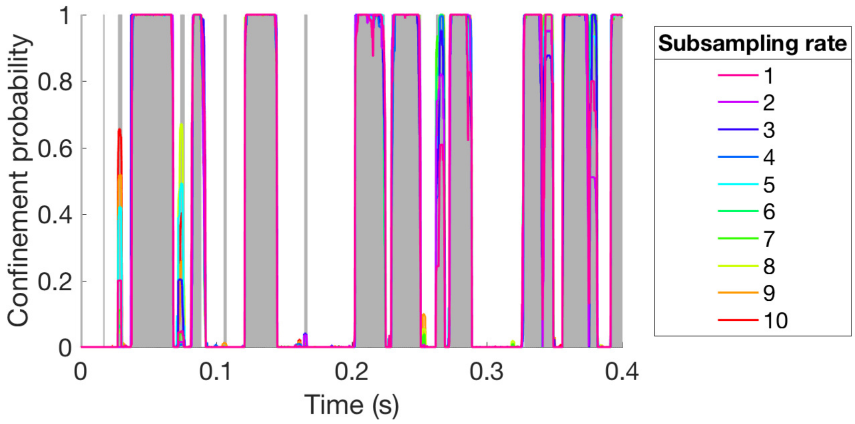

Confinement state posterior estimates for the model applied to a single simulated trajectory (total length 20000 time steps) subsampled at different rates. Grey area denotes the simulated (true) confinement state. Simulation parameters: $D = 0.5\,\mu\text{m}^2\,\text{s}^{-1}$, $D_C = 0.01\,\mu\text{m}^2\,\text{s}^{-1}$, $\kappa = 10\,000\,\text{s}^{-1}$, $p_{esc} = 0.002$, $p_{trap} = 0.002$.

12 Fig. S12: Varying confinement strength in simulations

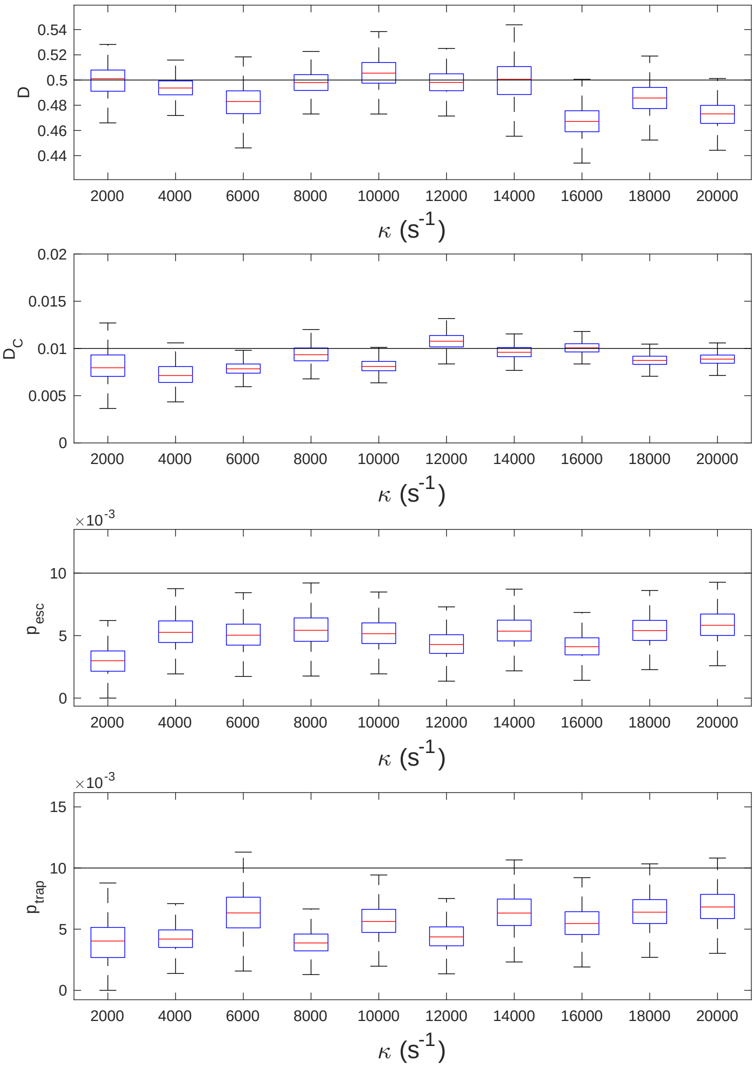

Parameter posterior distributions for harmonic potential well model applied to simulated trajectories with varying confinement strength κ. Black lines represent simulated parameter values ($D = 0.5\,\mu m^2\,s^{-1}$, $D_C = 0.01\,\mu m^2\,s^{-1}$, $p_{esc} = 0.01$, $p_{trap} = 0.01$).

13 Fig. S13: Varying confinement centre diffusion coefficient in simulations

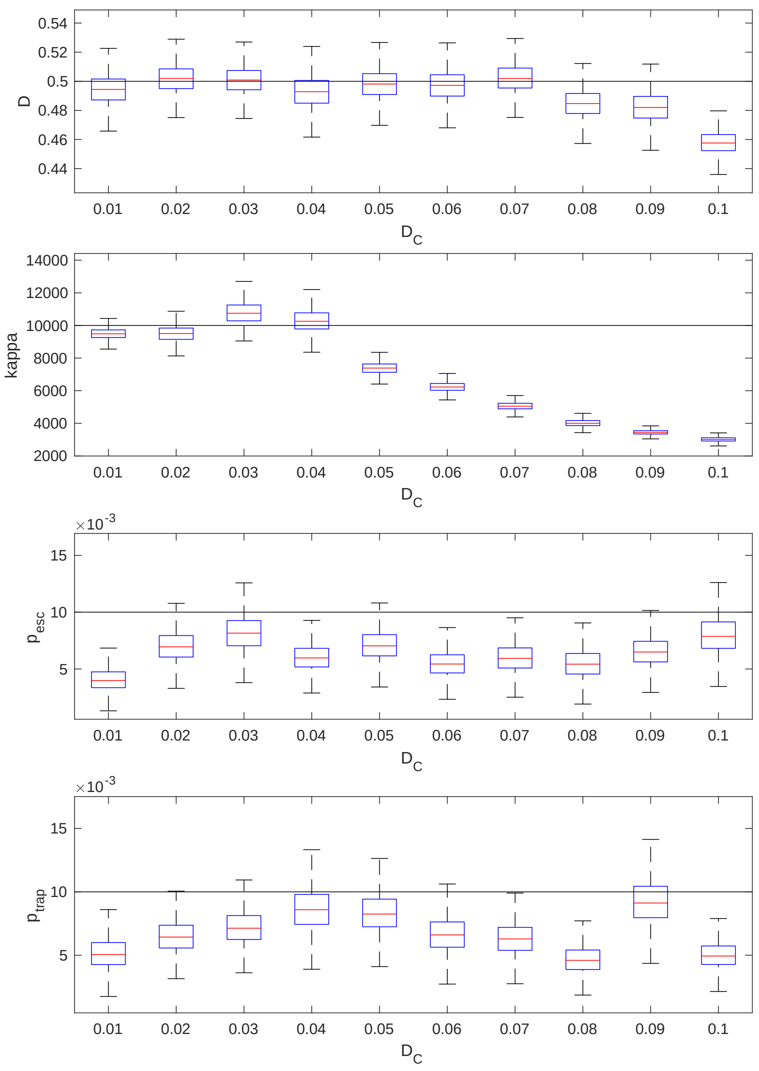

Parameter posterior distributions for harmonic potential well model applied to simulated trajectories with varying confinement centre diffusion coefficient D_C. Black lines represent simulated parameter values ($D = 0.5\,\mu\mathrm{m}^2\,\mathrm{s}^{-1}$, $\kappa = 10\,000\,\mathrm{s}^{-1}$, $p_{esc} = 0.01$, $p_{trap} = 0.01$).

14 Fig. S14: Fit of HPW model to a 20 nm AuNP/CTxB/GM1 trajectory in a model membrane on glass.

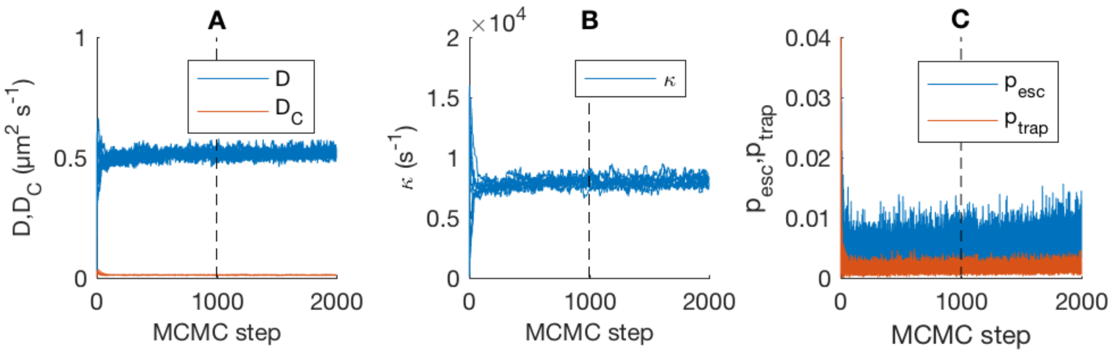

(A) MCMC chains for D (blue) and D_C (red). (B) MCMC chain for κ. (C) MCMC chains for p_{esc} and p_{trap}. For each parameter 12 independent MCMC runs are shown. Corresponding parameter posterior distributions are shown in Fig. 5 in the main text.

Fig. S15: 3D representation of HPW model applied to a 20 nm AuNP/CTxB/GM1 trajectory.

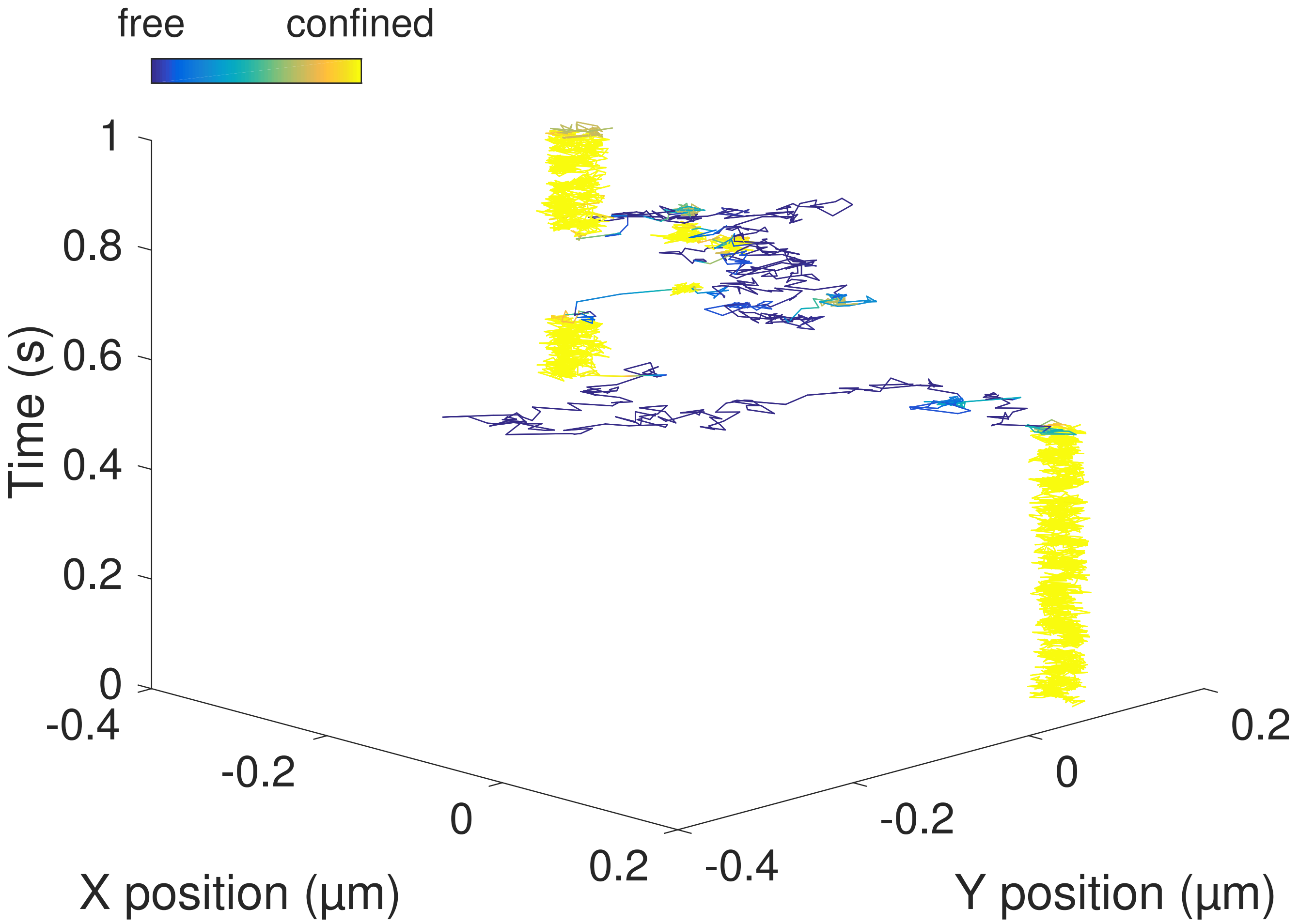

Colour represents the inferred hidden state. Corresponding hidden state and parameter posteriors are shown in Figs. 4 and 5 (main text) respectively.

16 Fig. S16: Parameter distributions across the population of trajectories.

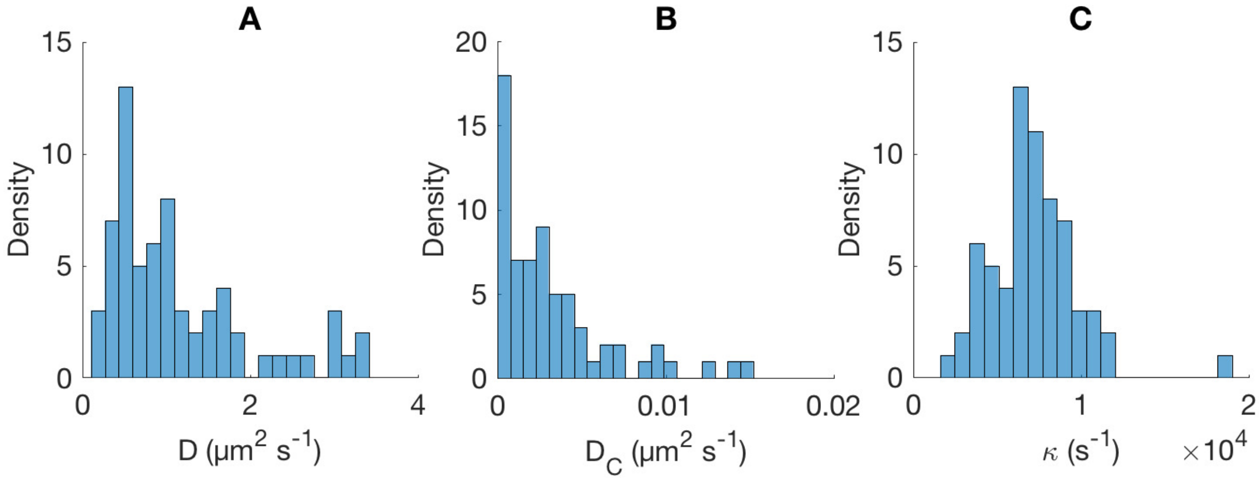

Histogram of the mean (posterior) parameters for HPW model applied to 20 nm AuNP/CTxB/GM1 on glass trajectories. A) Particle diffusion coefficient, B) harmonic well centre diffusion coefficient, C) harmonic well strength. D shows distinct deviation from being Gaussian ($p < 0.001$, Lilliefors test), although κ does not ($p = 0.21$).

17 Fig. S17: Variation in parameter posteriors across trajectories.

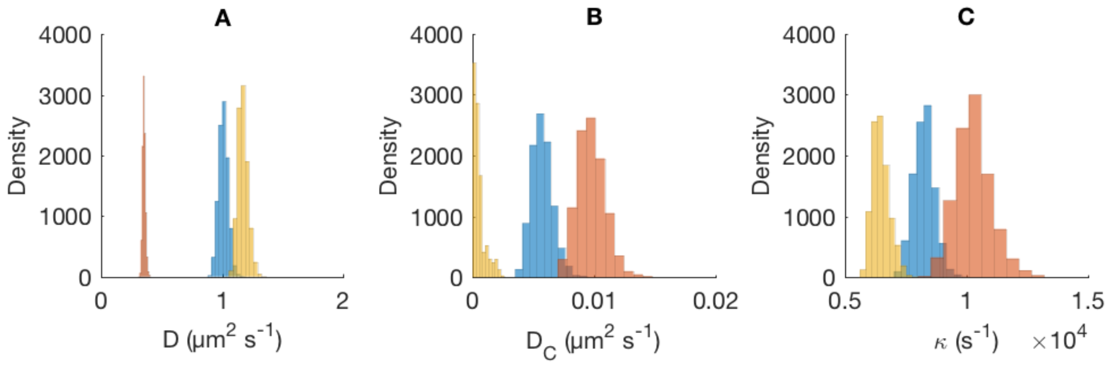

Histogram of parameter posterior samples for HPW model applied to three 20 nm AuNP/CTxB/GM1 on glass trajectories. A) Particle diffusion coefficient, B) harmonic well centre diffusion coefficient, C) harmonic well strength.

18 Fig. S18: Pooled confinement histograms for all 20 nm AuNP/CTxB/GM1 trajectories ordered by confinement size.

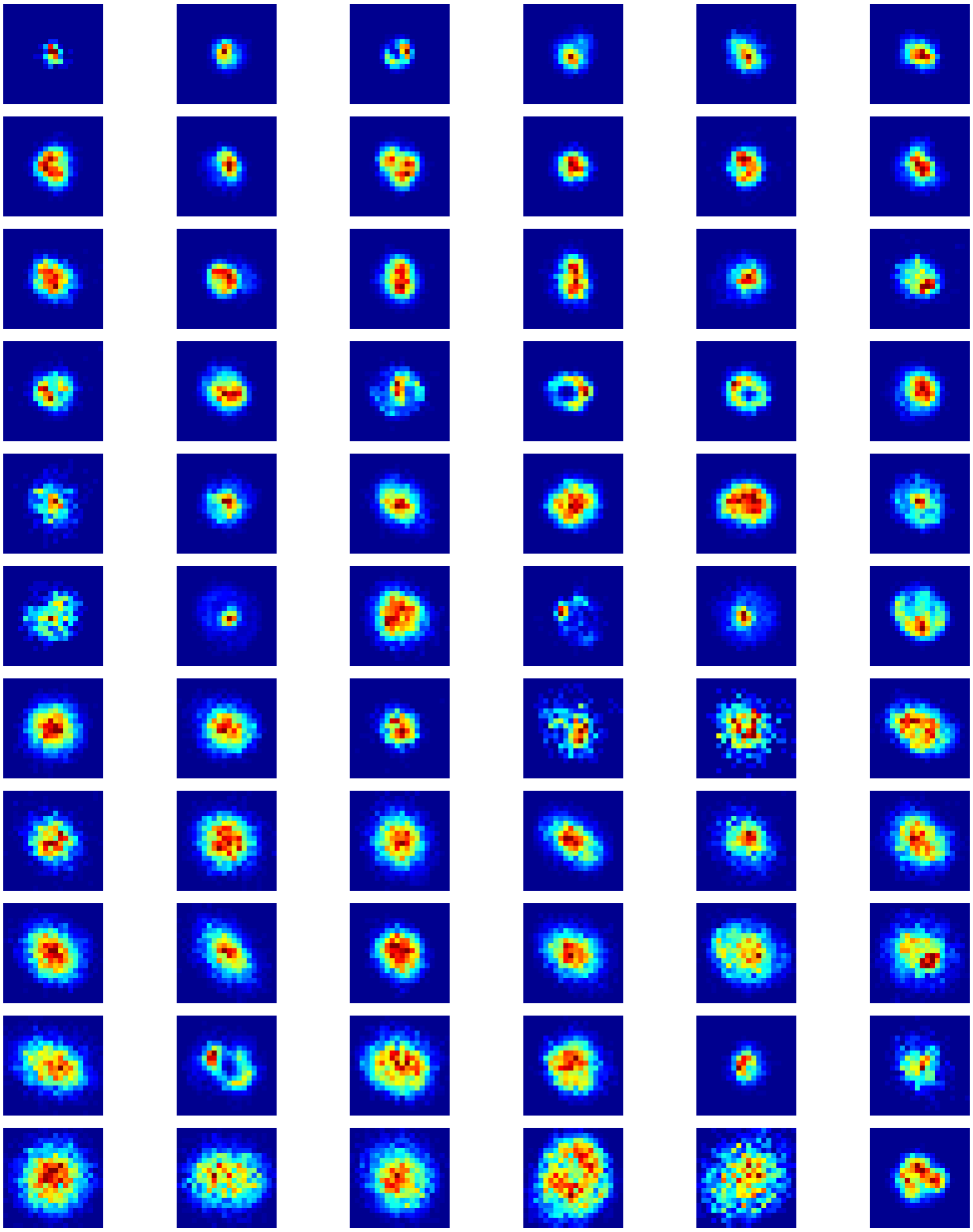

Histograms of particle positions pooled over confinement events for a trajectory. Trajectories are ordered (from smallest to largest) by the average of the mean confinement radius statistic ($\bar{R}_{lm}$) over all events (i.e. the order in Fig. 7C, main text). Confinement events were included based on the criteria in Table 1 in the main text, except that we included events revisiting a previous trapping zone (i.e. we did not enforce the condition that confinement event centres have to be $30\,\mathrm{nm}$ away from all previous centres in that trajectory). Each plot has side length $0.1\,\mu\mathrm{m}$.

19 Fig. S19: Pooled confinement histograms for all 20 nm AuNP/CTxB/GM1 trajectories ordered by spatial skew.

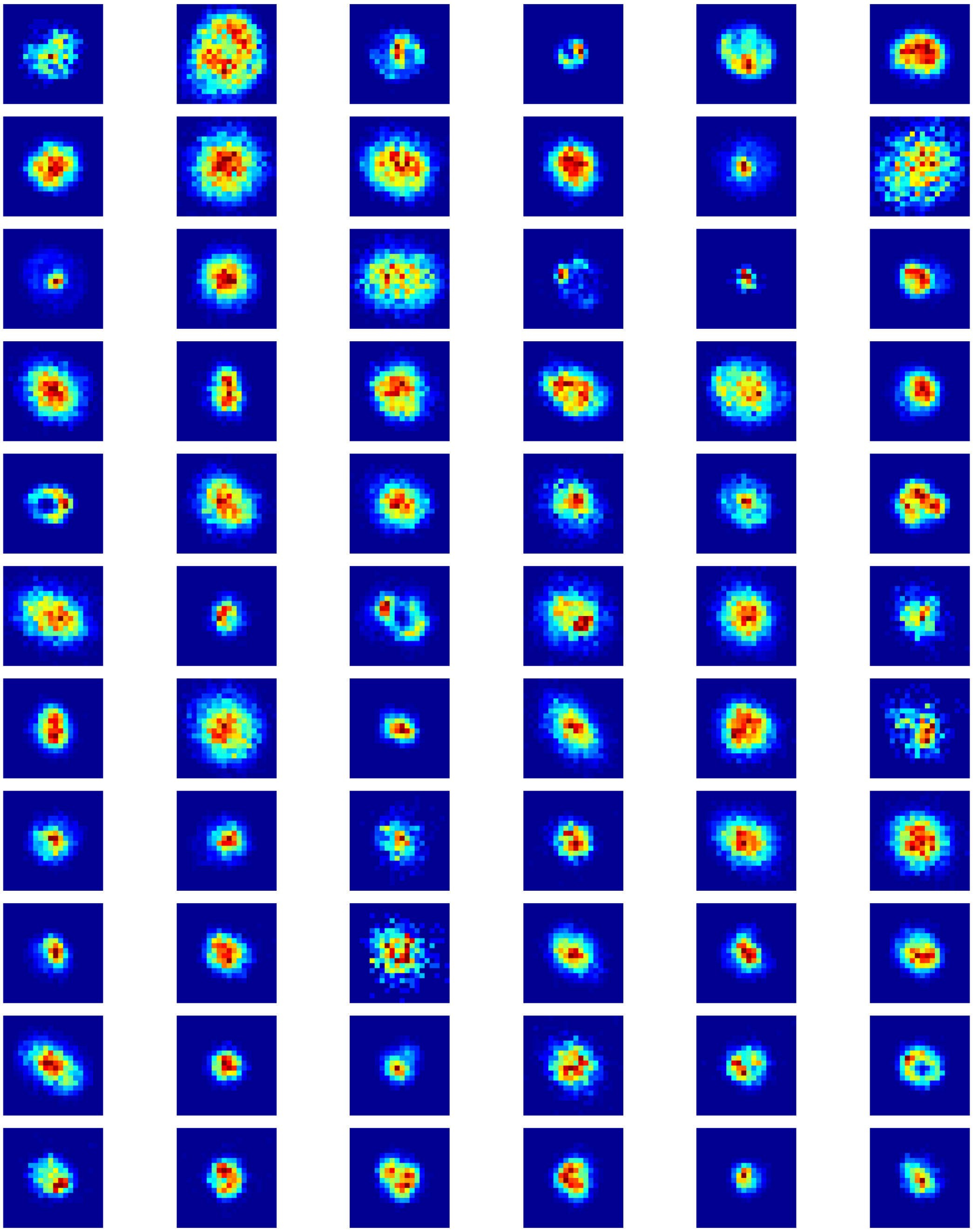

Histograms of particle positions pooled over confinement events for a trajectory. Trajectories are ordered (from smallest to largest) by the average of the radial skewness statistic (S_{lm}) over all events (i.e. the order in Fig. 7D, main text). Confinement events were included based on the criteria in Table 1 in the main text, except that we included events revisiting a previous trapping zone (i.e. we did not enforce the condition that confinement event centres have to be $30\,\mathrm{nm}$ away from all previous centres in that trajectory). Each plot has side length $0.1\,\mu\mathrm{m}$.

20 Fig. S20: GM1 trajectories in SLBs on a mica substrate show no confinement.

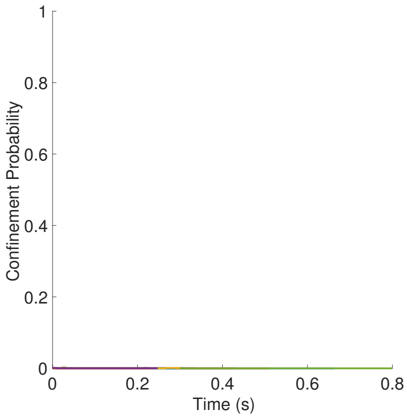

Inferred mean confinement state (average from 12 MCMC chains) from the harmonic potential well model applied to $40\,\mathrm{nm}$ AuNP/CTxB/GM1 trajectories in SLBs on mica. 18 trajectories of varying lengths (0.16-0.8s) each plotted in a different colour. The confinement (posterior) probability in all trajectories, was practically 0 at all times.
